# Supplementary material for: Trial of intraoperative cell salvage versus transfusion in ovarian cancer (TIC TOC): protocol for a randomised controlled feasibility study
Source: BMJ Open. 2018 Nov 1;8(11):e024108. doi: 10.1136/bmjopen-2018-024108 (PMC6224724; doi:10.1136/bmjopen-2018-024108)
Supplement: Supplementary data [file bmjopen-2018-024108supp001.pdf]

Appendix 1: Participant Information Sheet

TIC TOC

Trial of intraoperative cell salvage versus transfusion in ovarian cancer – a feasibility study (the TIC TOC study)

Participant Information Sheet

We'd like to invite you to take part in our research study

- Before you decide whether to take part it is important for you to understand why the research is being done and what it would involve for you.
- Please take time to read the following information carefully. Discuss it with your family, friends or your family doctor (GP) if you wish.
- You are free to decide whether or not to take part in this study. If you choose not to take part, this will not affect the care you get from your doctors.
- Please ask us if anything is not clear, or if you would like [illegible]

Important information about this study

- This is a **feasibility study.** A feasibility study may be carried out before a main study in order to answer the question "Can this study be done?"
- The aim of this study is to find out whether we can successfully plan and carry out a larger study in the future.
- If you take part in this study you will be randomly allocated to receive **either** a reinfusion (return) of your own blood (called Intraoperative Cell Salvage) **or** a transfusion of donated blood (standard blood transfusion), if there is enough blood loss during your forthcoming operation.
- Your care and medications will continue as normal.
- The study involves completing some follow-up questionnaires which will be sent to you by post.

Key contents

	Page
What is the purpose of the study?	2
Donor blood transfusion	2
Intraoperative cell salvage (ICS)	2
Why have I been approached?	2
What does the study involve?	2
Do I have to take part?	4
Study flowchart	5
What are the possible risks?	6
General information	7

If you have any questions about this study please contact:

Your research nurse:

<Enter local contact name>

>Enter local contact details<

What is the purpose of the study?

During any surgical operation it is common for there to be some loss of blood. Sometimes this is significant enough for the patient to need a blood transfusion. Giving someone a blood transfusion involves transfusing donated blood from an anonymous healthy donor.

For a long time, donor blood has been the only choice available for blood transfusion. Although this is a very safe procedure in the UK it is not completely without risk. There is now a technique available in which blood lost by a patient during surgery is collected, washed and given back to the same patient. The technical name for this is **Intraoperative Cell Salvage** (ICS).

Donor blood transfusion

- Blood from an anonymous donor
- Washed and filtered in the blood bank

ICS blood reinfusion (ICS blood return)

- Patient's own blood
- Washed and filtered during the operation

Surgery is one of the main treatments for ovarian cancer. ICS blood return is already used successfully in other types of surgery and is being used in cancer surgery including some ovarian cancer operations. There is some evidence that using ICS blood return instead of donor blood transfusion promotes better recovery for patients after surgery. However, we do not know which method is better for patients undergoing ovarian cancer surgery. We also do not know which method is better value for money. This study will help us to answer those questions.

Why have I been approached to take part?

You are being invited to take part because you are due to have surgery for ovarian cancer, or suspected ovarian cancer, at one of the four participating hospitals and have been identified as being potentially suitable for the study. You cannot take part if: -

- You have any other diagnosed cancer
- You are pregnant
- You have any disease of the red blood cells such as sickle cell or thalassaemia
- You are unwilling to accept donor blood (e.g. on religious grounds)

What does the study involve?

This study will involve 60 women in total. Half of the women taking part in the study will be allocated to receive a donor blood transfusion. The other half will be allocated to receive an ICS blood return. All women will receive a brief telephone check-up by a research nurse approximately one month after surgery, and will also be asked to complete up to four study follow-up questionnaire booklets by post. There are more details about this on page 4. There are **no** extra hospital visits required.

What will happen to me if I agree to take part in the study?

If you agree to take part in the study after considering the information provided, you will be asked to sign a consent form before any study procedures are completed. Consent will usually be taken when you attend the routine pre-operative assessment clinic. A research nurse will then review your past and current health status and you will be asked to complete a questionnaire booklet about your general health and wellbeing, and how your illness is affecting your daily life. When you have your operation, you may be given blood replacement by either a donor blood transfusion (if there is enough blood loss) or you will be given an ICS blood return. The blood replacement you receive will depend on which method you are allocated. Some women may not require any blood replacement at all.

Who decides which type of blood replacement I receive?

If you consent to take part in the study, you will be allocated at random (by chance – like tossing a coin) by computer to receive either ICS blood return during your operation, or, if replacement blood is needed, a donor blood transfusion. This is called **randomisation**. All other aspects of your operation and care will be exactly the same as if you had decided not to be involved in the study. During the study, you will not be told which type of blood replacement you have received but you can find this out when the study has ended (more information is provided on page 7). In accordance with standard practice, women who receive donor blood will only be given a blood transfusion if there is enough blood loss during the operation. Women allocated to ICS blood will be given ICS blood return, even if there is only a little blood loss during the operation.

What if I am to receive chemotherapy before my surgery?

Your surgeon / doctor at the hospital will inform you if you require chemotherapy prior to your surgery and will provide you with the information you need. This is called neo-adjuvant chemotherapy. You can still take part in the study if you need chemotherapy first.

What happens if I do not require blood replacement?.

Women undergoing surgery for ovarian cancer (or suspected cancer) do not always require blood replacement, so some women taking part in this study will not receive blood of any sort. If you are one of these women, you are still very important to the study and we would still like to collect the same information from you.

Blood replacement <u>after</u> your operation

Sometimes it is usual for some patients to require blood replacement after their operation, either in the recovery unit or on the ward. If you need blood after surgery, this will be a donor blood transfusion regardless of whether you were in the group that received donor blood or your own ICS blood during your operation. This is because the ICS machine cannot be used on the hospital ward. If you require a donor transfusion after surgery your participation in the study will still be valid and it is important that your information is still l included in the study analysis.

What happens after I've had my operation?

Participation in this study will not interfere with your usual care and recovery from surgery and should not delay your discharge home. If you take part in this study, the research nurse will record some details about your operation, recovery and whether or not you needed blood replacement. Any routine hospital follow-up visits will continue as usual.

Approximately **30 days** after your operation the research nurse will telephone you to ask about your general health and wellbeing since you were discharged from hospital. **Six weeks** after your operation you will be sent a questionnaire booklet to complete - the length of the booklet depends upon your final diagnosis. Your completed questionnaire booklet should be returned in the pre-paid envelope provided.

People who are recruited in the early stages of the study will be sent repeat questionnaire booklets, at three month intervals, as time allows (see flowchart on page 5). At these time points we will also ask you about any contacts you may have had with your hospital, GP, district nurse or other services since discharge from hospital. The questionnaire booklet should take no longer than 30 minutes to complete in total.

Do I have to take part?

No. Taking part in this study is entirely voluntary and it is up to you to decide whether or not to take part. If you do decide to take part you will be asked to sign a consent form, but you are still free to withdraw at any time in the future and without giving a reason. You will be given a copy of this information sheet and a copy of your signed consent form to keep. If you decide not to take part, or you withdraw from the study at any point, your medical care will not be affected in any way.

What will happen to me if I do NOT take part in the study?

If you decide this study is not for you at this time, you will be given the usual care and treatment offered at your hospital. If you need blood replacement during your operation, you may be offered ICS blood return as part of usual care (depending on the extent of your surgery

and the expected blood loss) even if you do not take part in the study. Usual care varies between hospitals because there is currently no evidence to show that receiving your own blood (ICS blood) is better or worse than donor blood. Currently ICS blood reinfusion is offered for ovarian cancer surgery in two of the hospitals participating in this study (Truro and Plymouth) but is not routinely offered in Gateshead or Leicester.

Study flowchart

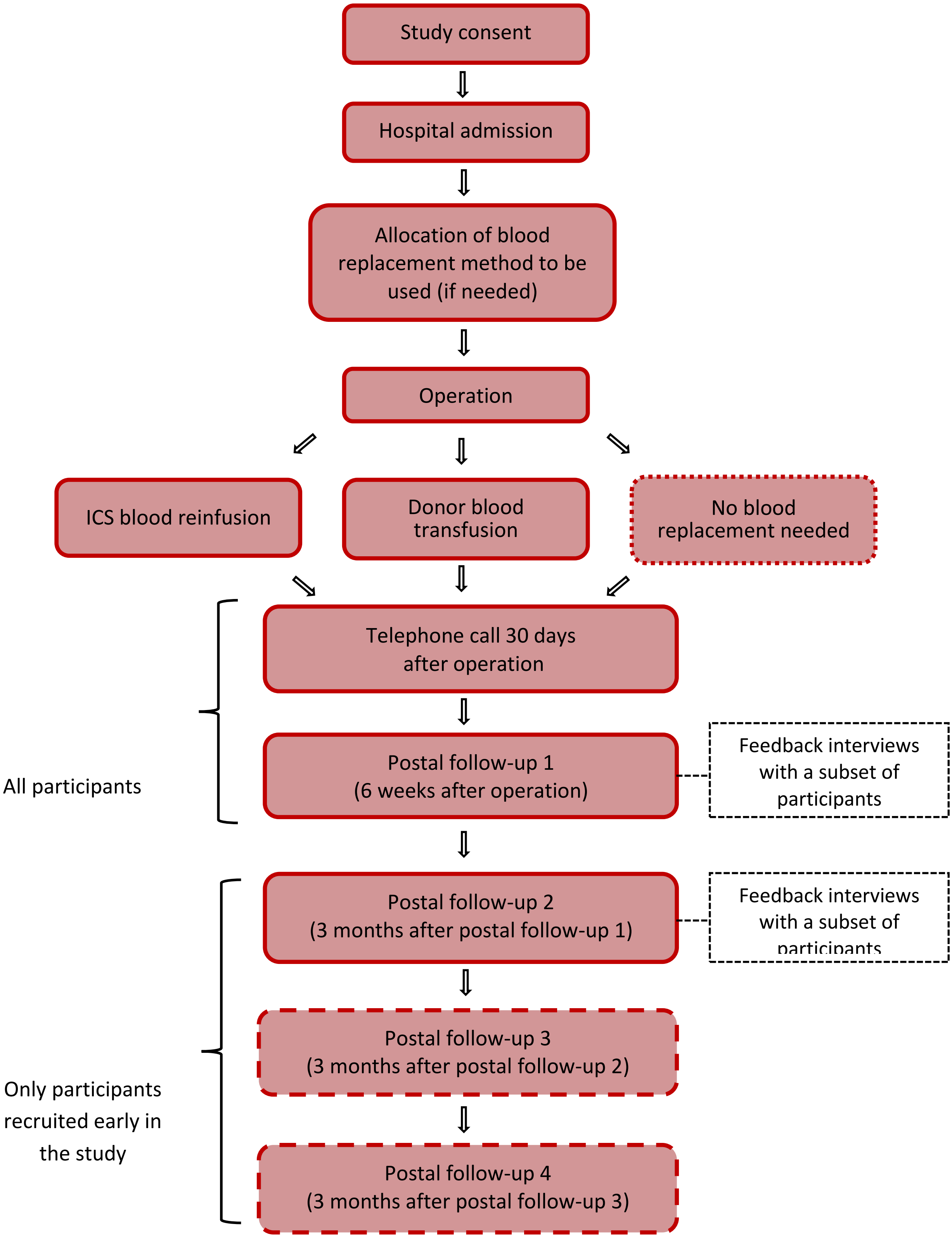

What are the possible benefits of taking part?

You may or may not benefit directly from this study but by taking part you will be contributing to a study which could potentially bring future benefit to women with ovarian cancer around the world. We don't know whether one method of blood replacement compared with the other improves recovery after surgery for ovarian cancer, but we hope that this study will help to answer that question.

What are the possible disadvantages and risks of taking part?

If you agree to take part in this study you will be asked to complete some questionnaire booklets, as described on page 4. These will take 15-30 minutes on each occasion and you will be asked to complete these two times (and up to a maximum of five times) over several months.

You may or may not receive blood replacement if you take part in this study. The anaesthetist and surgeon will decide whether you require additional blood during your operation as part of your usual care. If you need blood replacement during surgery, you may be given a donor blood transfusion or you may be given ICS blood return.

With any donor blood transfusion there is a possibility of side effects, including an increased risk of wound or other infections, lung and kidney problems, and a risk of receiving the wrong blood type in error. Such events and adverse transfusion reactions are rare. Donor blood transfusion has been used widely for many years and is considered a safe way of delivering blood to patients.

Intraoperative cell salvage (ICS) has been used in cancer operations, including ovarian cancer. Its use has been limited because of the theoretical risk (i.e. based on theory rather than experience) of reintroducing cancer cells into the bloodstream. However, the risk of cancer cells entering the bloodstream is low as far as current evidence shows, because a special filter is used which can remove any active cancer cells from the returned blood.

It is possible that the ICS technique may cause a temporary lowering of blood pressure but this is monitored continuously during the operation and any problem can be quickly corrected. There are no other documented problems with using ICS known to date.

What happens when the research study stops?

Once your participation in the study has ended, your usual care will continue as before. When every woman has completed their involvement in the study, we will prepare the study results (which normally takes several months) which will be available to participants.

If you would like to know whether you received a donor blood transfusion or ICS blood return, your research team will be able to tell you once everyone has completed the study. This is likely to be in the summer of 2018. The study results may be presented at national and international conferences and published in medical journals but you will not be identified in any information included in any presentation or publication.

General information about this study

What if relevant new information becomes available?

A special committee will be set up to look at all the information collected during the course of the TIC TOC study and will ensure that any study-related issues of concern are investigated. If the study is stopped for any reason, you will be told why. If any new information about ICS blood return becomes available which might affect your participation in the study, you will be informed.

What happens if I don't want to carry on with the study?

You are free to withdraw from the study at any time, without giving any reason, and without your medical care or legal rights being affected. If you want to withdraw from the study before you have your surgery, you must do this before you are given any anaesthetic. If you decide to withdraw from the study at any stage, we may still use information collected about you unless you ask us not to.

What if there is a problem?

Complaints: If you have a concern about any aspect of this study, please speak to someone in your research team who will do their best to answer your questions. If you remain unhappy and wish to complain formally, you can do this through your local NHS complaints procedure. The NHS has a Patient Advice and Liaison Service (PALS) for information and support, which can be found at your local hospital <Enter local PALS contact details>. You can also contact the department responsible for overseeing the study: Research, Development and

Innovations Manager, Knowledge Spa, Royal Cornwall Hospitals NHS Trust, Truro TR1 3HD. Tel: 01872 246424.

Harm: We don't expect any harm to come to you as a result of participating in this study. If you are harmed and this is due to someone's negligence, then you may have grounds for legal action for compensation against your hospital's Trust but you may have to pay your legal costs.

There are no special compensation arrangements in place. The normal NHS complaints mechanisms will still be available to you; your PALS service will be able to advise you.

Private insurance policies: Please note that it is your responsibility to check if taking part in this study affects the terms and conditions of any private insurance policies that you hold.

Will the information collected during the study be kept confidential?

All information collected about you whilst taking part in this study will be kept strictly confidential and will be collected and stored for five years after the study is complete, in accordance with the Data Protection Act (1998). You will be given a unique study number which your study information will be labelled with, along with your initials, so that you cannot be identified (known as pseudonymised or de-identified data). This study information will be stored and analysed at Plymouth University. Only members of the research team and the Peninsula Clinical Trials Unit (PenCTU) at Plymouth University will have direct access to the study information. Paper-based information will be stored in locked filing cabinets within a locked office in the PenCTU. Information kept on computers will be stored securely on a system maintained by Plymouth University. Copies of the study information will be held securely at your local hospital but will not contain any details that could identify you.

Authorised people from your NHS Trust, the PenCTU and study organisers may need to review your medical records to check that the study is being carried out correctly. All will have a duty of confidentiality to you as a research participant. As part of the consent process, you will be asked to consent to your contact details (name, address, telephone number) being provided to the PenCTU and the TIC TOC researcher based at Royal Cornwall Hospitals NHS Trust, to enable collection of some information by post. At the PenCTU, these details will be stored separately from the de-identified study information also held.

Will the study information help with other research projects?

It is important that good quality research data can be shared with others in order to advance clinical research and to benefit patients in the future. After the end of the study, de-identified information collected during the study will be made available to other researchers under an appropriate data sharing agreement, but it will not be possible to identify you personally from any information shared.

This is a feasibility study, so the aim is to test the processes required for a large study. This study will provide us with the necessary information to help us to learn what to consider when designing a main study in the future. Ultimately, the main study will assess whether ICS or donor blood transfusion is associated with better outcomes for patients having ovarian cancer surgery.

Involvement of your General Practitioner/ Family Doctor (GP)

Your general practitioner will be informed of your participation in this study.

Who is organising and funding the study?

The study is being led by Miss Khadra Galaal, Consultant Gynaecological Oncologist at the Royal Cornwall Hospitals NHS Trust (RCHT). The study is funded by the National Institute for Health Research (NIHR) Research for Patient Benefit grant scheme Ref: PB-PG-1014-35005. The study will be managed by the Peninsula Clinical Trials Unit at Plymouth University and sponsored (overseen) by RCHT.

Who has reviewed the study?

All NHS research is looked at by an independent panel (Research Ethics Committee). This study has been reviewed and been given a favourable opinion by the <Enter name> Research Ethics Committee.

Thank you for taking the time to read this information sheet and for considering taking part in the TIC TOC study.

TIC TOC

TO BE PRINTED ON RELEVANT NHS TRUST HEADED PAPER

Appendix 2: Informed consent form

PARTICIPANT CONSENT FORM

A randomised, controlled feasibility trial of intraoperative cell salvage versus donor blood transfusion in ovarian cancer surgery

Principal Investigator: <Insert PI’s name> **Participant Study Number:** ☐☐☐☐

Please initial each box

1. I confirm that I have read and understood the information sheet (version 2.0, dated 09 October 2017) for the above study. I have had the opportunity to consider the information and ask questions and I have had my questions answered satisfactorily. ☐
2. I understand that my participation in this study is voluntary and that I am free to withdraw at any time without giving any reason and without my medical care or legal rights being affected. ☐
3. I agree that my name, address and telephone number can be given to and stored by the Peninsula Clinical Trials Unit at Plymouth University to enable collection of study information by post. ☐
4. I understand that relevant sections of any of my medical notes and information collected during the study may be looked at by responsible individuals from my local NHS Trust, the Peninsula Clinical Trials Unit and the regulatory authorities, where it is relevant to my taking part in this research. I give permission for these individuals to have access to my records. ☐
5. I understand that an anonymised copy of this consent form will be sent to the Peninsula Clinical Trials Unit to confirm my agreement to participate. ☐
6. I understand that the information collected about me may be shared anonymously with other researchers to support future research studies. I cannot be personally identified from this. ☐
7. I agree to take part in the TIC TOC study. ☐

______________________	______________________	______________________
Print Name (Participant)	*Date*	*Signature*
______________________	______________________	______________________
Print Name (Researcher taking consent)	*Date*	*Signature*

Appendix 3: FIGO Ovarian Cancer Staging

Effective 1 January 2014

**STAGE I: Tumour confined to ovaries**	
**IA**	Tumour limited to one ovary, capsule intact, no tumour on surface, negative washings
**IB**	Tumour involves both ovaries, otherwise like IA
**IC**	Tumour limited to one or both ovaries
IC1	Surgical spill
IC2	Capsule rupture before surgery or tumour on ovarian surface
IC3	Malignant cells in the ascites or peritoneal washings

**STAGE II: Tumour involves one or both ovaries with pelvic extension (below the pelvic brim) or primary peritoneal cancer**	
**IIA**	Extension and/or implant on uterus and/or Fallopian tubes
**IIB**	Extension to other pelvic intraperitoneal tissues

**STAGE III: Tumour involves 1 or both ovaries with cytologically or histologically confirmed spread to the peritoneum outside the pelvis and/or metastasis to the retroperitoneal lymph nodes**		
**IIIA**	Positive retroperitoneal lymph nodes and/or microscopic metastasis beyond the pelvis	
IIIA1	Positive retroperitoneal lymph nodes only	
	IIIA1 (i)	Metastasis ≤ 10mm
	IIIA1 (ii)	Metastasis > 10mm
IIIA2	Microscopic, extrapelvic (above the brim) peritoneal involvement ± positive retroperitoneal lymph nodes	
IIIB	Macroscopic, extrapelvic, peritoneal metastasis ≤ 2cm ± positive retroperitoneal lymph nodes. Includes extension to capsule of liver/spleen	
IIIC	Macroscopic, extrapelvic, peritoneal metastasis > 2cm ± positive retroperitoneal lymph nodes. Includes extension to capsule of liver/spleen	

**STAGE IV: Distant metastasis excluding peritoneal metastasis**	
IVA	Pleural effusion with positive cytology
IVB	Hepatic and/or splenic parenchymal metastasis, metastasis to extra-abdominal organs (including inguinal lymph nodes and lymph nodes outside of the abdominal cavity)

Other major recommendations are as follows:

- Histologic type including grading should be designated at staging
- Primary site (ovary, Fallopian tube or peritoneum) should be designated where possible
- Tumours that may otherwise qualify for stage I but involved with dense adhesions justify upgrading to stage II if tumour cells are histologically proven to be present in the adhesions.

Appendix 4: Definition of surgical site infection

For the purposes of this study, surgical site infection (48, 49) is defined as an infection that:-

i) occurs within 30 days after the operation and

ii) appears to be related to the operation and

iii) involves deep soft tissues (e.g. fascial and muscle layers) of the incision and at least one of the following:-

a) Purulent drainage from the deep incision but not from the organ/space component of the surgical site

b) A deep incision spontaneously dehisces or is deliberately opened by a surgeon when the patient has at least one of the following signs or symptoms: fever (> 38 C), localized pain, or tenderness, unless site is culture-negative.

c) An abscess or other evidence of infection involving the deep incision is found on direct examination, during reoperation, or by histopathologic or radiologic examination.

d) Diagnosis of a deep incision SSI by a surgeon or attending physician

Appendix 5: Topic guide for participant interviews

First qualitative interview (6 weeks)

Topic	Questions	Prompts
**Opening question**	How are you feeling after your operation? Tell me a bit about yourself?	Role in life – past or present employment<br><br>Family<br><br>Be sensitive and understanding
**Recruitment**	How were you approached to take part in the TIC TOC study?<br><br>What did you think about the way the study was introduced?<br><br>What did you understand about the study?<br><br>What questions did you have? Did you receive answers you understood?	Which member of staff, how approached(surgeon, specialist nurse)
**Specific understanding**	What did you understand about reintroducing your own blood?<br><br>What did you understand by donor blood transfusion?	Which method did you think was safest?
**Involvement of family and friends**	Did you ask anyone else for their opinions?<br><br>If yes, who were they?<br><br>What was their opinion?	Explore any negative responses from family and friends<br><br>Explore any positive responses from family and friends
**Decision process**	What things did you think about when deciding if you were going to take part?	Barriers<br><br>Factors that stopped the woman taking part (fear, overwhelmed by potential cancer diagnosis, chance would get cell salvage anyway (some

		sites), lack of understanding, unable to read research literature)<br><br>Facilitators<br><br>Factors that encouraged her to take part (trust of surgeon, research staff, feeling obligated, fear, distrust of donated blood or salvaged blood)
**Research processes**	When you came to the first clinic to see your consultant, how were you treated in the research part of your appointment?<br><br>Tell me what you felt about the specialist nurse asking you if you wanted to take part in the TIC TOC study?<br><br>What did you think about the timing of being recruited to the study?<br><br>What did you think about the questionnaires?	Check woman's talk is about the research.<br><br>Woman may want to talk about their cancer experience – allow it.<br><br>Baseline questionnaires only
**Allocation**	Which group do you think you were allocated?<br><br>Why?	Do not say which
**Information about next appointments**	As part of your normal care, you will be followed up by your consultant or his/her team. As part of the research study you will receive some further postal questionnaires.	

	Can I contact you again in about 6 months to see what you think about the postal follow-up?	

Second qualitative interview (three months after first, by telephone)

**Topic**	**Questions**	**Prompts**
**Opening question**	Since we last spoke, how have you been getting on?<br><br>I have a few questions to ask you about your experience of taking part in the TIC TOC study.	May not be feeling well.<br><br>May be on chemotherapy treatment.<br><br>Be sensitive and understanding
**Research process:**<br><br>**follow-up questionnaires**	Where did you complete your questionnaires?<br><br>Did you have help to complete the questionnaires?<br><br>What did you like about the telephone/postal follow up?<br><br>What didn't you like about the telephone/postal follow up?<br><br>Was there anything that could be improved?<br><br>Did you know who to contact if you did not wish to keep taking part?	Did the woman know how to make a complaint?

	What did you think about the questionnaires asking you what health services you had used?	(probe questionnaires by telephone)<br><br>Check view about the number of questionnaires and clarity of questions<br><br>Check for questionnaire burden
**Allocation**	Which group do you think you were allocated?<br><br>Why?	**Do not say which.**<br><br>**The woman will receive notification about the allocation at the end of the study.**
	Thank you for taking part in the research study that will help inform a larger study.<br><br>Wish well for the future.	
