# Supplementary material for: Trial of intraoperative cell salvage versus transfusion in ovarian cancer (TIC TOC): protocol for a randomised controlled feasibility study
Source: BMJ Open. 2018 Nov 1;8(11):e024108. doi: 10.1136/bmjopen-2018-024108 (PMC6224724; doi:10.1136/bmjopen-2018-024108)
Supplement: Author's manuscript [file bmjopen-2018-024108.draft_revisions.pdf]

BMJ Open

BMJ Open is committed to open peer review. As part of this commitment we make the peer review history of every article we publish publicly available.

When an article is published we post the peer reviewers' comments and the authors' responses online. We also post the versions of the paper that were used during peer review. These are the versions that the peer review comments apply to.

The versions of the paper that follow are the versions that were submitted during the peer review process. They are not the versions of record or the final published versions. They should not be cited or distributed as the published version of this manuscript.

BMJ Open is an open access journal and the full, final, typeset and author-corrected version of record of the manuscript is available on our site with no access controls, subscription charges or pay-per-view fees (http://bmjopen.bmj.com).

If you have any questions on BMJ Open's open peer review process please email info.bmjopen@bmj.com

BMJ Open

Trial of intraoperative cell salvage versus transfusion in ovarian cancer (TIC TOC): protocol for a randomised controlled feasibility study

Journal:	*BMJ Open*
Manuscript ID	bmjopen-2018-024108
Article Type:	Protocol
Date Submitted by the Author:	09-May-2018
Complete List of Authors:	Galaal, Khadra; Royal Cornwall Hospitals NHS Trust, Gynaeoncology<br>Lopes, Alberto; Royal Cornwall Hospitals NHS Trust<br>Pritchard, Colin; Royal Cornwall Hospitals NHS Trust, Research, Development and Innovation<br>Barton, Andy; NIHR South West Research Design Service<br>Wingham, Jennifer; University of Exeter Medical School,<br>Marques, Elsa; University of Bristol, School of Social and Community Medicine<br>Faulds, John; Royal Cornwall Hospitals NHS Trust<br>Palmer, Joanne; Hull York Medical School, Vascular Research Unit<br>Vickery, Patricia; Plymouth University, Peninsula Clinical Trials Unit<br>Ralph, Catherine; Royal Cornwall Hospitals NHS Trust, Anaesthetics<br>Ferreira, Nicole ; Royal Cornwall Hospitals NHS Trust<br>Ewings, Paul; Research Design Service, Research Office
Keywords:	intraoperative cell salvage, donor blood transfusion, cytoreductive surgery, ovarian cancer, feasibility trial, quality of life

SCHOLARONE™
Manuscripts

Trial of intraoperative cell salvage versus transfusion in ovarian cancer (TIC TOC): protocol for a randomised controlled feasibility study

Khadra Galaal[1], Alberto Lopes[2], Colin Pritchard[3], Andrew Barton[4], Jennifer Wingham[2], Elsa Marques[5], John Faulds[6], Joanne Palmer[3], Jane Vickery[7], Catherine Ralph[9], Nicole Ferreira[6], Paul Ewings[4].

1. Gynaecology, Royal Cornwall Hospitals NHS Trust, Truro, Cornwall, UK
2. University of Exeter, Devon, UK
3. Research, Development and Innovation, Royal Cornwall Hospitals NHS Trust, Truro, Cornwall, UK
4. NIHR Research Design Service (South West)
5. Bristol Medical School, University of Bristol, Bristol, UK
6. Royal Cornwall Hospitals NHS Trust, Truro, Cornwall, UK
7. Peninsula Clinical Trials Unit, Plymouth University, Plymouth, UK
8. Department of Anaesthesia, Royal Cornwall Hospitals NHS Trust, Truro, Cornwall, UK.

Corresponding Author

Miss Khadra Galaal FRCOG

Consultant Gynaecological Oncologist

Royal Cornwall Hospital, Truro, UK

Tel: 01872 252731

Email: k.galaal@nhs.net

Key words: intraoperative cell salvage, donor blood transfusion, cytoreductive surgery, ovarian cancer, feasibility trial, economic evaluation, quality of life.

For peer review only

ABSTRACT

Introduction: Ovarian cancer is the leading cause of death from gynaecological cancer, with more than 7,000 new cases registered in the UK in 2014. In patients suitable for surgery, NICE guidance for treatment recommends surgical resection of all macroscopic tumour, followed by chemotherapy. The surgical procedure can be extensive and associated with substantial blood loss which is conventionally replaced with a donor blood transfusion. Whilst often necessary and life-saving, the use of donor blood is associated with increased risks of complications and adverse surgical outcomes. Intraoperative cell salvage (ICS) is a blood conservation strategy in which red cells collected from blood lost during surgery are returned to the patient thus minimising the use of donor blood. This is the protocol for a feasibility randomised controlled trial with an embedded qualitative study and feasibility economic evaluation. If feasible, a later definitive trial will test the effectiveness and cost-effectiveness of ICS reinfusion versus donor blood transfusion in ovarian cancer surgery.

Methods and analysis: Sixty adult females scheduled for primary or interval ovarian cancer surgery at participating UK NHS Trusts will be recruited and individually randomised in a 1:1 ratio to receive intraoperative cell salvage reinfusion or donor blood (as required) during surgery. Participants will be followed up by telephone at 30 days post-operatively for adverse events monitoring and by postal questionnaire at six weeks and three monthly thereafter, to capture quality of life and resource use data. Qualitative interviews will capture participants' and clinicians' experiences of the study.

Ethics and dissemination: This study has been granted ethical approval by the South West - Exeter Research Ethics Committee (ref: 16/SW/0256). Results will be disseminated via peer-reviewed publications and will inform the design of a larger trial.

Trial registration number: ISRCTN19517317

For peer review only

For peer review only - http://bmjopen.bmj.com/site/about/guidelines.xhtml

Strengths and limitations of this study

- This is the first study to use intraoperative cell salvage in cytoreductive surgery for ovarian cancer
- The study explores the feasibility and informs the design of a larger randomised controlled trial. Quantitative, qualitative and feasibility economic components are included
- Limitations are;
    - The effect of transfusion and cell salvage on immune response to surgery is not assessed
    - This feasibility study will not provide information on the long-term outcomes of using either cell salvage or transfusion.

For peer review only

INTRODUCTION

Background

Ovarian cancer is the leading cause of death from gynaecological cancer in the UK (age-standardised mortality rate 9.1 per 100,000 2008-2010) (1). Although survival rates have improved in recent decades, there are still more deaths from ovarian cancer than all other gynaecological cancers combined (2). The mainstays of treatment for advanced ovarian cancer are surgical cytoreduction and platinum-based chemotherapy. As operative success and survival is largely determined by residual disease (3). Surgery is often extensive with substantial intraoperative blood loss, about 53% of patients lose more than 1.5 litres during their first surgery (4). Blood lost during surgery is conventionally replaced using donor blood transfusion with the incidence of transfusion ranging from 35% to 77% (5, 6). Perioperative donor blood transfusion is associated with increased risks of complications and adverse surgical outcomes including mortality, wound infection, pulmonary and renal complications, systemic sepsis and prolonged hospital stay (7). In 2012 there were 12.3 serious adverse incidents per 10,000 transfused components reported by the Serious Hazards of Transfusion (SHOT) group (8). SHOT is an independent, professionally-led scheme, involved in collecting and analysing anonymised information on adverse events and reactions in blood transfusion from all healthcare organisations in the U.K. Where risks and problems are identified, they produce recommendations to improve patient safety. One suggested explanation for adverse reactions is a general transient depression of the immune system following transfusion with blood products, transfusion-induced immunomodulation (TRIM) (9, 10).

Intraoperative cell salvage (ICS) or autologous blood transfusion is the practice of recovering red cells from blood lost in the operative field and returning them to the patient (11). This process involves the separation, centrifugation, washing and filtration of heparinised red blood cells, before reinfusion into the patient. ICS eliminates or reduces the need for donor blood transfusion and its associated risks, making it an alternative where major blood loss is anticipated (12). ICS can be available in theatre at modest expense and reduces dependence on the limited pool of banked blood.

For peer review only - http://bmjopen.bmj.com/site/about/guidelines.xhtml

Studies comparing cell salvage with allogeneic blood transfusion have demonstrated increased mean erythrocyte (red blood cells) viability as high as 88% with cell salvage (13-15). ICS has been used successfully in surgical specialties (16) including cardiothoracic, vascular, orthopaedic and hepatobiliary (17-20). In addition, intraoperative cell salvage is associated with low rate of patient-related adverse events (21). ICS was initially contraindicated in cancer because of the theoretical risk of reintroducing malignant tumour cells into patients' bloodstreams (22, 23). However, such concerns appear to be unfounded(24). The *in vitro,* leucocyte depletion filters are highly efficient at removing malignant cells with removal rates of between 80 and 100% (25, 26). In patients undergoing surgery for gynaecological malignancy, leucocyte depletion filters effectively eliminate viable nucleated malignant cells from the returned blood (27, 28). Far from compromising outcomes, ICS is associated with improved outcomes in cervical (29, 30) and oesophageal cancers (24).

Interestingly, patients with primary metastatic cancer are known to have circulating tumour cells in the blood (31). Furthermore, operative manipulation of tumours during surgery leads to peripheral blood concentrations of malignant cells many times higher than could be attained with cell salvage (32). The presence of circulating tumour cells is prevalent in cancer patients with approximately one circulating tumour cell (CTC) per 105 to 107 mononuclear cells found in the peripheral blood of metastatic cancer patients (33).

Rationale

There is a paucity of studies in ICS, making it difficult for patients, clinicians and NHS managers to make decisions about this technology (34). ICS has been used in ovarian cancer patients in one of the participating sites with encouraging results, but a randomised controlled trial (RCT) is required for robust determination of effectiveness. The aim of a definitive trial would be to assess the clinical and cost-effectiveness of intraoperative cell salvage for women undergoing cytoreductive surgery for ovarian cancer, compared with usual practice of transfusing only allogeneic blood as required.

For peer review only

Aim and objectives

The aim of the study is to determine whether a definitive randomised controlled trial is feasible and, if so, how best to deliver it. The objectives of the study are to:

- Estimate the likely recruitment rate for the larger trial
- Estimate the likely completeness of resource use and outcome data
- Explore the practical logistics of undertaking randomisation in theatres
- Assess success of blinding of allocation for participants and outcome assessors
- Design data collection tools to collect resource use data from participants, hospital medical records and hospital staff
- Inform the trial design and confirm the resources required to run a larger definitive trial
- Explore the barriers and facilitators for women when deciding whether or not to participate
- Explore women's perceptions of:
    - The intervention, the information given and advantages/disadvantages of participation so that information can be optimised for the larger trial
    - Other trial aspects, e.g. regarding collection of outcome measures and completing resource use questionnaires.
- Identify factors influencing surgeons' decisions about whether or not to participate in the study.

METHODS

Trial design

This is a protocol for a randomised, controlled, multi-centre feasibility study in women undergoing cytoreductive surgery for ovarian cancer. Sixty participants will be individually randomised in a 1:1 ratio to intraoperative cell salvage (re-infusion of their own blood) or donor blood transfusion during surgery. Participants and outcome assessors will be blinded to the intervention. All participants will be followed up by telephone for adverse events reporting at 30 days post-

For peer review only

operatively, by post six weeks post-operatively and three monthly thereafter as time allows. A schematic diagram of the trial is given in Figure 1. The feasibility study includes an embedded qualitative component to assess participants' (patients and clinicians) perceptions of their experience in preparation for the later trial. It will also involve an assessment of the feasibility of collecting resource use and other economic data for a future economic evaluation.

Study setting

The study will take place at the Royal Cornwall Hospitals NHS Trust, Plymouth Hospitals NHS Trust, Gateshead Health NHS Foundation Trust and University Hospitals of Leicester NHS Trust. All sites have existing personnel experienced in the management of intraoperative cell salvage and reinfusion.

Participants and recruitment

Participants will be recruited from patients scheduled to undergo surgery for ovarian cancer at the participating hospitals. Potential participants will usually be identified from those patients attending the gynaecological oncology out-patient clinic having been referred by their GP under the two week wait cancer pathway. Some patients will be scheduled for primary surgery and are suitable for immediate recruitment to the study. Others will undergo neo-adjuvant chemotherapy prior to interval debulking surgery and may be recruited to the study at a later date, following chemotherapy. Written informed consent will be obtained by an appropriately trained member of the research team in line with ICH Good Clinical Practice (GCP) guidelines. As part of the consent process, patients will be reminded that they are free to withdraw from the study at any time without giving a reason and without affecting their future treatment.

Inclusion criteria

Potential participants must satisfy the following criteria to be enrolled in the study:

• 18 years old or over

For peer review only

• Suspected or confirmed ovarian cancer (newly diagnosed) requiring cytoreductive surgery, whether primary or interval (following chemotherapy)

• CT scan evidence (with or without clinical evidence) compatible with FIGO stage III/IV ovarian cancer/primary peritoneal cancer at presentation (35) (Appendix 1)

• Eastern Cooperative Oncology Group (ECOG) Performance Status 0-1 (36)

• Willing to participate and able to give written informed consent

Exclusion criteria

Potential participants meeting any of the following criteria will be excluded from study participation:

• Diagnosis of concurrent malignancy

• Pregnant

• Haemoglobinopathies (e.g. sickle cell, thalassaemia)

• Unwilling to accept donor blood (e.g. on religious grounds)

Randomisation

Randomisation will be undertaken after written consent has been obtained, but as close to the start of surgery as possible; usually this will be on the morning of the operation day but if this is not possible for practical reasons, it may be performed earlier. Randomisation will be achieved by means of a web-based system created by the UK Clinical Research Collaboration (UKCRC) registered Peninsula Clinical Trials Unit (CTU) in conjunction with the trial statistician, using random permuted blocks of varying size. Participants will be allocated to receive ICS reinfusion or donor blood transfusion in a 1:1 ratio, stratified by study site. To prevent any unnecessary delays in the operating theatre, cell salvage equipment will be set up in advance for all study participants, before confirmation of treatment allocation.

Trial interventions

For peer review only

Participants will be allocated to receive either donor blood transfusion or ICS reinfusion intraoperatively, in accordance with specified transfusion protocols. Donor blood will only be given (in standard volumes) when deemed necessary (e.g. after substantial blood loss and/or drop in haemoglobin) whereas ICS blood will be returned even if only small quantities are lost. Some participants may not require any intraoperative transfusion and some (in either arm of the trial) may require donor blood transfusion post-operatively.

Intraoperative cell salvage

All sites will follow a common ICS protocol and relevant site staff will undergo study-specific training prior to the study start. The make and model of ICS machine used in clinical practice varies across NHS Trusts and will not be standardised for this feasibility study. Collected blood will be processed via the ICS machine being used before being re-infused via a leucodepletion filter. Relevant data from a local intraoperative cell salvage audit form will be transcribed into the study-specific Case Report Form (CRF), including the amounts of salvaged blood processed and reinfused.

Donor transfusion

Participants allocated to donor transfusion will be considered for transfusion during surgery in accordance with clinical judgement, guided by local hospital policy. The factors triggering transfusion (e.g. excessive blood loss, hypotension, reduced Hb) will be documented in the CRF along with the amount and type of blood and blood products transfused.

Donor transfusion in ICS arm

Participants allocated to the ICS arm who need donor transfusion can be given donor blood at any time, during or after surgery, for the duration of their hospital stay. The factors triggering intraoperative donor transfusion in the ICS group will be documented in the CRF as well as the amount and type of any blood and blood products transfused.

Blinding

For peer review only

For peer review only - http://bmjopen.bmj.com/site/about/guidelines.xhtml

Surgeons, other theatre staff and the person recording details of intra-operative blood transfusion or reinfusion cannot be blinded in this study. The research nurse responsible for recording post-operative outcomes will aim to remain blinded to treatment allocation. Participants in either arm of the study may have some form of blood replacement in progress immediately post-surgery; it is unlikely that participants will be able to distinguish between the two types and either group may require donor blood for clinical reasons.

Feasibility outcomes

The outcomes for this study are the feasibility and acceptability of the study and study procedures in relation to recruitment, randomisation, intervention, blinding, participant retention and data completion. Both quantitative and qualitative methods will be used. Recruitment rate will be measured as the proportion of eligible patients who are subsequently enrolled and the number of patients recruited per site per month. The number of patients screened, number/percent of patients approached, number/percent of patients excluded after screening/approach and the number/percent of patients providing consent will be assessed. Reasons for declining participation will be sought where possible, and the appropriateness and practicalities of the chosen eligibility criteria will be explored. The number/percent of women enrolled prior to initial surgery compared to following neo-adjuvant chemotherapy will be assessed. The timing of randomisation in relation to operation start will be recorded to assess the practicalities of randomising as late as possible, in particular what proportion are randomised on the day of surgery itself.

Use of ICS blood and donor blood will be recorded for both arms, partly to assess intervention fidelity but also to obtain an estimate of the proportion of people in the control arm that actually require donor blood. Reasons for non-use of ICS blood and/or use of donor blood in the ICS arm will be recorded.

Since the intervention takes place in the operating theatre it is unlikely that any participant will withdraw from intervention following randomisation. Attrition will be assessed by examining the

For peer review only - http://bmjopen.bmj.com/site/about/guidelines.xhtml

number of participants lost to follow-up at any subsequent point in the study period. Reasons for discontinuation of follow-up will be sought from participants.

The success of blinding of allocation for participants and outcome assessors will be assessed by asking both the participant and research nurse to guess the allocation (including "unsure") at the 30 day post-operative follow-up and comparing the responses with the actual allocation.

Clinical outcomes

In the later, definitive trial, our primary outcome is likely to be either mortality or cancer recurrence, both of which are unlikely to occur in the time available in this feasibility study. Therefore, whilst readily accessible, these data will not be collected here. Other measures proposed for the later trial will be collected in this feasibility study at baseline and peri-operatively, with follow-up at 30 days and 6 weeks post-operatively. Participants recruited at an early stage of the study will also be followed up at 4.5, 7.5 and 10.5 months post-operatively as time allows (Figure 1). Clinical outcomes include:

- Inadvertent visceral injury (bladder, bowel, ureters, blood vessels, nerve)
- Return to theatre within 48 hours
- Surgical site infection (Appendix 2) within 30 days
- Thromboembolic complications (DVT, PE) within 30 days
- Number and nature of adverse events
- Amount of donor blood given (total and ≤24 hours post-surgery)
- Length of hospital stay
- Resource use
- Generic quality of life (QOL) measure: EQ-5D-5L
- Cancer-specific QOL measure: EORTC QLQ-C30 (Version 3.0) (confirmed cancer only)
- Ovarian cancer QOL measure: EORTC QLQ-OV28 (confirmed cancer only)

Data management

For peer review only

For peer review only - http://bmjopen.bmj.com/site/about/guidelines.xhtml

Each participant will be allocated a unique trial number on consenting to the study and will be identified in all study-related documentation by her trial number and initials. A record of names and addresses linked to participants' trial numbers will be maintained by the research nurse at each site for administrative purposes, and stored securely.

Data collection

Data collected by the research team (Table 1) up to 30 days post-operatively will be recorded on study specific data collection forms (CRFs), usually by a research nurse. All data not routinely captured during the hospital admission but recorded straight into the CRF will be classified as source data. Participant self-completion questionnaires at baseline will be completed during a face-to-face meeting with a research nurse, following written informed consent. The research nurse will return completed CRFs and baseline questionnaires to the CTU. Subsequent self-completion questionnaires (6 weeks post-operatively and three monthly thereafter as time allows) will be mailed to participants directly from the CTU and returned by participants to the CTU in a pre-paid envelope provided. In the event of non-return of a questionnaire, a reminder will be sent from the CTU in the first instance. If there is no response from the two mailings, the CTU will inform the local research nurse who will telephone the participant in order to encourage compliance with follow-up.

For peer review only

Table 1: Trial schedule

	Pre-operative		OPERATION and peri-operative data collection	Post-operative follow-up				
	Screen	Baseline		1	2	3†	4†	5†
				30 days post-op	6 weeks post-op	3 months after follow-up 2	6 months after follow-up 2	9 months after follow-up 2
Screen/eligibility	x							
Consent		x						
Demographics & history		x						
Randomisation		x						

EORTC QLQ-C30*		x			x	x	x	x
EORTC QLQ-OV28*		x			x	x	x	x
EQ-5D-5L		x			x	x	x	x
Adverse events				x				
Resource use questionnaire		x			x	x		
Qualitative interviews					x	x		

Statistical considerations

Sample size for a feasibility study is necessarily a compromise between the twin assets of precision and efficiency. For any binary "outcome" our target sample size of 60 will result in a 95% confidence interval of no greater than about +/-12 percentage points, while in a single arm the target of 30 will have a CI of no more than +/- 17 percentage points.

Data analysis will enable the feasibility outcomes to be addressed in order to inform a decision about proceeding to a definitive trial. Data will be presented in accordance with the extension to the ConSORT statement for pilot and feasibility studies. They will detail the numbers of patients that were approached, the number that were eligible and the number providing consent. Likewise, compliance rates at all stages will be presented, including the numbers of questionnaires completed at each stage and more generally the completeness of data on all outcomes at each time point. Participating patients' characteristics (demographics, comorbidities, clinical details) will be summarised and, where possible, compared with the overall population of relevant patients to explore possible factors associated with participation. Where possible, the reasons will be ascertained for potentially eligible patients not being approached to consider participation.

Descriptive data on the clinical outcomes will be presented by trial arm, using appropriate measures of central tendency and variation for continuous measures and numbers/percentages for categorical measures. No formal statistical tests will be conducted.

Qualitative study

For peer review only

For peer review only - http://bmjopen.bmj.com/site/about/guidelines.xhtml

A qualitative evaluation will assess the acceptability of the intervention to women taking part in the study, in particular attitudes towards reinfusion of salvaged blood and transfusion of donor blood. The study will also gain an understanding of the women's experience of taking part in the research processes of the TIC TOC study and what influenced their decision to take part. Following surgery, up to 20 women from across all centres will be asked to take part in individual face to face or telephone semi-structured interviews using a topic guide that has been developed with patient and public involvement (PPI) involvement (Appendix 3). Purposive sampling techniques will ensure a range of women are selected according to centre, education, age, ethnicity, socioeconomic status, and social support.

As the trial schedule allows, the same women will be approached to take part in a brief telephone interview three months after the first interview. The purpose of the second interview is to determine participants' perceptions about the follow-up research processes and ask their opinion about whether anything should change in a full trial. Surgeons from each centre will also be invited to participate in one brief telephone interview each to understand the issues considered in deciding whether to offer women the opportunity to take part in the study.

The qualitative data will be managed using computer software such as Nvivo 11 and thematically analysed (37, 38). The researcher will ensure accuracy of the transcription and read the transcript several times to become immersed in the data, noting initial thoughts and ideas. Codes will be assigned to extracts of the data relevant to the project. Codes with similar meaning will be grouped together in themes. Using constant comparison techniques across the transcripts' themes looking for similarities and differences, the themes will be reviewed and refined. Extracts from the data will be used in the final report. Reflexive research memos will be used as an audit trail of the analysis procedure (39). A second qualitative researcher will conduct an independent analysis of a subset of six transcripts before the researchers meet to discuss and agree the findings. Findings will also be presented to the study's patient advisory group for discussion. Any significant differences of opinion will be discussed with the Chief Investigator. A model may be developed to explain the

For peer review only - http://bmjopen.bmj.com/site/about/guidelines.xhtml

factors affecting recruitment and retention to the trial to inform development of the research processes required in any future full trial.

Economic data and analyses

A definitive study will include a within trial economic evaluation to compare costs and health outcomes of ICS versus donor blood within the time frame of the study and a decision analytic model to extrapolate any future health benefits and costs to the lifetime of the participant. The evaluations will primarily be in relation to quality adjusted life years and will take a health and social perspective on costs, in accordance with NICE guidelines (37). Secondary analyses will take place in relation to important clinical outcomes of interest for the definitive trial such as deaths averted and disease-free progression. This study aims to test the feasibility of collecting enough resource use and outcome data to perform the future economic evaluations.

Data collection tools will be prepared and refined with a view to undertaking the two planned economic evaluations within the future study. These evaluations will take on a health and social care payer perspective. Should participant-reported resource use data allow, the future within-trial economic evaluation will take on a societal perspective on costs in secondary analyses, to further capture the burden to participants, carers, and society. The parameters for the lifetime economic decision model (costs, outcomes, and probabilities of outcomes to occur) will be informed by the within trial economic results. If feasible, costs from a societal perspective may be included in the lifetime economic decision model as well.

Resources will be collected from several sources. In the immediate post-operative period, research nurses will record resources pertaining to the participant's surgery and subsequent hospital stay. Where possible, research staff will also review participants' medical notes at 4.5 months post-operatively to collect hospital contacts following initial discharge (i.e. re-hospitalisations, outpatient and emergency visits). Participant-completed resource use questionnaires will be administered at both six weeks and 4.5 months post-operatively (where the trial schedule allows) to collect other

For peer review only

resources used. These questionnaires will be delivered by post and include questions related to in-patient and out-patient hospital visits; community based services such as General Practice doctor and nurse contacts, physiotherapy, occupational therapy and other community contacts; use of personal social services such as home care workers and social workers; privately paid therapies and expenses; time off work and lost leisure; and informal care required from family and friends. Completion rates, missing data and the method of administering questionnaires will be reviewed to identify potential problems with data collection methods and to seek solutions to minimise participant/staff burden if required. We will report frequency, mean, and standard deviation of resources used by trial arm to explore potential cost-drivers for the main study.

The EuroQoL EQ-5D questionnaire will capture generic quality of life differences between the trial arms. In a recent study of EQ-5D valuation sets, the 3L and 5L versions of the EQ-5D produced substantially different estimates for cost-effectiveness (40) and prompted NICE to issue a position statement in August 2017 to recommend the future use of the 3L version (41). In this study, we will use the mapped utility scores from the 3L to the 5L version using the Van Hout algorithm (42) for the UK population, as recommended by the NICE statement. We expect to use the 3L version in the future study and not proceed with the study of the distribution properties produced by the 5L version scores in this feasibility study.

Patient and Public Involvement and Engagement

The study has benefitted from its inception from an enthusiastic patient advisory group. The aim of PPI in the study is to ensure that the trial is equitable and acceptable to the women taking part by embedding the women's experiential expertise of cancer throughout the trial design and processes. The group comprises six women aged between 50-80 years, who have experienced a cancer diagnosis and are living in Cornwall. However, one member is formerly from Gateshead, where she was treated for her cancer, so is able to bring her experience of the patient pathway to inform the trial processes across the sites. Another member and co-applicant is the founder of PANTS cancer charity in Cornwall.

For peer review only - http://bmjopen.bmj.com/site/about/guidelines.xhtml

The PPI work is undertaken using a predominately collaborative approach with engagement functions embedded within it. The members worked with the research team on the research design and in particular the patient approach, providing input into the grant application, language, content and layout of the participant documentation. The group have worked on the qualitative interview topic guide content and are also working with the qualitative researchers on analysis of the participant interview transcripts. The members are fully integrated into the team and regularly attend the trial management meetings, as well as providing advice and suggesting solutions to problems encountered during the trial.

The members will attend patient and public events and conferences to engage with other members of the public and professionals and share their experience of supporting and being part of the design and management of research. They will also work together with the wider research team to prepare a lay summary of the findings and on other communications such as website, Twitter and Facebook articles.

All members of the research team contribute to the training and support of the PPI members. The mechanisms to achieve these are multifactorial and include specific discussion around methodology and trial processes in PPI meetings, explaining the terminology in lay language, providing information, such as the Involve jargon buster sheet and conducting workshops for specific tasks (e.g. poster development), as well as signposting to other resources such as the Involve website.

ETHICS AND DISSEMINATION

The results of this feasibility study will be published in peer-reviewed journals and presented at relevant national/international conferences and to patient groups. Participants of the trial will be sent a summary of the findings and these will also be disseminated via the pantscancer.org charity, Target Ovarian Cancer charity and participating NHS Trusts' websites.

DISCUSSION

For peer review only

For peer review only - http://bmjopen.bmj.com/site/about/guidelines.xhtml

Research has shown that donor blood transfusions have been associated with poorer outcomes including increased mortality, wound, pulmonary and renal complications; this has been ascribed to transfusion-induced immune modulation (TRIM) (9) which is a transient depression of the immune system following transfusion with blood products. The Cochrane meta-analysis of randomised trials estimated perioperative allogeneic blood transfusion to be associated with increased risk of recurrence with odds ratio of 1.42 (95% CI, 1.20 to 1.67) in surgery for colorectal cancer (43). Long-term results from a clinical trial suggest that this effect of allogeneic blood transfusion is persistent (44, 45). This led to the suggestion of introducing measures that would help limit the use of allogeneic blood transfusion (12).

Patient blood management is an evidence-based patient-tailored approach aimed at reducing the need for allogeneic blood transfusion by managing anaemia, perioperative blood conservation, surgical haemostasis, and drug use (46). Perioperative blood conservation measures include interventions such as the administration of agents to diminish blood loss (e.g. tranexamic acid, fibrin sealant), agents that promote red blood cell production (e.g. erythropoietin) and techniques for reinfusing a patient's own blood including cell salvage (28). Previous randomised and non-randomised studies have provided evidence that the use of intraoperative cell salvage can reduce the need for allogeneic blood transfusion (ABT) (9). A systematic review of 75 randomised trials highlighted that salvaged blood reinfusion reduced the rate of exposure to ABT by 38% (relative risk, 0.62; 95% confidence interval [95% CI], 0.55-0.70) (47). However, concern exists that blood collected by intraoperative cell salvage might result in reinfusion of tumour cells and subsequent distant metastases thus limiting the use of cell salvage across oncological specialties. However, in patients undergoing surgery for a gynaecological malignancy, the use of a leucocyte depletion filter was shown to be effective in eliminating viable nucleated malignant cells from the returned blood during collection, processing, and leukofiltration (27). Similarly, in vitro work shows that depletion filters are highly efficient at removing malignant cells, leading to removal rates of between 80 and 100% (25, 26).

For peer review only - http://bmjopen.bmj.com/site/about/guidelines.xhtml

Patients with primary or metastatic cancer are known to have CTCs in the blood. The concentration of CTCs varies widely depending on tumour type and stage of disease (31). There is evidence from a range of different cancer surgeries that operative manipulation of tumour during surgery leads to peripheral blood concentrations of malignant cells many times higher than could be attained with cell salvage alone (31, 32, 48).

There is emerging evidence suggesting that far from compromising outcomes, intraoperative autologous transfusion is associated with improved outcomes in surgery for other gynaecological cancers such as cervical cancer. Several studies in early stage (I-IIA) cervical cancer patients report that intraoperative autologous transfusion significantly reduces the need for donor blood transfusion, without compromising survival or post-operative complication rates (30). In addition, no distant recurrences have been reported (30). However, most of the evidence on the use of salvaged blood in cancer surgery is based on retrospective and observational studies. These studies are insufficient to draw any definitive conclusions regarding adverse events related to a particular intervention in the presence of multiple confounding factors. Therefore in order to mitigate for confounding factors a large well-designed randomised controlled trials are required (49). Our trial provides new evidence in the use of cell salvage in ovarian cancer surgery and will add to a more general evidence base informing the use of ICS in other areas, in particular other cancers.

For peer review only

Conflict of interest statement

The authors declare no conflict of interest.

This work was supported by the National Institute for Health Research (NIHR) Research for Patient Benefit (RfPB) programme, grant number PB-PG-1014-35005.

Contributorship statement

All authors except NF and JP were co-applicants on the NIHR RfPB grant application and as such were involved in the design of this feasibility study. All authors contributed to successive drafts of this paper.

For peer review only - http://bmjopen.bmj.com/site/about/guidelines.xhtml

KG is the Chief Investigator, provided clinical expertise and was responsible for conception and design of the study as well as drafting and revising of the article.

NF was responsible for the first draft of this paper.

CP contributed to study design.

AB contributed to study design and trial management.

PE is the trial statistician and provided expertise in the overall design of the trial.

JF provided expertise in cell salvage and drafted the cell salvage protocol.

AL provided clinical expertise and helped with design of the study.

EM was responsible for the design and analysis of the economic evaluation component.

JP contributed to study design and coordinated the PPI input.

CR provided anaesthetics and cell salvage expertise

JV is the trial manager, responsible for overseeing the day-to-day running of the trial.

JW was responsible for the design and conduct of the qualitative study.

Acknowledgements

The authors are grateful for the support of the study sponsor (Royal Cornwall Hospitals NHS Trust) and the South West Peninsula NIHR Clinical Research Network. We are also indebted to the members of our PPI group for their continued support of the trial. In addition, the authors would like to thank the following members of the TIC TOC trial team: Mr S Chatopadhyay, Mr G Hughes, Mr R Naik and Dr P Ricketts.

DH disclaimer

The views expressed are those of the author(s) and not necessarily those of the NHS, the NIHR or the Department of Health.

For peer review only

For peer review only

References

1. Cancer Research UK. Ovarian cancer incidence statistics http://www.cancerresearchuk.org/cancer-; 2014.
2. National Cancer Intelligence Network (NCIN). Overview of Ovarian Cancer in England: Incidence, Mortality and Survival. Trent Cancer Registry November 2012 [Internet]. 2012.
3. Schwartz PE. Cytoreductive surgery in the management of ovarian cancer Oncology 22: 1025-33. 2008;22(9):1025-33 .
4. Peiretti M ZV, Aletti GD, Bocciolone L, Colombo N, Landoni F, Minig L, Biffi R, Radice D, Maggioni A. Role of maximal primary cytoreductive surgery in patients with advanced epithelial ovarian and tubal cancer: Surgical and oncological outcomes. Single institution experience. Gynecol Oncol. 2010;119(2):256-64.
5. Prescott LS AT, Brown AJ, Taylor JS, Munsell MF, Sun CC, et al,. Perioperative blood transfusion in gynecologic oncology surgery: analysis of the National Surgical Quality Improvement Program Database. Gynecologic oncology. 2015;136(1):65-70.
6. Warner LL DS, Martin JR, Lemens MA, McGree ME, Weaver AL, et al,. The impact of perioperative packed red blood cell transfusion on survival in epithelial ovarian cancer. . Int J Gynecol Cancer 2013;23:1612–9.
7. Ferraris VA DD, Saha SP, Austin PC, Zwischenberger JB. Surgical Outcomes and Transfusion of Minimal Amounts of Blood in the Operating Room. JAMA Surgery. Jan 2012, Vol 147, No. 1 JAMA Surgery. 2012;147(1):49-55.
8. PHB Bolton-Maggs (Ed) DP, A Watt, D Thomas and H Cohen on behalf of the Serious Hazards of Transfusion (SHOT) Steering Group. The 2012 Annual SHOT Report (2013)
9. Blajchman MA, and Eleftherios C. Vamvakas. "Transfusion-Related Immunomodulation." Practical Transfusion Medicine. Third Edition (2009): 98-106. 2009.
10. Goubran H SD, Radosevic J, Burnouf T, Seghatchian J,. Transfusion-related immunomodulation and cancer. Transfus Apher Sci. 2017;56(3):336–40.
11. Ashworth A KAA. Cell salvage as part of a blood conservation strategy in anaesthesia. Br J Anaesth. 2010;105(4):401-16.
12. The Association of Anaesthetists of Great Britain and Ireland (AAGBI). The Association of Anaesthetists of Great Britain and Ireland (AAGBI) Safety Guideline—Blood Transfusion and the Anaesthetist: Intra-operative Cell Salvage2009.
13. Krajewski K AR, Pung N, Wald S, Lazareff J, Kawamoto HK, Bradley JP. Successful blood conservation during craniosynostotic correction with dual therapy using Procrit and cell saver. J Caraniofac Surg. 2008;19:101-5.
14. Bridgens JP EC, Dobson PMS, Hamer AJ. Intraoperative red blood-cell salvage in revision hip surgery. J Bone Joint Surg 2008;89:270-5.
15. Colwell CW Jr. BE, West C, Hardwick ME, Morris BA. Erythrocyte viability in blood salvaged during total joint arthroplasty with cement. J Bone Joint Surg 2002;84:23-5.
16. Williamson KR TH. Intraoperative blood salvage: a review. Transfusion. 1991;31(7):662-75.
17. Akbulut S KC, Yilmaz M, Ince V, Ozgor D, Karabulut K, Eris C, Toprak HI, Aydin C, Yilmaz S. Effect of autotransfusion system on tumor recurrence and survival in hepatocellular carcinoma patients World J Gastroenterol. 2013;19(10):1625–31.
18. Wang G BD, Martin J, Cheng D The efficacy of an intraoperative cell saver during cardiac surgery: a meta-analysis of randomized trials. Anesth Analg. 2009;109(2):320-30.
19. Marković M DL, Savić N, Sindjelić R, Ille T, Dragas M. Intraoperative cell salvage versus allogeneic transfusion during abdominal aortic surgery: clinical and financial outcomes. Vascular. 2009;17(2):83-92.
20. Breakwell LM GC, Dobson P. T,. The efficacy of autologous blood transfusion in bilateral total knee arthroplasty. Knee. 2000;7(3):145–7.

For peer review only

21. DeAndrade D WJ, Triulzi DJ, Alarcon L, Wisniewski MK, Dyga R, et al,. Very low rate of patient-related adverse events associated with the use of intraoperative cell salvage. Transfusion. 2016 Nov;56(11):2768–72. Transfusion. 2016;56(11):2768–72.
22. Hansen E WN, Kneuchel R, Ruschoff J, Hofstaedter F. Hansen E, Wolff N, Kneuchel R, Ruschoff J, Hofstaedter F. Tumor calls in blood shed from the surgical field. Arch.Surg. 1995; 130(4) 387-393. Tumor calls in blood shed from the surgical field. 1995;130(4):387-93.
23. Kumar N ZA, Kantharajanna SB, Khoo BL, Lim CT, Thiery JP,. Metastatic efficiency of tumour cells can be impaired by intraoperative cell salvage process: truth or conjecture? . Transfus Med. 2017;Suppl 5:327-34.
24. Motoyama S SR, Kamata S, Kitamura M, Okuyama M, Imano H, Nakamura M, Suzuki H, Omokawa S, Motohashi Y, Ogawa J. Survival advantage of using autologous blood transfusion during surgery for esophageal cancer. Surg Today. 2002;32(11):951-8.
25. Karczewski DM LM, Glaves D. The efficiency of an autotransfusion system for tumor cell removal from blood salvaged during cancer surgery. Anesth Analg 1994;78(6):1131-5.
26. Fruhauf NR DO, Kaudel CP, Kasimir-Bauer S, Oldhafer KJ. Filtration of malignant cells: tumour cell depletion in an ex vivo model using a leukocyte adhesion filter. Perfusion 2001;16:Suppl:51-5.
27. Catling S WS, Freites O, Rees M, Davies C, Hopkins L. Use of a leucocyte filter to remove tumour cells from intra-operative cell salvage blood. Anaesthesia 2008;63(12):1332-8.
28. Zaw AS BKS, Kumar N,. Is Autologous Salvaged Blood a Viable Option for Patient Blood Management in Oncologic Surgery? . Transfusion Medicine Reviews. 2017;31(1):56-61.
29. Connor JP MP, Alagoz T, Anderson B, Bottles K, Buller RE. Intraoperative autologous blood collection and autotransfusion in the surgical management of early cancers of the uterine cervix. Obstet Gynecol. 1995;86(3):373- 8.
30. Mirhashemi R AH, Deepika K, Estape R, Angioli R, Martin J, Rodriguez M, Penalver MA. The impact of intraoperative autologous blood transfusion during type III radical hysterectomy for early-stage cervical cancer. Am J Obstet Gynecol 1999;181(6):1310-5; discussion 5-6.
31. AllardWJ MJ, MillerMC, Repollet M, Connelly MC, Rao C, et al,. Tumor cells circulate in the peripheral blood of all major carcinomas but not in healthy subjects or patients with nonmalignant diseases. Clin Cancer Res. 2004;10:6897–904.
32. Oefelein MG KK, Herz B, Blum MD, Holland JM, Keeler TC, Cook WA, Ignatoff JM. Molecular detection of prostate epithelial cells from the surgical field and peripheral circulation during radical prostatectomy. J Urol 1996;155(1):238-42.
33. Allan AL KM. Circulating tumor cell analysis: technical and statistical considerations

for application to the clinic. J Oncol 2010;2010:426218.
34. Galaal K BA, Smits A, Lopes AD,. Intraoperative Cell Salvage in Surgery for Ovarian Cancer: Time for Action. . Medical Journal of Obstetrics and Gynecology 2014;2(2):1035.
35. Society of Gynecologic Oncology. FIGO ovarian cancer staging: https://www.sgo.org/wp-content/uploads/2012/FIGO-Ovarian-Cancer-Staging_1.10.14.pdf; 2014 [
36. Oken MM, Creech, R.H., Tormey, D.C., Horton, J., Davis, T.E., McFadden, E.T., Carbone, P.P. Toxicity And Response Criteria Of The Eastern Cooperative Oncology Group. Am J Clin Oncol 1982;5:649-55.
37. Braun V CV. Using thematic analysis in psychology. Qualitative Research in Psychology. 2006;3:77-101.
38. Green J TN. 2004. Qualitative Health Methods for Health Research. London: Sage Publications.
39. Liamputtong P ED. Qualitative Research Methods. Second Edition ed. Oxford: Oxford University Press; 2005. In: Press OU, editor. Oxford:2005.
40. Wailoo A HAM, Grimm S et al. Comparing the EQ-5D-3L and 5L versions. What are the implications for cost effectiveness estimates? NICE Decision Support Unit report 2017 6th July 2017.

For peer review only

41. https://www.nice.org.uk/Media/Default/About/what-we-do/NICE-guidance/NICE-technology-appraisal-guidance/eq5d5l_nice_position_statement.pdf.
42. Van Hout B JM, Feng Y et al,. Interim scoring for the EQ-5D-5L: Mapping the EQ-5D-5L to EQ-5D-3L value sets. Value in Health. 2012;15:708-15.
43. Amato A PM. Perioperative blood transfusions and recurrence of colorectal cancer. Cochrane Database of Systematic Reviews 2006, Issue 1. Art. No.: CD005033. DOI: 10.1002/14651858.CD005033.pub2. Cochrane Database of Systematic Reviews. 2006(1).
44. Linder BJ FI, Cheville JC, Tollefson MK, Thompson RH, Tarrell RF, Thapa P, Boorjian SA. The Impact of Perioperative Blood Transfusion on Cancer Recurrence and Survival Following Radical Cystectomy Eur Urol. 2013;63(5):839–45
45. Harlaar JJ GM, Hop WC, Lange JF, Busch OR, Jeekel H Blood Transfusions and Prognosis in Colorectal Cancer: Long-term Results of a Randomized Controlled Trial Ann Surg. 2012;256:681-6
46. Spahn DR SA, Hofmann A,. The chiasm: transfusion practice versus patient blood management. Best Pract Res Clin Anaesthesiol 2013;27:37-42.
47. Carless PA HD, Moxey AJ, O'Connell D, Brown T, Fergusson DA. Cell salvage for minimising perioperative allogeneic blood transfusion. Cochrane Database of Systematic Reviews 2010(4):Art. No: CD001888.
48. Yamaguchi K TY, Aoki S, FutamuraM, Saji S,. Significant detection of circulating cancer cells in the blood by reverse transcriptase-polymerase chain reaction during colorectal cancer resection. Ann Surg 2000;232:58-65.
49. Yazer MH TD. Things aren't always as they seem: what the randomized trialsof red blood cell transfusion tell us about adverse outcomes. Transfusion 2014;54:3243–6 [quiz 2].

For peer review only

For peer review only - http://bmjopen.bmj.com/site/about/guidelines.xhtml

Legend

Table 1 Trial schedule

Figure 1 Summary of trial design

Appendix 1 FIGO Ovarian Cancer Staging

Appendix 2 Definition of surgical site infection

Appendix 3 Topic guide for participant interviews

For peer review only

TABLES AND FIGURES

Table 1: Trial schedule

	Pre-operative		OPERATION and peri-operative data collection	Post-operative follow-up				
	Screen	Baseline		1	2	3†	4†	5†
				30 days post-op	6 weeks post-op	3 months after follow-up 2	6 months after follow-up 2	9 months after follow-up 2
Screen/eligibility	x							
Consent		x						
Demographics & history		x						
Randomisation		x						
EORTC QLQ-C30*		x			x	x	x	x
EORTC QLQ-OV28*		x			x	x	x	x
EQ-5D-5L		x			x	x	x	x
Adverse events				x				
Resource use questionnaire		x			x	x		
Qualitative interviews					x	x		

† As time allows

*Confirmed cancer participants only for post-operative follow-up

For peer review only

For peer review only - http://bmjopen.bmj.com/site/about/guidelines.xhtml

Figure 1: Summary of trial design

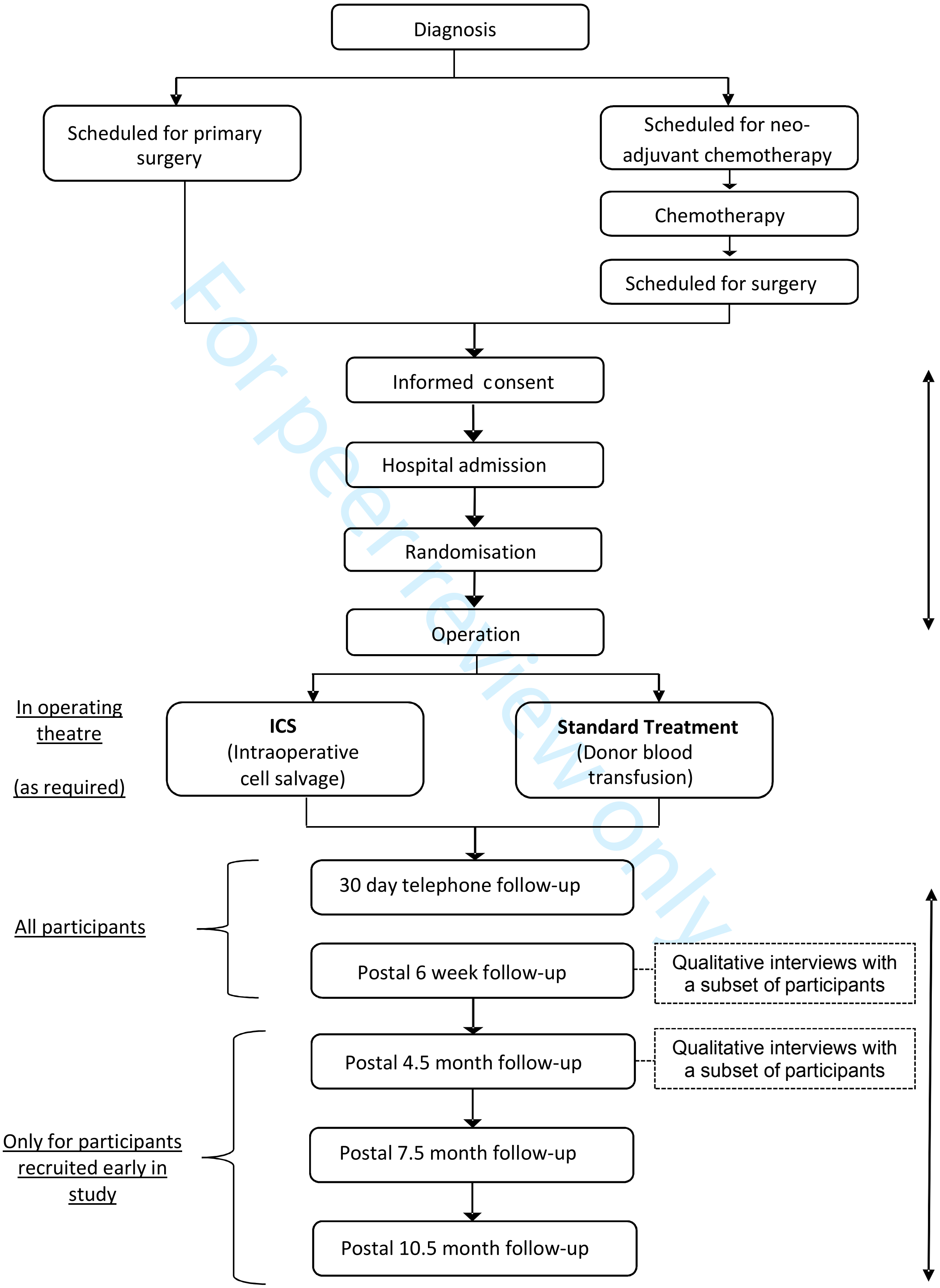

For peer review only

For peer review only - http://bmjopen.bmj.com/site/about/guidelines.xhtml

Appendix 1: FIGO Ovarian Cancer Staging

Effective 1 January 2014

**STAGE I: Tumour confined to ovaries**	
**IA**	Tumour limited to one ovary, capsule intact, no tumour on surface, negative washings
**IB**	Tumour involves both ovaries, otherwise like IA
**IC**	Tumour limited to one or both ovaries
IC1	Surgical spill
IC2	Capsule rupture before surgery or tumour on ovarian surface
IC3	Malignant cells in the ascites or peritoneal washings

**STAGE II: Tumour involves one or both ovaries with pelvic extension (below the pelvic brim) or primary peritoneal cancer**	
**IIA**	Extension and/or implant on uterus and/or Fallopian tubes
**IIB**	Extension to other pelvic intraperitoneal tissues

<table>
<tr><th colspan="3">STAGE III: Tumour involves 1 or both ovaries with cytologically or histologically confirmed spread to the peritoneum outside the pelvis and/or metastasis to the retroperitoneal lymph nodes</th></tr>
<tr><td>IIIA</td><td colspan="2">Positive retroperitoneal lymph nodes and/or microscopic metastasis beyond the pelvis</td></tr>
<tr><td rowspan="3">IIIA1</td><td colspan="2">Positive retroperitoneal lymph nodes only</td></tr>
<tr><td>IIIA1 (i)</td><td>Metastasis ≤ 10mm</td></tr>
<tr><td>IIIA1 (ii)</td><td>Metastasis > 10mm</td></tr>
<tr><td>IIIA2</td><td colspan="2">Microscopic, extrapelvic (above the brim) peritoneal involvement ± positive retroperitoneal lymph nodes</td></tr>
<tr><td>IIIB</td><td colspan="2">Macroscopic, extrapelvic, peritoneal metastasis ≤ 2cm ± positive retroperitoneal lymph nodes. Includes extension to capsule of liver/spleen</td></tr>
<tr><td>IIIC</td><td colspan="2">Macroscopic, extrapelvic, peritoneal metastasis > 2cm ± positive retroperitoneal lymph nodes. Includes extension to capsule of liver/spleen</td></tr>
</table>

**STAGE IV: Distant metastasis excluding peritoneal metastasis**	
IVA	Pleural effusion with positive cytology
IVB	Hepatic and/or splenic parenchymal metastasis, metastasis to extra-abdominal organs (including inguinal lymph nodes and lymph nodes outside of the abdominal cavity)

Other major recommendations are as follows:

- Histologic type including grading should be designated at staging
- Primary site (ovary, Fallopian tube or peritoneum) should be designated where possible
- Tumours that may otherwise qualify for stage I but involved with dense adhesions justify upgrading to stage II if tumour cells are histologically proven to be present in the adhesions.

For peer review only

Appendix 2: Definition of surgical site infection

For the purposes of this study, surgical site infection (48, 49) is defined as an infection that:-

i) occurs within 30 days after the operation and

ii) appears to be related to the operation and

iii) involves deep soft tissues (e.g. fascial and muscle layers) of the incision and at least one of the following:-

a) Purulent drainage from the deep incision but not from the organ/space component of the surgical site

b) A deep incision spontaneously dehisces or is deliberately opened by a surgeon when the patient has at least one of the following signs or symptoms: fever (> 38 C), localized pain, or tenderness, unless site is culture-negative.

c) An abscess or other evidence of infection involving the deep incision is found on direct examination, during reoperation, or by histopathologic or radiologic examination.

d) Diagnosis of a deep incision SSI by a surgeon or attending physician

For peer review only

For peer review only - http://bmjopen.bmj.com/site/about/guidelines.xhtml

Appendix 3: Topic guide for participant interviews

First qualitative interview (6 weeks)

Topic	Questions	Prompts
**Opening question**	How are you feeling after your operation? Tell me a bit about yourself?	Role in life – past or present employment<br>Family<br>Be sensitive and understanding
**Recruitment**	How were you approached to take part in the TIC TOC study?<br>What did you think about the way the study was introduced?<br>What did you understand about the study?<br>What questions did you have? Did you receive answers you understood?	Which member of staff, how approached(surgeon, specialist nurse)
**Specific understanding**	What did you understand about reintroducing your own blood?<br>What did you understand by donor blood transfusion?	Which method did you think was safest?
**Involvement of family and friends**	Did you ask anyone else for their opinions?<br>If yes, who were they?<br>What was their opinion?	Explore any negative responses from family and friends<br>Explore any positive responses from family and friends
**Decision process**	What things did you think about when deciding if you were going to take part?	Barriers<br>Factors that stopped the woman taking part (fear, overwhelmed by potential cancer diagnosis, chance would get cell salvage anyway (some sites), lack of understanding, unable to read research literature)<br>Facilitators<br>Factors that encouraged her to take part (trust of surgeon, research staff, feeling obligated, fear, distrust of donated blood or salvaged blood)
**Research processes**	When you came to the first clinic to see your consultant, how were you treated in the research part of your appointment?<br>Tell me what you felt about the specialist nurse asking you if you wanted to take part in the TIC TOC study?<br>What did you think about the timing of being recruited to the study?<br>What did you think about the questionnaires?	Check woman's talk is about the research.<br>Woman may want to talk about their cancer experience – allow it.<br>Baseline questionnaires only
**Allocation**	Which group do you think you were	Do not say which

For peer review only - http://bmjopen.bmj.com/site/about/guidelines.xhtml

	allocated?<br><br>Why?	
**Information about next appointments**	As part of your normal care, you will be followed up by your consultant or his/her team. As part of the research study you will receive some further postal questionnaires.<br><br>Can I contact you again in about 6 months to see what you think about the postal follow-up?	

Second qualitative interview (three months after first, by telephone)

**Topic**	**Questions**	**Prompts**
**Opening question**	Since we last spoke, how have you been getting on?<br><br>I have a few questions to ask you about your experience of taking part in the TIC TOC study.	May not be feeling well.<br><br>May be on chemotherapy treatment.<br><br>Be sensitive and understanding
**Research process: follow-up questionnaires**	Where did you complete your questionnaires?<br><br>Did you have help to complete the questionnaires?<br><br>What did you like about the telephone/postal follow up?<br><br>What didn't you like about the telephone/postal follow up?<br><br>Was there anything that could be improved?<br><br>Did you know who to contact if you did not wish to keep taking part?<br><br>What did you think about the questionnaires asking you what health services you had used?	Did the woman know how to make a complaint?<br><br>(probe questionnaires by telephone) Check view about the number of questionnaires and clarity of questions<br><br>Check for questionnaire burden
**Allocation**	Which group do you think you were allocated?<br><br>Why?	**Do not say which.**<br>**The woman will receive notification about the allocation at the end of the study.**
	Thank you for taking part in the research study that will help inform a larger study.<br><br>Wish well for the future.	

For peer review only - http://bmjopen.bmj.com/site/about/guidelines.xhtml

Contribution statement

All authors except NF and JP were co-applicants on the NIHR RfPB grant application and as such were involved in the design of this feasibility study. All authors contributed to successive drafts of this paper.

KG is the Chief Investigator, provided clinical expertise and was responsible for conception and design of the study as well as drafting and revising of the article.

NF was responsible for the first draft of this paper.

AB: contributed to study design and trial management.

PE: is the trial statistician and provided expertise in the overall design of the trial.

JF: provided expertise in cell salvage and drafted the cell salvage protocol.

AL: provided clinical expertise and helped with design of the study.

EM: was responsible for the design and analysis of the economic evaluation component.

JP: contributed to study design and coordinated the PPI input.

CR: provided anaesthetics and cell salvage expertise

JV is the trial manager, responsible for overseeing the day-to-day running of the trial.

JW was responsible for the design and conduct of the qualitative study.

For peer review only - http://bmjopen.bmj.com/site/about/guidelines.xhtml

CONSORT 2010 checklist of information to include when reporting a randomised trial*

Section/Topic	Item No	Checklist item	Reported on page No
**Title and abstract**			
	1a	Identification as a randomised trial in the title	1
	1b	Structured summary of trial design, methods, results, and conclusions (for specific guidance see CONSORT for abstracts)	2
**Introduction**			
Background and objectives	2a	Scientific background and explanation of rationale	4-5
	2b	Specific objectives or hypotheses	6
**Methods**			
Trial design	3a	Description of trial design (such as parallel, factorial) including allocation ratio	6
	3b	Important changes to methods after trial commencement (such as eligibility criteria), with reasons	N/A
Participants	4a	Eligibility criteria for participants	7-8
	4b	Settings and locations where the data were collected	7
Interventions	5	The interventions for each group with sufficient details to allow replication, including how and when they were actually administered	8-9
Outcomes	6a	Completely defined pre-specified primary and secondary outcome measures, including how and when they were assessed	11-12
	6b	Any changes to trial outcomes after the trial commenced, with reasons	N/A
Sample size	7a	How sample size was determined	12
	7b	When applicable, explanation of any interim analyses and stopping guidelines	N/A
Randomisation:			
Sequence generation	8a	Method used to generate the random allocation sequence	8
	8b	Type of randomisation; details of any restriction (such as blocking and block size)	8
Allocation concealment mechanism	9	Mechanism used to implement the random allocation sequence (such as sequentially numbered containers), describing any steps taken to conceal the sequence until interventions were assigned	8
Implementation	10	Who generated the random allocation sequence, who enrolled participants, and who assigned participants to interventions	7, 8
Blinding	11a	If done, who was blinded after assignment to interventions (for example, participants, care providers, those	9

For peer review only

For peer review only - http://bmjopen.bmj.com/site/about/guidelines.xhtml

		assessing outcomes) and how	
	11b	If relevant, description of the similarity of interventions	N/A
Statistical methods	12a	Statistical methods used to compare groups for primary and secondary outcomes	12-14
	12b	Methods for additional analyses, such as subgroup analyses and adjusted analyses	N/A
**Results**			
Participant flow (a diagram is strongly recommended)	13a	For each group, the numbers of participants who were randomly assigned, received intended treatment, and were analysed for the primary outcome	N/A
	13b	For each group, losses and exclusions after randomisation, together with reasons	N/A
Recruitment	14a	Dates defining the periods of recruitment and follow-up	N/A
	14b	Why the trial ended or was stopped	N/A
Baseline data	15	A table showing baseline demographic and clinical characteristics for each group	N/A
Numbers analysed	16	For each group, number of participants (denominator) included in each analysis and whether the analysis was by original assigned groups	N/A
Outcomes and estimation	17a	For each primary and secondary outcome, results for each group, and the estimated effect size and its precision (such as 95% confidence interval)	N/A
	17b	For binary outcomes, presentation of both absolute and relative effect sizes is recommended	N/A
Ancillary analyses	18	Results of any other analyses performed, including subgroup analyses and adjusted analyses, distinguishing pre-specified from exploratory	N/A
Harms	19	All important harms or unintended effects in each group (for specific guidance see CONSORT for harms)	N/A
**Discussion**			
Limitations	20	Trial limitations, addressing sources of potential bias, imprecision, and, if relevant, multiplicity of analyses	N/A
Generalisability	21	Generalisability (external validity, applicability) of the trial findings	N/A
Interpretation	22	Interpretation consistent with results, balancing benefits and harms, and considering other relevant evidence	N/A
**Other information**			
Registration	23	Registration number and name of trial registry	2
Protocol	24	Where the full trial protocol can be accessed, if available	N/A
Funding	25	Sources of funding and other support (such as supply of drugs), role of funders	19

*We strongly recommend reading this statement in conjunction with the CONSORT 2010 Explanation and Elaboration for important clarifications on all the items. If relevant, we also recommend reading CONSORT extensions for cluster randomised trials, non-inferiority and equivalence trials, non-pharmacological treatments, herbal interventions, and pragmatic trials. Additional extensions are forthcoming: for those and for up to date references relevant to this checklist, see www.consort-statement.org.

For peer review only - http://bmjopen.bmj.com/site/about/guidelines.xhtml

BMJ Open

Trial of intraoperative cell salvage versus transfusion in ovarian cancer (TIC TOC): protocol for a randomised controlled feasibility study

Journal:	*BMJ Open*
Manuscript ID	bmjopen-2018-024108.R1
Article Type:	Protocol
Date Submitted by the Author:	31-Jul-2018
Complete List of Authors:	Galaal, Khadra; Royal Cornwall Hospitals NHS Trust, Gynaeoncology<br>Lopes, Alberto; Royal Cornwall Hospitals NHS Trust<br>Pritchard, Colin; Royal Cornwall Hospitals NHS Trust, Research, Development and Innovation<br>Barton, Andy; NIHR South West Research Design Service<br>Wingham, Jennifer; University of Exeter Medical School,<br>Marques, Elsa; University of Bristol, School of Social and Community Medicine<br>Faulds, John; Royal Cornwall Hospitals NHS Trust<br>Palmer, Joanne; Hull York Medical School, Vascular Research Unit<br>Vickery, Patricia; Plymouth University, Peninsula Clinical Trials Unit<br>Ralph, Catherine; Royal Cornwall Hospitals NHS Trust, Anaesthetics<br>Ferreira, Nicole ; Royal Cornwall Hospitals NHS Trust<br>Ewings, Paul; Research Design Service, Research Office
**Primary Subject Heading**:	Obstetrics and gynaecology
Secondary Subject Heading:	Haematology (incl blood transfusion)
Keywords:	intraoperative cell salvage, donor blood transfusion, cytoreductive surgery, ovarian cancer, feasibility trial, quality of life

SCHOLARONE™
Manuscripts

Trial of intraoperative cell salvage versus transfusion in ovarian cancer (TIC TOC): protocol for a randomised controlled feasibility study

Khadra Galaal[1], Alberto Lopes[2], Colin Pritchard[3], Andy Barton[4], Jennifer Wingham[2], Elsa Marques[5], John Faulds[6], Joanne Palmer[3], Jane Vickery[7], Catherine Ralph[9], Nicole Ferreira[6], Paul Ewings[4].

1. Gynaecology, Royal Cornwall Hospitals NHS Trust, Truro, Cornwall, UK
2. University of Exeter, Devon, UK
3. Research, Development and Innovation, Royal Cornwall Hospitals NHS Trust, Truro, Cornwall, UK
4. NIHR Research Design Service (South West)
5. Bristol Medical School, University of Bristol, Bristol, UK
6. Royal Cornwall Hospitals NHS Trust, Truro, Cornwall, UK
7. Peninsula Clinical Trials Unit, Plymouth University, Plymouth, UK
8. Department of Anaesthesia, Royal Cornwall Hospitals NHS Trust, Truro, Cornwall, UK.

Corresponding Author

Miss Khadra Galaal FRCOG

Consultant Gynaecological Oncologist

Royal Cornwall Hospital, Truro, UK

Tel: 01872 252731

Email: k.galaal@nhs.net

Key words: intraoperative cell salvage, donor blood transfusion, cytoreductive surgery, ovarian cancer, feasibility trial, economic evaluation, quality of life.

For peer review only

ABSTRACT

Introduction: Ovarian cancer is the leading cause of death from gynaecological cancer, with more than 7,000 new cases registered in the UK in 2014. In patients suitable for surgery, NICE guidance for treatment recommends surgical resection of all macroscopic tumour, followed by chemotherapy. The surgical procedure can be extensive and associated with substantial blood loss which is conventionally replaced with a donor (allogeneic) blood transfusion. Whilst often necessary and life-saving, the use of donor (allogeneic) blood is associated with increased risks of complications and adverse surgical outcomes. Intraoperative cell salvage (ICS) is a blood conservation strategy in which red cells collected from blood lost during surgery are returned to the patient thus minimising the use of donor blood. This is the protocol for a feasibility randomised controlled trial with an embedded qualitative study and feasibility economic evaluation. If feasible, a later definitive trial will test the effectiveness and cost-effectiveness of ICS reinfusion versus donor (allogeneic) blood transfusion in ovarian cancer surgery.

Methods and analysis: Sixty adult females scheduled for primary or interval ovarian cancer surgery at participating UK NHS Trusts will be recruited and individually randomised in a 1:1 ratio to receive intraoperative cell salvage reinfusion or donor blood (as required) during surgery. Participants will be followed up by telephone at 30 days post-operatively for adverse events monitoring and by postal questionnaire at six weeks and three monthly thereafter, to capture quality of life and resource use data. Qualitative interviews will capture participants' and clinicians' experiences of the study.

Ethics and dissemination: This study has been granted ethical approval by the South West - Exeter Research Ethics Committee (ref: 16/SW/0256). Results will be disseminated via peer-reviewed publications and will inform the design of a larger trial.

Trial registration number: ISRCTN19517317

For peer review only - http://bmjopen.bmj.com/site/about/guidelines.xhtml

Strengths and limitations of this study

- This is the first study to use intraoperative cell salvage in cytoreductive surgery for ovarian cancer
- The study explores the feasibility and informs the design of a larger randomised controlled trial. Quantitative, qualitative and feasibility economic components are included
- Limitations are;
  - The effect of transfusion and cell salvage on immune response to surgery is not assessed
  - This feasibility study will not provide information on the long-term outcomes of using either cell salvage or transfusion.

For peer review only

INTRODUCTION

Background

Ovarian cancer is the leading cause of death from gynaecological cancer in the UK (age-standardised mortality rate 9.1 per 100,000 2008-2010) (1). Although survival rates have improved in recent decades, there are still more deaths from ovarian cancer than all other gynaecological cancers combined (2). The mainstays of treatment for advanced ovarian cancer are surgical cytoreduction and platinum-based chemotherapy. Surgery includes total abdominal hysterectomy and bilateral salpingo-oophorectomy with omentectomy and debulking of as much gross tumour as can safely be completed. Current evidence indicates that the volume of visible disease left at the completion of the primary surgery is related to patient survival The extent of surgery involves a trade-off between the benefits of minimising residual disease and the risks of operative morbidity~~As operative success and survival is largely determined by residual disease~~ (3, 4). Surgery is often extensive with substantial intraoperative blood loss, about 53% of patients lose more than 1.5 litres during their first surgery (5). Blood lost during surgery is conventionally replaced using donor blood transfusion with the incidence of transfusion ranging from 35% to 77% (6, 7). Perioperative donor blood transfusion is associated with increased risks of complications and adverse surgical outcomes including mortality, wound infection, pulmonary and renal complications, systemic sepsis and prolonged hospital stay (8). In 2012 there were 12.3 serious adverse incidents per 10,000 transfused components reported by the Serious Hazards of Transfusion (SHOT) group (9). SHOT is an independent, professionally-led scheme, involved in collecting and analysing anonymised information on adverse events and reactions in blood transfusion from all healthcare organisations in the U.K. Where risks and problems are identified, they produce recommendations to improve patient safety. One suggested explanation for adverse reactions is a general transient depression of the immune system following transfusion with blood products, transfusion-induced immunomodulation (TRIM) (10, 11).

Intraoperative cell salvage (ICS) or autologous blood transfusion is the practice of recovering red cells from blood lost in the operative field and returning them to the patient (12). This process

For peer review only - http://bmjopen.bmj.com/site/about/guidelines.xhtml

involves the separation, centrifugation, washing and filtration of heparinised red blood cells, before reinfusion into the patient. ICS eliminates or reduces the need for donor blood transfusion and its associated risks, making it an alternative where major blood loss is anticipated (13). ICS can be available in theatre at modest expense and reduces dependence on the limited pool of banked blood. Studies comparing cell salvage with allogeneic blood transfusion have demonstrated increased mean erythrocyte (red blood cells) viability as high as 88% with cell salvage (14-16). ICS has been used successfully in surgical specialties (17) including cardiothoracic, vascular, orthopaedic and hepatobiliary (18-21). In addition, intraoperative cell salvage is associated with low rate of patient-related adverse events (22). ICS was initially contraindicated in cancer because of the theoretical risk of reintroducing malignant tumour cells into patients' bloodstreams (23, 24). However, such concerns appear to be unfounded(25). The *in vitro,* leucocyte depletion filters are highly efficient at removing malignant cells with removal rates of between 80 and 100% (26, 27). In patients undergoing surgery for gynaecological malignancy, leucocyte depletion filters effectively eliminate viable nucleated malignant cells from the returned blood (28, 29). Far from compromising outcomes, ICS is associated with improved outcomes in cervical (30, 31) and~~-~~ prostate~~oesophageal~~ cancers (32, 33).

Interestingly, patients with primary metastatic cancer are known to have circulating tumour cells in the blood (34). Furthermore, operative manipulation of tumours during surgery leads to peripheral blood concentrations of malignant cells many times higher than could be attained with cell salvage (35, 36). The presence of circulating tumour cells is prevalent in cancer patients with approximately one circulating tumour cell (CTC) per 105 to 107 mononuclear cells found in the peripheral blood of metastatic cancer patients (37).

Rationale

There is a paucity of studies in ICS, making it difficult for patients, clinicians and NHS managers to make decisions about this technology (38). ICS has been used in ovarian cancer patients in one of the participating sites with encouraging results, but a randomised controlled trial (RCT) is required

For peer review only - http://bmjopen.bmj.com/site/about/guidelines.xhtml

for robust determination of effectiveness. The aim of a definitive trial would be to assess the clinical and cost-effectiveness of intraoperative cell salvage for women undergoing cytoreductive surgery for ovarian cancer, compared with usual practice of transfusing only allogeneic blood as required.

Aim and objectives

The aim of the study is to determine whether a definitive randomised controlled trial is feasible and, if so, how best to deliver it. The objectives of the study are to:

- Estimate the likely recruitment rate for the larger trial
- Estimate the likely completeness of resource use and outcome data
- Explore the practical logistics of undertaking randomisation in theatres
- Assess success of blinding of allocation for participants and outcome assessors
- Design data collection tools to collect resource use data from participants, hospital medical records and hospital staff
- Inform the trial design and confirm the resources required to run a larger definitive trial
- Explore the barriers and facilitators for women when deciding whether or not to participate
- Explore women's perceptions of:
    - The intervention, the information given and advantages/disadvantages of participation so that information can be optimised for the larger trial
    - Other trial aspects, e.g. regarding collection of outcome measures and completing resource use questionnaires.
- Identify factors influencing surgeons' decisions about whether or not to participate in the study.

METHODS

Trial design

For peer review only

This is a protocol for a randomised, controlled, multi-centre feasibility study in women undergoing cytoreductive surgery for ovarian cancer. Sixty participants will be individually randomised in a 1:1 ratio to intraoperative cell salvage (re-infusion of their own blood) or donor blood transfusion during surgery. Participants and outcome assessors will be blinded to the intervention. All participants will be followed up by telephone for adverse events reporting at 30 days post-operatively, by post six weeks post-operatively and three monthly thereafter as time allows. A schematic diagram of the trial is given in Figure 1. The feasibility study includes an embedded qualitative component to assess participants' (patients and clinicians) perceptions of their experience in preparation for the later trial. It will also involve an assessment of the feasibility of collecting resource use and other economic data for a future economic evaluation.

Study setting

The study will take place at the Royal Cornwall Hospitals NHS Trust, Plymouth Hospitals NHS Trust, Gateshead Health NHS Foundation Trust and University Hospitals of Leicester NHS Trust. All sites have existing personnel experienced in the management of intraoperative cell salvage and reinfusion.

Participants and recruitment

Participants will be recruited from patients scheduled to undergo surgery for ovarian cancer at the participating hospitals. Potential participants will usually be identified from those patients attending the gynaecological oncology out-patient clinic having been referred by their GP under the two ~~week~~weeks wait cancer pathway. Some patients will be scheduled for primary surgery and are suitable for immediate recruitment to the study. Others will undergo neo-adjuvant chemotherapy prior to interval debulking surgery and may be recruited to the study at a later date, following chemotherapy. Potentially suitable patients will be provided with written information about the study at an outpatient clinic, followed up by a telephone call from the specialist or research nurse to answer questions and ascertain interest in the study. Written informed consent will be obtained at the surgical pre-assessment clinic by an appropriately trained member of the research team in line

For peer review only - http://bmjopen.bmj.com/site/about/guidelines.xhtml

with ICH Good Clinical Practice (GCP) guidelines. As part of the consent process, patients will be reminded that they are free to withdraw from the study at any time without giving a reason and without affecting their future treatment.

Inclusion criteria

Potential participants must satisfy the following criteria to be enrolled in the study:

- 18 years old or over
- Suspected or confirmed ovarian cancer (newly diagnosed) requiring cytoreductive surgery, whether primary or interval (following chemotherapy)
- CT scan evidence (with or without clinical evidence) compatible with FIGO stage III/IV ovarian cancer/primary peritoneal cancer at presentation (39) (Appendix 1)
- Eastern Cooperative Oncology Group (ECOG) Performance Status 0-1 (40)
- Willing to participate and able to give written informed consent

Exclusion criteria

Potential participants meeting any of the following criteria will be excluded from study participation:

- Diagnosis of concurrent malignancy
- Pregnant
- Haemoglobinopathies (e.g. sickle cell, thalassaemia)
- Unwilling to accept donor blood (e.g. on religious grounds)

Randomisation

Randomisation will be undertaken after written consent has been obtained, but as close to the start of surgery as possible; usually this will be on the morning of the operation day but if this is not possible for practical reasons, it may be performed earlier. Randomisation will be achieved by means of a web-based system created by the UK Clinical Research Collaboration (UKCRC) registered Peninsula Clinical Trials Unit (CTU) in conjunction with the trial statistician, using

For peer review only

For peer review only - http://bmjopen.bmj.com/site/about/guidelines.xhtml

random permuted blocks of varying size. Participants will be allocated to receive ICS reinfusion or donor blood transfusion in a 1:1 ratio, stratified by study site. To prevent any unnecessary delays in the operating theatre, cell salvage equipment will be set up in advance for all study participants, before confirmation of treatment allocation.

Trial interventions

Participants will be allocated to receive either donor blood transfusion or ICS reinfusion intraoperatively, in accordance with specified transfusion protocols. Donor blood will only be given (in standard volumes) when deemed necessary (e.g. after substantial blood loss and/or drop in haemoglobin) whereas ICS blood will be returned even if only small quantities are lost. Some participants may not require any intraoperative transfusion and some (in either arm of the trial) may require donor blood transfusion post-operatively.

Intraoperative cell salvage

All sites will follow a common ICS protocol and relevant site staff will undergo study-specific training prior to the study start. The make and model of ICS machine used in clinical practice varies across NHS Trusts and will not be standardised for this feasibility study. Collected blood will be processed via the ICS machine being used before being re-infused via a leucodepletion filter. Relevant data from a local intraoperative cell salvage audit form will be transcribed into the study-specific Case Report Form (CRF), including the amounts of salvaged blood processed and reinfused.

Donor transfusion

Participants allocated to donor transfusion will be considered for transfusion during surgery in accordance with clinical judgement, guided by local hospital policy. The factors triggering transfusion (e.g. excessive blood loss, hypotension, reduced Hb) will be documented in the CRF along with the amount and type of blood and blood products transfused.

Donor transfusion in ICS arm

For peer review only - http://bmjopen.bmj.com/site/about/guidelines.xhtml

Participants allocated to the ICS arm who need donor transfusion can be given donor blood at any time, during or after surgery, for the duration of their hospital stay. The factors triggering intraoperative donor transfusion in the ICS group will be documented in the CRF as well as the amount and type of any blood and blood products transfused.

Blinding

Surgeons, other theatre staff and the person recording details of intra-operative blood transfusion or reinfusion cannot be blinded in this study. The research nurse responsible for recording post-operative outcomes will aim to remain blinded to treatment allocation. Participants in either arm of the study may have some form of blood replacement in progress immediately post-surgery; it is unlikely that participants will be able to distinguish between the two types and either group may require donor blood for clinical reasons.

Feasibility outcomes

The outcomes for this study are the feasibility and acceptability of the study and study procedures in relation to recruitment, randomisation, intervention, blinding, participant retention and data completion. Both quantitative and qualitative methods will be used. Recruitment rate will be measured as the proportion of eligible patients who are subsequently enrolled, and the number of patients recruited per site per month. The number of patients screened, number/percent of patients approached, number/percent of patients excluded after screening/approach and the number/percent of patients providing consent will be assessed. Reasons for declining participation will be sought where possible, and the appropriateness and practicalities of the chosen eligibility criteria will be explored. The number/percent of women enrolled prior to initial surgery compared to following neo-adjuvant chemotherapy will be assessed. The timing of randomisation in relation to operation start will be recorded to assess the practicalities of randomising as late as possible, in particular what proportion are randomised on the day of surgery itself.

For peer review only - http://bmjopen.bmj.com/site/about/guidelines.xhtml

Use of ICS blood and donor blood will be recorded for both arms, partly to assess intervention fidelity but also to obtain an estimate of the proportion of people in the control arm that actually require donor blood. Reasons for non-use of ICS blood and/or use of donor blood in the ICS arm will be recorded.

Since the intervention takes place in the operating theatre it is unlikely that any participant will withdraw from intervention following randomisation. Attrition will be assessed by examining the number of participants lost to follow-up at any subsequent point in the study period. Reasons for discontinuation of follow-up will be sought from participants.

The success of blinding of allocation for participants and outcome assessors will be assessed by asking both the participant and research nurse to guess the allocation (including "unsure") at the 30 day post-operative follow-up and comparing the responses with the actual allocation.

Clinical outcomes

In the later, definitive trial, our primary outcome is likely to be either mortality or cancer recurrence, both of which are unlikely to occur in the time available in this feasibility study. Therefore, whilst readily accessible, these data will not be collected here. Other measures proposed for the later trial will be collected in this feasibility study at baseline and peri-operatively, with follow-up at 30 days and 6 weeks post-operatively. Participants recruited at an early stage of the study will also be followed up at 4.5, 7.5 and 10.5 months post-operatively as time allows (Figure 1). Clinical outcomes include:

- Inadvertent visceral injury (bladder, bowel, ureters, blood vessels, nerve)
- Return to theatre within 48 hours
- Surgical site infection (Appendix 2) within 30 days
- Thromboembolic complications (DVT, PE) within 30 days
- Number and nature of adverse events
- Amount of donor blood given (total and ≤24 hours post-surgery)

For peer review only - http://bmjopen.bmj.com/site/about/guidelines.xhtml

- Length of hospital stay
- Resource use
- Generic quality of life (QOL) measure: EQ-5D-5L
- Cancer-specific QOL measure: EORTC QLQ-C30 (Version 3.0) (confirmed cancer only)
- Ovarian cancer QOL measure: EORTC QLQ-OV28 (confirmed cancer only)

Data collection and management

Each participant will be allocated a unique trial number on consenting to the study and will be identified in all study-related documentation by her trial number and initials. A record of names and addresses linked to participants' trial numbers will be maintained by the research nurse at each site for administrative purposes, and stored securely.

~~Data collection~~

Data collected by the research team (Table 1) up to 30 days post-operatively will be recorded on study specific data collection forms (CRFs), usually by a research nurse. All data not routinely captured during the hospital admission but recorded straight into the CRF will be classified as source data. Participant self-completion questionnaires at baseline will be completed during a face-to-face meeting with a research nurse, following written informed consent. The research nurse will return completed CRFs and baseline questionnaires to the CTU. Subsequent self-completion questionnaires (6 weeks post-operatively and three monthly thereafter as time allows) will be mailed to participants directly from the CTU and returned by participants to the CTU in a pre-paid envelope provided. In the event of non-return of a questionnaire, a reminder will be sent from the CTU in the first instance. If there is no response from the two mailings, the CTU will inform the local research nurse who will telephone the participant in order to encourage compliance with follow-up.

For peer review only

For peer review only - http://bmjopen.bmj.com/site/about/guidelines.xhtml

Table 1: Trial schedule

	Pre-operative		OPERATION and peri-operative data collection	Post-operative follow-up				
	Screen	Baseline		1	2	3†	4†	5†
				30 days post-op	6 weeks post-op	3 months after follow-up 2	6 months after follow-up 2	9 months after follow-up 2
Screen/eligibility	x							
Consent		x						
Demographics & history		x						
Randomisation		x						
EORTC QLQ-C30*		x			x	x	x	x
EORTC QLQ-OV28*		x			x	x	x	x
EQ-5D-5L		x			x	x	x	x
Adverse events				x				
Resource use questionnaire		x			x	x		
Qualitative interviews					x	x		

All data management procedures will be conducted in line with written standard operating procedures and study-specific work instructions. Data will be collected and stored in accordance with the Data Protection Act, 1998. Completed paper CRF's will be posted to the CTU at agreed time points for double-data entry on to a password-protected database, with copies retained at the relevant study site. Forms will be tracked using a web-based trial management system. Double-entered data will be compared for discrepancies using a stored procedure and discrepant data will be verified using the original paper data sheets. Pseudo-anonymised paper-based study data will be stored in locked filing cabinets within a locked office within the CTU. Electronic records will be stored in a SQL server database, stored on a restricted access, secure server maintained by Plymouth University. Direct access to the trial data will be restricted to members of the research team and the CTU, with access granted to the Sponsor on request.

Losses to follow-up

If a participant does not wish to complete follow-up, regardless of whether she has received trial treatment or not, she will be formally withdrawn from the study. Participants will be asked to explain their reason for withdrawing from follow-up, but are under no obligation to do so. The

For peer review only

appropriate study-specific withdrawal form should be completed and sent to the CTU as soon as possible as official notification of the withdrawal. Withdrawal from follow-up and the reason, if known, should also be clearly documented in the participant's clinical records.

Participants who do not require blood transfusion or who discontinue follow-up for any reason will not be replaced within the study. Data collected prior to withdrawal will be included in the study analysis unless a participant specifically requests that her data are removed from the database.

Adverse events

Any serious adverse event occurring from the start of the participant's operation until the date the participant completes follow-up (or withdraws from the study), whether thought to be related to trial intervention or not, must be reported to the CTU within 24 hours of the research team becoming aware of it. Non-serious adverse events considered to be related to the surgery for this cancer episode and occurring from the start of the participant's operation until 30 days post-operatively will be captured by the research nurse during the participant's hospitalisation and via the 30-day telephone follow-up call to each participant. The PI is responsible for assessing whether or not an adverse event is related to the trial intervention. The TMG and TSC will regularly review (S)AE data.

Trial management and oversight

The trial management group (TMG) including the CI, CTU trial manager, trial statistician and other relevant personnel (e.g. other clinical colleagues, CTU data manager and patient representatives) will meet regularly (usually monthly) throughout the duration of the trial to monitor progress, resolve day-to-day problems, oversee development of documentation and forms, monitor participant recruitment and follow-up, review the budget, discuss analysis, results, draft reports and dissemination.

The Trial Steering Committee (TSC) will oversee the conduct and safety of the trial in accordance with the terms of an agreed TSC Charter, ensuring that milestones are achieved and general scientific probity is maintained. The independent TSC chair will be Professor Henry Kitchener.

For peer review only - http://bmjopen.bmj.com/site/about/guidelines.xhtml

Other committee members will include an independent statistician, independent consultant gynaecological oncology surgeon, independent consultant anaesthetist, a lay representative and the Chief Investigator. The trial statistician, trial manager(s) and Sponsor representative will be invited to attend TSC meetings as observers. The TSC will meet once before the start of the study and approximately three more times during the study as agreed by the Committee itself. Given that this is a feasibility study with no interim analysis and no serious safety concerns, an independent Data Monitoring Committee will not be convened.

Statistical considerations

Sample size for a feasibility study is necessarily a compromise between the twin assets of precision and efficiency. For any binary "outcome" our target sample size of 60 will result in a 95% confidence interval of no greater than about +/-12 percentage points, while in a single arm the target of 30 will have a CI of no more than +/- 17 percentage points.

Data analysis will enable the feasibility outcomes to be addressed in order to inform a decision about proceeding to a definitive trial. Data will be presented in accordance with the extension to the ConSORT statement for pilot and feasibility studies. They will detail the numbers of patients that were approached, the number that were eligible and the number providing consent. Likewise, compliance rates at all stages will be presented, including the numbers of questionnaires completed at each stage and more generally the completeness of data on all outcomes at each time point. Participating patients' characteristics (demographics, comorbidities, clinical details) will be summarised and, where possible, compared with the overall population of relevant patients to explore possible factors associated with participation. Where possible, the reasons will be ascertained for potentially eligible patients not being approached to consider participation.

Descriptive data on the clinical outcomes will be presented by trial arm, using appropriate measures of central tendency and variation for continuous measures and numbers/percentages for categorical

For peer review only - http://bmjopen.bmj.com/site/about/guidelines.xhtml

measures. All planned analyses will be described in a written statistical analysis plan. No formal statistical tests will be conducted.

Qualitative study

A qualitative evaluation will assess the acceptability of the intervention to women taking part in the study, in particular attitudes towards reinfusion of salvaged blood and transfusion of donor blood. The study will also gain an understanding of the women's experience of taking part in the research processes of the TIC TOC study and what influenced their decision to take part. Following surgery, up to 20 women from across all centres will be asked to take part in individual face to face or telephone semi-structured interviews using a topic guide that has been developed with patient and public involvement (PPI) involvement (Appendix 3). Purposive sampling techniques will ensure a range of women are selected according to centre, education, age, ethnicity, socioeconomic status, and social support.

As the trial schedule allows, the same women will be approached to take part in a brief telephone interview three months after the first interview. The purpose of the second interview is to determine participants' perceptions about the follow-up research processes and ask their opinion about whether anything should change in a full trial. Surgeons from each centre will also be invited to participate in one brief telephone interview each to understand the issues considered in deciding whether to offer women the opportunity to take part in the study.

The qualitative data will be managed using computer software such as Nvivo 11 and thematically analysed (41, 42). The researcher will ensure accuracy of the transcription and read the transcript several times to become immersed in the data, noting initial thoughts and ideas. Codes will be assigned to extracts of the data relevant to the project. Codes with similar meaning will be grouped together in themes. Using constant comparison techniques across the transcripts' themes looking for similarities and differences, the themes will be reviewed and refined. Extracts from the data will be used in the final report. Reflexive research memos will be used as an audit trail of the analysis

For peer review only - http://bmjopen.bmj.com/site/about/guidelines.xhtml

procedure (43). A second qualitative researcher will conduct an independent analysis of a subset of six transcripts before the researchers meet to discuss and agree the findings. Findings will also be presented to the study's patient advisory group for discussion. Any significant differences of opinion will be discussed with the Chief Investigator. A model may be developed to explain the factors affecting recruitment and retention to the trial to inform development of the research processes required in any future full trial.

Economic data and analyses

A definitive study will include a within trial economic evaluation to compare costs and health outcomes of ICS versus donor blood within the time frame of the study and a decision analytic model to extrapolate any future health benefits and costs to the lifetime of the participant. The evaluations will primarily be in relation to quality adjusted life years and will take a health and social perspective on costs, in accordance with NICE guidelines (37). Secondary analyses will take place in relation to important clinical outcomes of interest for the definitive trial such as deaths averted and disease-free progression. This study aims to test the feasibility of collecting enough resource use and outcome data to perform the future economic evaluations.

Data collection tools will be prepared and refined with a view to undertaking the two planned economic evaluations within the future study. These evaluations will take on a health and social care payer perspective. Should participant-reported resource use data allow, the future within-trial economic evaluation will take on a societal perspective on costs in secondary analyses, to further capture the burden to participants, carers, and society. The parameters for the lifetime economic decision model (costs, outcomes, and probabilities of outcomes to occur) will be informed by the within trial economic results. If feasible, costs from a societal perspective may be included in the lifetime economic decision model as well.

Resources will be collected from several sources. In the immediate post-operative period, research nurses will record resources pertaining to the participant's surgery and subsequent hospital stay.

For peer review only

For peer review only - http://bmjopen.bmj.com/site/about/guidelines.xhtml

Where possible, research staff will also review participants' medical notes at 4.5 months post-operatively to collect hospital contacts following initial discharge (i.e. re-hospitalisations, outpatient and emergency visits). Participant-completed resource use questionnaires will be administered at both six weeks and 4.5 months post-operatively (where the trial schedule allows) to collect other resources used. These questionnaires will be delivered by post and include questions related to in-patient and out-patient hospital visits; community--based services such as General Practice doctor and nurse contacts, physiotherapy, occupational therapy and other community contacts; use of personal social services such as home care workers and social workers; privately paid therapies and expenses; time off work and lost leisure; and informal care required from family and friends. Completion rates, missing data and the method of administering questionnaires will be reviewed to identify potential problems with data collection methods and to seek solutions to minimise participant/staff burden if required. We will report frequency, mean, and standard deviation of resources used by trial arm to explore potential cost-drivers for the main study.

The EuroQoL EQ-5D questionnaire will capture generic quality of life differences between the trial arms. In a recent study of EQ-5D valuation sets, the 3L and 5L versions of the EQ-5D produced substantially different estimates for cost-effectiveness (44) and prompted NICE to issue a position statement in August 2017 to recommend the future use of the 3L version (45). In this study, we will use the mapped utility scores from the 3L to the 5L version using the Van Hout algorithm (46) for the UK population, as recommended by the NICE statement. We expect to use the 3L version in the future study and not proceed with the study of the distribution properties produced by the 5L version scores in this feasibility study.

Patient and Public Involvement and Engagement

The study has benefitted from its inception from an enthusiastic patient advisory group. The aim of PPI in the study is to ensure that the trial is equitable and acceptable to the women taking part by embedding the women's experiential expertise of cancer throughout the trial design and processes. The group comprises six women aged between 50-80 years, who have experienced a cancer

For peer review only

diagnosis and are living in Cornwall. However, one member is formerly from Gateshead, where she was treated for her cancer, so is able to bring her experience of the patient pathway to inform the trial processes across the sites. Another member and co-applicant is the founder of PANTS cancer charity in Cornwall.

The PPI work is undertaken using a predominately collaborative approach with engagement functions embedded within it. The members worked with the research team on the research design and in particular the patient approach, providing input into the grant application, language, content and layout of the participant documentation. The group have worked on the qualitative interview topic guide content and are also working with the qualitative researchers on analysis of the participant interview transcripts. The members are fully integrated into the team and regularly attend the trial management meetings, as well as providing advice and suggesting solutions to problems encountered during the trial.

The members will attend patient and public events and conferences to engage with other members of the public and professionals and share their experience of supporting and being part of the design and management of research. They will also work together with the wider research team to prepare a lay summary of the findings and on other communications such as website, Twitter and Facebook articles.

All members of the research team contribute to the training and support of the PPI members. The mechanisms to achieve these are multifactorial and include specific discussion around methodology and trial processes in PPI meetings, explaining the terminology in lay language, providing information, such as the Involve jargon buster sheet and conducting workshops for specific tasks (e.g. poster development), as well as signposting to other resources such as the Involve website.

Data sharing

For peer review only - http://bmjopen.bmj.com/site/about/guidelines.xhtml

After the end of the study, pseudonymised information collected during the study may be made available to other researchers under an appropriate data sharing agreement; it will not be possible to identify participants personally from any information shared.

Indemnity

This is an NHS-sponsored research trial. If an individual suffers negligent harm as a result of participating in the trial, NHS indemnity covers NHS staff and those people responsible for conducting the trial who have honorary contracts with the relevant NHS Trust.

ETHICS AND DISSEMINATION

The results of this feasibility study will be published in peer-reviewed journals and presented at relevant national/international conferences and to patient groups. Participants of the trial will be sent a summary of the findings and these will also be disseminated via the pantscancer.org charity, Target Ovarian Cancer charity and participating NHS Trusts' websites.

DISCUSSION

Research has shown that donor blood transfusions have been associated with poorer outcomes including increased mortality, wound, pulmonary and renal complications (8); this has been ascribed to transfusion-induced immune modulation (TRIM) (10) which is a transient depression of the immune system following transfusion with blood products. The Cochrane meta-analysis of randomised trials estimated perioperative allogeneic blood transfusion to be associated with increased risk of recurrence with odds ratio of 1.42 (95% CI, 1.20 to 1.67) in surgery for colorectal cancer (47). Long-term results from a clinical trial suggest that this effect of allogeneic blood transfusion is persistent (48, 49). This led to the suggestion of introducing measures that would help limit the use of allogeneic blood transfusion (13).

For peer review only - http://bmjopen.bmj.com/site/about/guidelines.xhtml

Patient blood management is an evidence-based patient-tailored approach aimed at reducing the need for allogeneic blood transfusion by managing anaemia, perioperative blood conservation, surgical haemostasis, and drug use (50). Perioperative blood conservation measures include interventions such as the administration of agents to diminish blood loss (e.g. tranexamic acid, fibrin sealant), agents that promote red blood cell production (e.g. erythropoietin) and techniques for reinfusing a patient's own blood including cell salvage (29). Previous randomised and non-randomised studies have provided evidence that the use of intraoperative cell salvage can reduce the need for allogeneic blood transfusion (ABT) (10). A systematic review of 75 randomised trials highlighted that salvaged blood reinfusion reduced the rate of exposure to ABT by 38% (relative risk, 0.62; 95% confidence interval [95% CI], 0.55-0.70) (51). However, concern exists that blood collected by intraoperative cell salvage might result in reinfusion of tumour cells and subsequent distant metastases thus limiting the use of cell salvage across oncological specialties. However, in patients undergoing surgery for a gynaecological malignancy, the use of a leucocyte depletion filter was shown to be effective in eliminating viable nucleated malignant cells from the returned blood during collection, processing, and leukofiltration (28). Similarly, in vitro work shows that depletion filters are highly efficient at removing malignant cells, leading to removal rates of between 80 and 100% (26, 27).

Patients with primary or metastatic cancer are known to have CTCs in the blood. The concentration of CTCs varies widely depending on tumour type and stage of disease (34). There is evidence from a range of different cancer surgeries that operative manipulation of tumour during surgery leads to peripheral blood concentrations of malignant cells many times higher than could be attained with cell salvage alone (34, 35, 52).

There is emerging evidence suggesting that far from compromising outcomes, intraoperative autologous transfusion is associated with improved outcomes in surgery for other gynaecological cancers such as cervical cancer (31). Several studies in early stage (I-IIA) cervical cancer patients report that intraoperative autologous transfusion significantly reduces the need for donor blood

For peer review only - http://bmjopen.bmj.com/site/about/guidelines.xhtml

transfusion, without compromising survival or post-operative complication rates (31). In addition, no distant recurrences have been reported (31). However, most of the evidence on the use of salvaged blood in cancer surgery is based on retrospective and observational studies. These studies are insufficient to draw any definitive conclusions regarding adverse events related to a particular intervention in the presence of multiple confounding factors. Therefore in order to mitigate for confounding factors a large well-designed randomised controlled trials are required (53). Our trial provides new evidence in the use of cell salvage in ovarian cancer surgery and will add to a more general evidence base informing the use of ICS in other areas, in particular other cancers.

~~**Conflict of interest statement**~~

~~The authors declare no conflict of interest.~~

~~This work was supported by the National Institute for Health Research (NIHR) Research for Patient Benefit (RfPB) programme, grant number PB-PG-1014-35005.~~

Contributorship statement

All authors except NF and JP were co-applicants on the NIHR RfPB grant application and as such were involved in the design of this feasibility study. All authors contributed to successive drafts of this paper.

KG is the Chief Investigator, provided clinical expertise and was responsible for conception and design of the study as well as drafting and revising of the article.

NF was responsible for the first draft of this paper.

CP contributed to study design.

AB contributed to study design and trial management.

PE is the trial statistician and provided expertise in the overall design of the trial.

JF provided expertise in cell salvage and drafted the cell salvage protocol.

AL provided clinical expertise and helped with design of the study.

EM was responsible for the design and analysis of the economic evaluation component.

For peer review only - http://bmjopen.bmj.com/site/about/guidelines.xhtml

JP contributed to study design and coordinated the PPI input.

CR provided anaesthetics and cell salvage expertise

JV is the trial manager, responsible for overseeing the day-to-day running of the trial.

JW was responsible for the design and conduct of the qualitative study.

Representatives of the study sponsor reviewed the study protocol.

Competing ~~nflict of~~ interest ~~statement~~

The authors declare no competing~~nflict of~~ interests.

For peer review only

Funding

This work was supported by the National Institute for Health Research (NIHR) Research for Patient Benefit (RfPB) programme, grant number PB-PG-1014-35005. The funder was not involved in the design, conduct or analysis of the study.

Data sharing

After the end of the study, pseudonymised information collected during the study may be made available to other researchers under an appropriate data sharing agreement.

Acknowledgements

The authors are grateful for the support of the study sponsor (Research, Development and Innovation Department, Royal Cornwall Hospitals NHS Trust, Treliske, Truro TR1 3LJ) and the South West Peninsula NIHR Clinical Research Network. We are also indebted to the members of our PPI group for their continued support of the trial. In addition, the authors would like to thank

For peer review only - http://bmjopen.bmj.com/site/about/guidelines.xhtml

the following members of the TIC TOC trial team: Mr S Chatopadhyay, Mr G Hughes, Mr R Naik and Dr P Ricketts.

DH disclaimer

The views expressed are those of the author(s) and not necessarily those of the NHS, the NIHR or the Department of Health.

For peer review only

References

1. Cancer Research UK. Ovarian cancer incidence statistics http://www.cancerresearchuk.org/cancer-; 2014.
2. National Cancer Intelligence Network (NCIN). Overview of Ovarian Cancer in England: Incidence, Mortality and Survival. Trent Cancer Registry November 2012 [Internet]. 2012.
3. Schwartz PE. Cytoreductive surgery in the management of ovarian cancer Oncology 22: 1025-33. 2008;22(9):1025-33 .
4. Hoskins WJ MW, Brady MF, et al. The effect of diameter of largest residual disease on survival after primary cytoreductive surgery in patients with suboptimal residual epithelial ovarian carcinoma. Am J Obstet Gynecol 1994;170(4):974-9.
5. Peiretti M ZV, Aletti GD, Bocciolone L, Colombo N, Landoni F, Minig L, Biffi R, Radice D, Maggioni A. Role of maximal primary cytoreductive surgery in patients with advanced epithelial ovarian and tubal cancer: Surgical and oncological outcomes. Single institution experience. Gynecol Oncol. 2010;119(2):256-64.
6. Prescott LS AT, Brown AJ, Taylor JS, Munsell MF, Sun CC, et al,. Perioperative blood transfusion in gynecologic oncology surgery: analysis of the National Surgical Quality Improvement Program Database. Gynecologic oncology. 2015;136(1):65-70.
7. Warner LL DS, Martin JR, Lemens MA, McGree ME, Weaver AL, et al,. The impact of perioperative packed red blood cell transfusion on survival in epithelial ovarian cancer. . Int J Gynecol Cancer 2013;23:1612–9.
8. Ferraris VA DD, Saha SP, Austin PC, Zwischenberger JB. Surgical Outcomes and Transfusion of Minimal Amounts of Blood in the Operating Room. JAMA Surgery. Jan 2012, Vol 147, No. 1 JAMA Surgery. 2012;147(1):49-55.
9. PHB Bolton-Maggs (Ed) DP, A Watt, D Thomas and H Cohen on behalf of the Serious Hazards of Transfusion (SHOT) Steering Group. The 2012 Annual SHOT Report (2013)
10. Blajchman MA, and Eleftherios C. Vamvakas. "Transfusion-Related Immunomodulation." Practical Transfusion Medicine. Third Edition (2009): 98-106. 2009.
11. Goubran H SD, Radosevic J, Burnouf T, Seghatchian J,. Transfusion-related immunomodulation and cancer. Transfus Apher Sci. 2017;56(3):336–40.
12. Ashworth A KAA. Cell salvage as part of a blood conservation strategy in anaesthesia. Br J Anaesth. 2010;105(4):401-16.
13. The Association of Anaesthetists of Great Britain and Ireland (AAGBI). The Association of Anaesthetists of Great Britain and Ireland (AAGBI) Safety Guideline—Blood Transfusion and the Anaesthetist: Intra-operative Cell Salvage2009.
14. Krajewski K AR, Pung N, Wald S, Lazareff J, Kawamoto HK, Bradley JP. Successful blood conservation during craniosynostotic correction with dual therapy using Procrit and cell saver. J Caraniofac Surg. 2008;19:101-5.
15. Bridgens JP EC, Dobson PMS, Hamer AJ. Intraoperative red blood-cell salvage in revision hip surgery. J Bone Joint Surg 2008;89:270-5.
16. Colwell CW Jr. BE, West C, Hardwick ME, Morris BA. Erythrocyte viability in blood salvaged during total joint arthroplasty with cement. J Bone Joint Surg 2002;84:23-5.
17. Williamson KR TH. Intraoperative blood salvage: a review. Transfusion. 1991;31(7):662-75.
18. Akbulut S KC, Yilmaz M, Ince V, Ozgor D, Karabulut K, Eris C, Toprak HI, Aydin C, Yilmaz S. Effect of autotransfusion system on tumor recurrence and survival in hepatocellular carcinoma patients World J Gastroenterol. 2013;19(10):1625–31.
19. Wang G BD, Martin J, Cheng D The efficacy of an intraoperative cell saver during cardiac surgery: a meta-analysis of randomized trials. Anesth Analg. 2009;109(2):320-30.

For peer review only

20. Marković M DL, Savić N, Sindjelić R, Ille T, Dragas M. Intraoperative cell salvage versus allogeneic transfusion during abdominal aortic surgery: clinical and financial outcomes. Vascular. 2009;17(2):83-92.
21. Breakwell LM GC, Dobson P. T,. The efficacy of autologous blood transfusion in bilateral total knee arthroplasty. Knee. 2000;7(3):145–7.
22. DeAndrade D WJ, Triulzi DJ, Alarcon L, Wisniewski MK, Dyga R, et al,. Very low rate of patient-related adverse events associated with the use of intraoperative cell salvage. Transfusion. 2016 Nov;56(11):2768–72. Transfusion. 2016;56(11):2768–72.
23. Hansen E WN, Kneuchel R, Ruschoff J, Hofstaedter F. Hansen E, Wolff N, Kneuchel R, Ruschoff J, Hofstaedter F. Tumor calls in blood shed from the surgical field. Arch.Surg. 1995; 130(4) 387-393. Tumor calls in blood shed from the surgical field. 1995;130(4):387-93.
24. Kumar N ZA, Kantharajanna SB, Khoo BL, Lim CT, Thiery JP,. Metastatic efficiency of tumour cells can be impaired by intraoperative cell salvage process: truth or conjecture? . Transfus Med. 2017;Suppl 5:327-34.
25. Motoyama S SR, Kamata S, Kitamura M, Okuyama M, Imano H, Nakamura M, Suzuki H, Omokawa S, Motohashi Y, Ogawa J. Survival advantage of using autologous blood transfusion during surgery for esophageal cancer. Surg Today. 2002;32(11):951-8.
26. Karczewski DM LM, Glaves D. The efficiency of an autotransfusion system for tumor cell removal from blood salvaged during cancer surgery. Anesth Analg 1994;78(6):1131-5.
27. Fruhauf NR DO, Kaudel CP, Kasimir-Bauer S, Oldhafer KJ. Filtration of malignant cells: tumour cell depletion in an ex vivo model using a leukocyte adhesion filter. Perfusion 2001;16:Suppl:51-5.
28. Catling S WS, Freites O, Rees M, Davies C, Hopkins L. Use of a leucocyte filter to remove tumour cells from intra-operative cell salvage blood. Anaesthesia 2008;63(12):1332-8.
29. Zaw AS BKS, Kumar N,. Is Autologous Salvaged Blood a Viable Option for Patient Blood Management in Oncologic Surgery? . Transfusion Medicine Reviews. 2017;31(1):56-61.
30. Connor JP MP, Alagoz T, Anderson B, Bottles K, Buller RE. Intraoperative autologous blood collection and autotransfusion in the surgical management of early cancers of the uterine cervix. Obstet Gynecol. 1995;86(3):373- 8.
31. Mirhashemi R AH, Deepika K, Estape R, Angioli R, Martin J, Rodriguez M, Penalver MA. The impact of intraoperative autologous blood transfusion during type III radical hysterectomy for early-stage cervical cancer. Am J Obstet Gynecol 1999;181(6):1310-5; discussion 5-6.
32. Ford BS SS, Rezaishiraz H, Huben RS, Mohler JL,. Effect of perioperative blood transfusion on prostate cancer recurrence. Urol Oncol. 2008;26(4):364-7.
33. Heal JM CC, Blumberg N. Perioperative blood transfusions and prostate cancer recurrence and survival Am J Surg 1988;156(5):374–9
34. AllardWJ MJ, MillerMC, Repollet M, Connelly MC, Rao C, et al,. Tumor cells circulate in the peripheral blood of all major carcinomas but not in healthy subjects or patients with nonmalignant diseases. Clin Cancer Res. 2004;10:6897–904.
35. Oefelein MG KK, Herz B, Blum MD, Holland JM, Keeler TC, Cook WA, Ignatoff JM. Molecular detection of prostate epithelial cells from the surgical field and peripheral circulation during radical prostatectomy. J Urol 1996;155(1):238-42.
36. N Kumar ASZ, B.L. Khoo, S. Saminathan, Z. Jiang, G. Singh, C.T. Lim, B.L. Thiery,. Intraoperative cell salvage in metastatic spine tumour surgery reduces potential for reinfusion of viable cancer cells. EurSpine J. 2016;25(12):4008-15.
37. Allan AL KM. Circulating tumor cell analysis: technical and statistical considerations

for application to the clinic. J Oncol 2010;2010:426218.

For peer review only - http://bmjopen.bmj.com/site/about/guidelines.xhtml

38. Galaal K BA, Smits A, Lopes AD,. Intraoperative Cell Salvage in Surgery for Ovarian Cancer: Time for Action. . Medical Journal of Obstetrics and Gynecology 2014;2(2):1035.
39. Society of Gynecologic Oncology. FIGO ovarian cancer staging: https://www.sgo.org/wp-content/uploads/2012/FIGO-Ovarian-Cancer-Staging_1.10.14.pdf; 2014 [
40. Oken MM, Creech, R.H., Tormey, D.C., Horton, J., Davis, T.E., McFadden, E.T., Carbone, P.P. Toxicity And Response Criteria Of The Eastern Cooperative Oncology Group. Am J Clin Oncol 1982;5:649-55.
41. Braun V CV. Using thematic analysis in psychology. Qualitative Research in Psychology. 2006;3:77-101.
42. Green J TN. 2004. Qualitative Health Methods for Health Research. London: Sage Publications.
43. Liamputtong P ED. Qualitative Research Methods. Second Edition ed. Oxford: Oxford University Press; 2005. In: Press OU, editor. Oxford:2005.
44. Wailoo A HAM, Grimm S et al. Comparing the EQ-5D-3L and 5L versions. What are the implications for cost effectiveness estimates? NICE Decision Support Unit report 2017 6th July 2017.
45. https://www.nice.org.uk/Media/Default/About/what-we-do/NICE-guidance/NICE-technology-appraisal-guidance/eq5d5l_nice_position_statement.pdf.
46. Van Hout B JM, Feng Y et al,. Interim scoring for the EQ-5D-5L: Mapping the EQ-5D-5L to EQ-5D-3L value sets. Value in Health. 2012;15:708-15.
47. Amato A PM. Perioperative blood transfusions and recurrence of colorectal cancer. Cochrane Database of Systematic Reviews 2006, Issue 1. Art. No.: CD005033. DOI: 10.1002/14651858.CD005033.pub2. Cochrane Database of Systematic Reviews. 2006(1).
48. Linder BJ FI, Cheville JC, Tollefson MK, Thompson RH, Tarrell RF, Thapa P, Boorjian SA. The Impact of Perioperative Blood Transfusion on Cancer Recurrence and Survival Following Radical Cystectomy Eur Urol. 2013;63(5):839–45
49. Harlaar JJ GM, Hop WC, Lange JF, Busch OR, Jeekel H Blood Transfusions and Prognosis in Colorectal Cancer: Long-term Results of a Randomized Controlled Trial Ann Surg. 2012;256:681-6
50. Spahn DR SA, Hofmann A,. The chiasm: transfusion practice versus patient blood management. Best Pract Res Clin Anaesthesiol 2013;27:37-42.
51. Carless PA HD, Moxey AJ, O'Connell D, Brown T, Fergusson DA. Cell salvage for minimising perioperative allogeneic blood transfusion. Cochrane Database of Systematic Reviews 2010(4):Art. No: CD001888.
52. Yamaguchi K TY, Aoki S, FutamuraM, Saji S,. Significant detection of circulating cancer cells in the blood by reverse transcriptase-polymerase chain reaction during colorectal cancer resection. Ann Surg 2000;232:58-65.
53. Yazer MH TD. Things aren't always as they seem: what the randomized trialsof red blood cell transfusion tell us about adverse outcomes. Transfusion 2014;54:3243–6 [quiz 2].

For peer review only - http://bmjopen.bmj.com/site/about/guidelines.xhtml

Legend

Table 1 Trial schedule

Figure 1 Summary of trial design

Appendix 1 FIGO Ovarian Cancer Staging

Appendix 2 Definition of surgical site infection

Appendix 3 Topic guide for participant interviews

Appendix 4 Participant Information Sheet

Appendix 5 Informed Consent Form

For peer review only - http://bmjopen.bmj.com/site/about/guidelines.xhtml

TABLES AND FIGURES

Table 1: Trial schedule

	Pre-operative		OPERATION and peri-operative data collection	Post-operative follow-up				
	Screen	Baseline		1	2	3†	4†	5†
				30 days post-op	6 weeks post-op	3 months after follow-up 2	6 months after follow-up 2	9 months after follow-up 2
Screen/eligibility	x							
Consent		x						
Demographics & history		x						
Randomisation		x						
EORTC QLQ-C30*		x			x	x	x	x
EORTC QLQ-OV28*		x			x	x	x	x
EQ-5D-5L		x			x	x	x	x
Adverse events				x				
Resource use questionnaire		x			x	x		
Qualitative interviews					x	x		

† As time allows
*Confirmed cancer participants only for post-operative follow-up

297x420mm (300 x 300 DPI)

Figure 1: Summary of trial design

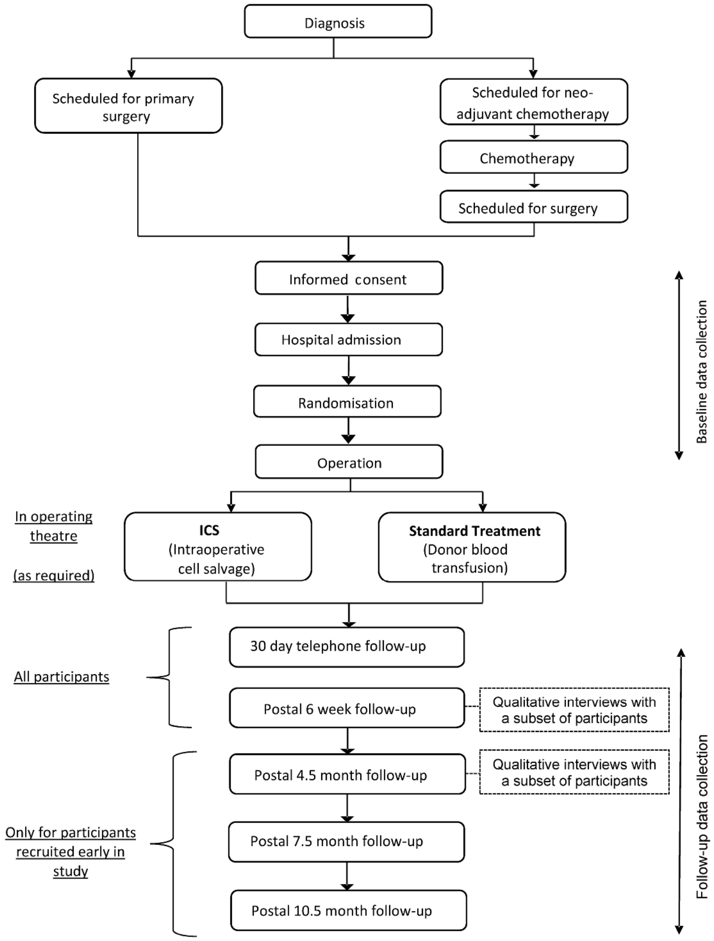

TIC TOC protocol paper Table_figure_K Galaal_31.07.18 ISRCTN19517317 Page **2** of **2**

297x420mm (300 x 300 DPI)

Appendix 1: FIGO Ovarian Cancer Staging

Effective 1 January 2014

**STAGE I: Tumour confined to ovaries**	
**IA**	Tumour limited to one ovary, capsule intact, no tumour on surface, negative washings
**IB**	Tumour involves both ovaries, otherwise like IA
**IC**	Tumour limited to one or both ovaries
IC1	Surgical spill
IC2	Capsule rupture before surgery or tumour on ovarian surface
IC3	Malignant cells in the ascites or peritoneal washings

**STAGE II: Tumour involves one or both ovaries with pelvic extension (below the pelvic brim) or primary peritoneal cancer**	
**IIA**	Extension and/or implant on uterus and/or Fallopian tubes
**IIB**	Extension to other pelvic intraperitoneal tissues

**STAGE III: Tumour involves 1 or both ovaries with cytologically or histologically confirmed spread to the peritoneum outside the pelvis and/or metastasis to the retroperitoneal lymph nodes**		
**IIIA**	Positive retroperitoneal lymph nodes and/or microscopic metastasis beyond the pelvis	
IIIA1	Positive retroperitoneal lymph nodes only	
	IIIA1 (i)	Metastasis ≤ 10mm
	IIIA1 (ii)	Metastasis > 10mm
IIIA2	Microscopic, extrapelvic (above the brim) peritoneal involvement ± positive retroperitoneal lymph nodes	
IIIB	Macroscopic, extrapelvic, peritoneal metastasis ≤ 2cm ± positive retroperitoneal lymph nodes. Includes extension to capsule of liver/spleen	
IIIC	Macroscopic, extrapelvic, peritoneal metastasis > 2cm ± positive retroperitoneal lymph nodes. Includes extension to capsule of liver/spleen	

**STAGE IV: Distant metastasis excluding peritoneal metastasis**	
IVA	Pleural effusion with positive cytology
IVB	Hepatic and/or splenic parenchymal metastasis, metastasis to extra-abdominal organs (including inguinal lymph nodes and lymph nodes outside of the abdominal cavity)

Other major recommendations are as follows:

- Histologic type including grading should be designated at staging
- Primary site (ovary, Fallopian tube or peritoneum) should be designated where possible
- Tumours that may otherwise qualify for stage I but involved with dense adhesions justify upgrading to stage II if tumour cells are histologically proven to be present in the adhesions.

For peer review only

For peer review only - http://bmjopen.bmj.com/site/about/guidelines.xhtml

Appendix 2: Definition of surgical site infection

For the purposes of this study, surgical site infection (48, 49) is defined as an infection that:-

i) occurs within 30 days after the operation and

ii) appears to be related to the operation and

iii) involves deep soft tissues (e.g. fascial and muscle layers) of the incision and at least one of the following:-

a) Purulent drainage from the deep incision but not from the organ/space component of the surgical site

b) A deep incision spontaneously dehisces or is deliberately opened by a surgeon when the patient has at least one of the following signs or symptoms: fever (> 38 C), localized pain, or tenderness, unless site is culture-negative.

c) An abscess or other evidence of infection involving the deep incision is found on direct examination, during reoperation, or by histopathologic or radiologic examination.

d) Diagnosis of a deep incision SSI by a surgeon or attending physician

For peer review only

For peer review only - http://bmjopen.bmj.com/site/about/guidelines.xhtml

Appendix 3: Topic guide for participant interviews

First qualitative interview (6 weeks)

Topic	Questions	Prompts
**Opening question**	How are you feeling after your operation? Tell me a bit about yourself?	Role in life – past or present employment<br>Family<br>Be sensitive and understanding
**Recruitment**	How were you approached to take part in the TIC TOC study?<br><br>What did you think about the way the study was introduced?<br><br>What did you understand about the study?<br><br>What questions did you have? Did you receive answers you understood?	Which member of staff, how approached(surgeon, specialist nurse)
**Specific understanding**	What did you understand about reintroducing your own blood?<br><br>What did you understand by donor blood transfusion?	Which method did you think was safest?
**Involvement of family and friends**	Did you ask anyone else for their opinions?<br><br>If yes, who were they?<br>What was their opinion?	Explore any negative responses from family and friends<br>Explore any positive responses from family and friends
**Decision process**	What things did you think about when deciding if you were going to take part?	Barriers<br>Factors that stopped the woman taking part (fear, overwhelmed by potential cancer diagnosis, chance would get cell salvage anyway (some sites), lack of understanding, unable to read research literature)<br>Facilitators<br>Factors that encouraged her to take part (trust of surgeon, research staff, feeling obligated, fear, distrust of donated blood or salvaged blood)
**Research processes**	When you came to the first clinic to see your consultant, how were you treated in the research part of your appointment?<br><br>Tell me what you felt about the specialist nurse asking you if you wanted to take part in the TIC TOC study?<br>What did you think about the timing of being recruited to the study?<br><br>What did you think about the questionnaires?	Check woman's talk is about the research.<br><br>Woman may want to talk about their cancer experience – allow it.<br><br>Baseline questionnaires only

For peer review only - http://bmjopen.bmj.com/site/about/guidelines.xhtml

**Allocation**	Which group do you think you were allocated?<br><br>Why?	Do not say which
**Information about next appointments**	As part of your normal care, you will be followed up by your consultant or his/her team. As part of the research study you will receive some further postal questionnaires.<br><br>Can I contact you again in about 6 months to see what you think about the postal follow-up?	

For peer review only

Second qualitative interview (three months after first, by telephone)

**Topic**	**Questions**	**Prompts**
**Opening question**	Since we last spoke, how have you been getting on?<br><br>I have a few questions to ask you about your experience of taking part in the TIC TOC study.	May not be feeling well.<br><br>May be on chemotherapy treatment.<br><br>Be sensitive and understanding
**Research process: follow-up questionnaires**	Where did you complete your questionnaires?<br><br>Did you have help to complete the questionnaires?<br><br>What did you like about the telephone/postal follow up?<br><br>What didn't you like about the telephone/postal follow up?<br><br>Was there anything that could be improved?<br><br>Did you know who to contact if you did not wish to keep taking part?<br><br>What did you think about the questionnaires asking you what health services you had used?	Did the woman know how to make a complaint?<br><br>(probe questionnaires by telephone) Check view about the number of questionnaires and clarity of questions<br><br>Check for questionnaire burden
**Allocation**	Which group do you think you were allocated?<br><br>Why?	**Do not say which.**<br>**The woman will receive notification about the allocation at the end of the study.**
	Thank you for taking part in the research study that will help inform a larger study.<br><br>Wish well for the future.	

Appendix 4: Participant Information Sheet

TIC TOC

Trial of intraoperative cell salvage versus transfusion in ovarian cancer – a feasibility study (the TIC TOC study)

Participant Information Sheet

We'd like to invite you to take part in our research study

- Before you decide whether to take part it is important for you to understand why the research is being done and what it would involve for you.
- Please take time to read the following information carefully. Discuss it with your family, friends or your family doctor (GP) if you wish.
- You are free to decide whether or not to take part in this study. If you choose not to take part, this will not affect the care you get from your doctors.
- Please ask us if anything is not clear, or if you would like [illegible]

Important information about this study

- This is a **feasibility study.** A feasibility study may be carried out before a main study in order to answer the question "Can this study be done?"
- The aim of this study is to find out whether we can successfully plan and carry out a larger study in the future.
- If you take part in this study you will be randomly allocated to receive **either** a reinfusion (return) of your own blood (called Intraoperative Cell Salvage) **or** a transfusion of donated blood (standard blood transfusion), if there is enough blood loss during your forthcoming operation.
- Your care and medications will continue as normal.
- The study involves completing some follow-up questionnaires which will be sent to you by post.

Key contents

	Page
What is the purpose of the study?	2
Donor blood transfusion	2
Intraoperative cell salvage (ICS)	2
Why have I been approached?	2
What does the study involve?	2
Do I have to take part?	4
Study flowchart	5
What are the possible risks?	6
General information	7

If you have any questions about this study please contact:

Your research nurse:

<Enter local contact name>

>Enter local contact details<

For peer review only - http://bmjopen.bmj.com/site/about/guidelines.xhtml

What is the purpose of the study?

During any surgical operation it is common for there to be some loss of blood. Sometimes this is significant enough for the patient to need a blood transfusion. Giving someone a blood transfusion involves transfusing donated blood from an anonymous healthy donor.

For a long time, donor blood has been the only choice available for blood transfusion. Although this is a very safe procedure in the UK it is not completely without risk. There is now a technique available in which blood lost by a patient during surgery is collected, washed and given back to the same patient. The technical name for this is **Intraoperative Cell Salvage** (ICS).

Donor blood transfusion

- Blood from an anonymous donor
- Washed and filtered in the blood bank

ICS blood reinfusion (ICS blood return)

- Patient's own blood
- Washed and filtered during the operation

Surgery is one of the main treatments for ovarian cancer. ICS blood return is already used successfully in other types of surgery and is being used in cancer surgery including some ovarian cancer operations. There is some evidence that using ICS blood return instead of donor blood transfusion promotes better recovery for patients after surgery. However, we do not know which method is better for patients undergoing ovarian cancer surgery. We also do not know which method is better value for money. This study will help us to answer those questions.

Why have I been approached to take part?

You are being invited to take part because you are due to have surgery for ovarian cancer, or suspected ovarian cancer, at one of the four participating hospitals and have been identified as being potentially suitable for the study. You cannot take part if: -

- You have any other diagnosed cancer
- You are pregnant
- You have any disease of the red blood cells such as sickle cell or thalassaemia
- You are unwilling to accept donor blood (e.g. on religious grounds)

What does the study involve?

This study will involve 60 women in total. Half of the women taking part in the study will be allocated to receive a donor blood transfusion. The other half will be allocated to receive an ICS blood return. All women will receive a brief telephone check-up by a research nurse approximately one month after surgery, and will also be asked to complete up to four study follow-up questionnaire booklets by post. There are more details about this on page 4. There are **no** extra hospital visits required.

For peer review only - http://bmjopen.bmj.com/site/about/guidelines.xhtml

What will happen to me if I agree to take part in the study?

If you agree to take part in the study after considering the information provided, you will be asked to sign a consent form before any study procedures are completed. Consent will usually be taken when you attend the routine pre-operative assessment clinic. A research nurse will then review your past and current health status and you will be asked to complete a questionnaire booklet about your general health and wellbeing, and how your illness is affecting your daily life. When you have your operation, you may be given blood replacement by either a donor blood transfusion (if there is enough blood loss) or you will be given an ICS blood return. The blood replacement you receive will depend on which method you are allocated. Some women may not require any blood replacement at all.

Who decides which type of blood replacement I receive?

If you consent to take part in the study, you will be allocated at random (by chance – like tossing a coin) by computer to receive either ICS blood return during your operation, or, if replacement blood is needed, a donor blood transfusion. This is called **randomisation**. All other aspects of your operation and care will be exactly the same as if you had decided not to be involved in the study. During the study, you will not be told which type of blood replacement you have received but you can find this out when the study has ended (more information is provided on page 7). In accordance with standard practice, women who receive donor blood will only be given a blood transfusion if there is enough blood loss during the operation. Women allocated to ICS blood will be given ICS blood return, even if there is only a little blood loss during the operation.

What if I am to receive chemotherapy before my surgery?

Your surgeon / doctor at the hospital will inform you if you require chemotherapy prior to your surgery and will provide you with the information you need. This is called neo-adjuvant chemotherapy. You can still take part in the study if you need chemotherapy first.

What happens if I do not require blood replacement?.

Women undergoing surgery for ovarian cancer (or suspected cancer) do not always require blood replacement, so some women taking part in this study will not receive blood of any sort. If you are one of these women, you are still very important to the study and we would still like to collect the same information from you.

For peer review only - http://bmjopen.bmj.com/site/about/guidelines.xhtml

Blood replacement after your operation

Sometimes it is usual for some patients to require blood replacement after their operation, either in the recovery unit or on the ward. If you need blood after surgery, this will be a donor blood transfusion regardless of whether you were in the group that received donor blood or your own ICS blood during your operation. This is because the ICS machine cannot be used on the hospital ward. If you require a donor transfusion after surgery your participation in the study will still be valid and it is important that vour information is still l included in the studv analvsis.

What happens after I've had my operation?

Participation in this study will not interfere with your usual care and recovery from surgery and should not delay your discharge home. If you take part in this study, the research nurse will record some details about your operation, recovery and whether or not you needed blood replacement. Any routine hospital follow-up visits will continue as usual.

Approximately **30 days** after your operation the research nurse will telephone you to ask about your general health and wellbeing since you were discharged from hospital. **Six weeks** after your operation you will be sent a questionnaire booklet to complete - the length of the booklet depends upon your final diagnosis. Your completed questionnaire booklet should be returned in the pre-paid envelope provided.

People who are recruited in the early stages of the study will be sent repeat questionnaire booklets, at three month intervals, as time allows (see flowchart on page 5). At these time points we will also ask you about any contacts you may have had with your hospital, GP, district nurse or other services since discharge from hospital. The questionnaire booklet should take no longer than 30 minutes to complete in total.

Do I have to take part?

No. Taking part in this study is entirely voluntary and it is up to you to decide whether or not to take part. If you do decide to take part you will be asked to sign a consent form, but you are still free to withdraw at any time in the future and without giving a reason. You will be given a copy of this information sheet and a copy of your signed consent form to keep. If you decide not to take part, or you withdraw from the study at any point, your medical care will not be affected in any way.

What will happen to me if I do NOT take part in the study?

If you decide this study is not for you at this time, you will be given the usual care and treatment offered at your hospital. If you need blood replacement during your operation, you may be offered ICS blood return as part of usual care (depending on the extent of your surgery

For peer review only - http://bmjopen.bmj.com/site/about/guidelines.xhtml

and the expected blood loss) even if you do not take part in the study. Usual care varies between hospitals because there is currently no evidence to show that receiving your own blood (ICS blood) is better or worse than donor blood. Currently ICS blood reinfusion is offered for ovarian cancer surgery in two of the hospitals participating in this study (Truro and Plymouth) but is not routinely offered in Gateshead or Leicester.

Study flowchart

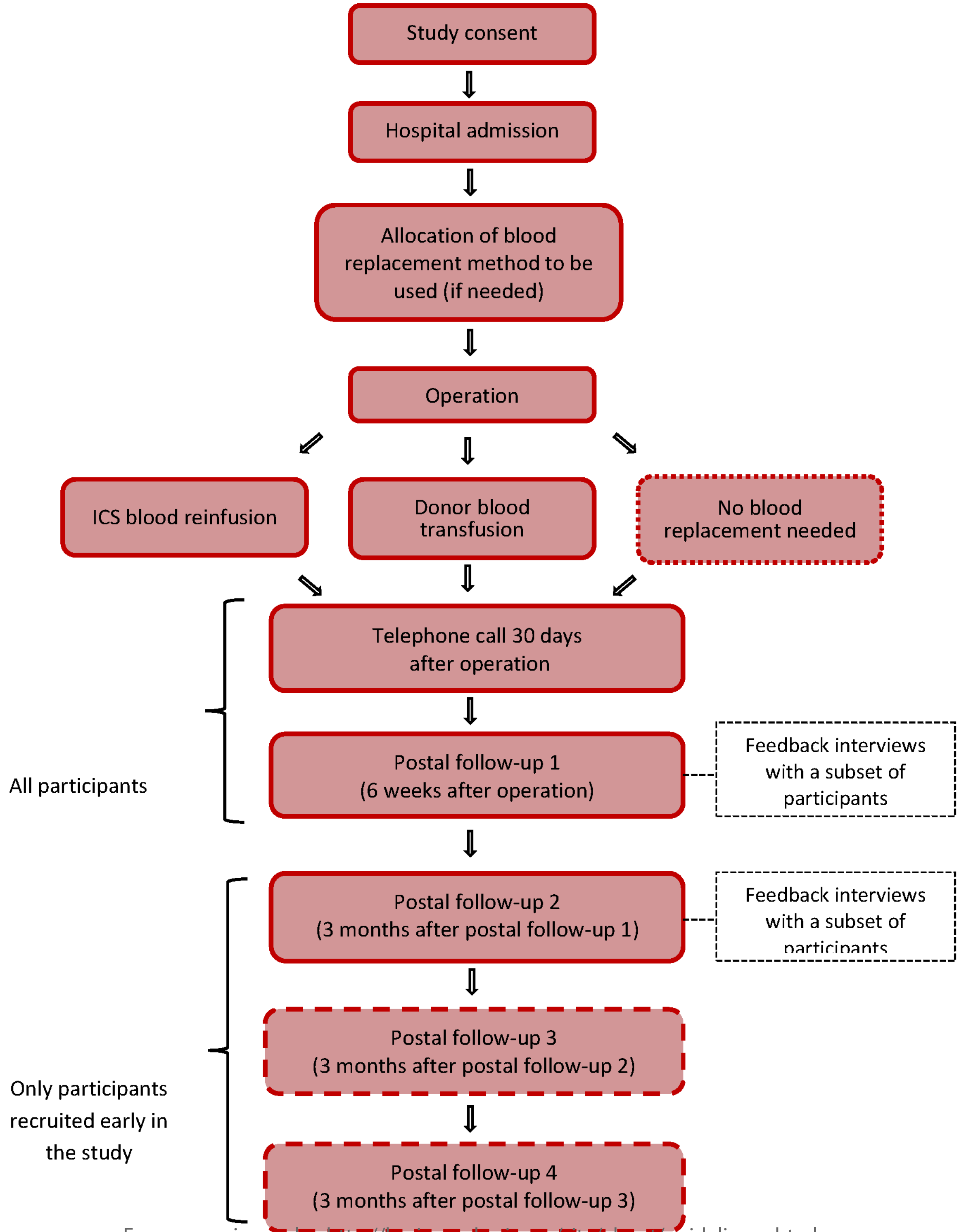

For peer review only - http://bmjopen.bmj.com/site/about/guidelines.xhtml

What are the possible benefits of taking part?

You may or may not benefit directly from this study but by taking part you will be contributing to a study which could potentially bring future benefit to women with ovarian cancer around the world. We don't know whether one method of blood replacement compared with the other improves recovery after surgery for ovarian cancer, but we hope that this study will help to answer that question.

What are the possible disadvantages and risks of taking part?

If you agree to take part in this study you will be asked to complete some questionnaire booklets, as described on page 4. These will take 15-30 minutes on each occasion and you will be asked to complete these two times (and up to a maximum of five times) over several months.

You may or may not receive blood replacement if you take part in this study. The anaesthetist and surgeon will decide whether you require additional blood during your operation as part of your usual care. If you need blood replacement during surgery, you may be given a donor blood transfusion or you may be given ICS blood return.

With any donor blood transfusion there is a possibility of side effects, including an increased risk of wound or other infections, lung and kidney problems, and a risk of receiving the wrong blood type in error. Such events and adverse transfusion reactions are rare. Donor blood transfusion has been used widely for many years and is considered a safe way of delivering blood to patients.

Intraoperative cell salvage (ICS) has been used in cancer operations, including ovarian cancer. Its use has been limited because of the theoretical risk (i.e. based on theory rather than experience) of reintroducing cancer cells into the bloodstream. However, the risk of cancer cells entering the bloodstream is low as far as current evidence shows, because a special filter is used which can remove any active cancer cells from the returned blood.

It is possible that the ICS technique may cause a temporary lowering of blood pressure but this is monitored continuously during the operation and any problem can be quickly corrected. There are no other documented problems with using ICS known to date.

For peer review only - http://bmjopen.bmj.com/site/about/guidelines.xhtml

What happens when the research study stops?

Once your participation in the study has ended, your usual care will continue as before. When every woman has completed their involvement in the study, we will prepare the study results (which normally takes several months) which will be available to participants.

If you would like to know whether you received a donor blood transfusion or ICS blood return, your research team will be able to tell you once everyone has completed the study. This is likely to be in the summer of 2018. The study results may be presented at national and international conferences and published in medical journals but you will not be identified in any information included in any presentation or publication.

General information about this study

What if relevant new information becomes available?

A special committee will be set up to look at all the information collected during the course of the TIC TOC study and will ensure that any study-related issues of concern are investigated. If the study is stopped for any reason, you will be told why. If any new information about ICS blood return becomes available which might affect your participation in the study, you will be informed.

What happens if I don't want to carry on with the study?

You are free to withdraw from the study at any time, without giving any reason, and without your medical care or legal rights being affected. If you want to withdraw from the study before you have your surgery, you must do this before you are given any anaesthetic. If you decide to withdraw from the study at any stage, we may still use information collected about you unless you ask us not to.

What if there is a problem?

Complaints: If you have a concern about any aspect of this study, please speak to someone in your research team who will do their best to answer your questions. If you remain unhappy and wish to complain formally, you can do this through your local NHS complaints procedure. The NHS has a Patient Advice and Liaison Service (PALS) for information and support, which can be found at your local hospital <Enter local PALS contact details>. You can also contact the department responsible for overseeing the study: Research, Development and

For peer review only - http://bmjopen.bmj.com/site/about/guidelines.xhtml

Innovations Manager, Knowledge Spa, Royal Cornwall Hospitals NHS Trust, Truro TR1 3HD. Tel: 01872 246424.

Harm: We don't expect any harm to come to you as a result of participating in this study. If you are harmed and this is due to someone's negligence, then you may have grounds for legal action for compensation against your hospital's Trust but you may have to pay your legal costs.

There are no special compensation arrangements in place. The normal NHS complaints mechanisms will still be available to you; your PALS service will be able to advise you.

Private insurance policies: Please note that it is your responsibility to check if taking part in this study affects the terms and conditions of any private insurance policies that you hold.

Will the information collected during the study be kept confidential?

All information collected about you whilst taking part in this study will be kept strictly confidential and will be collected and stored for five years after the study is complete, in accordance with the Data Protection Act (1998). You will be given a unique study number which your study information will be labelled with, along with your initials, so that you cannot be identified (known as pseudonymised or de-identified data). This study information will be stored and analysed at Plymouth University. Only members of the research team and the Peninsula Clinical Trials Unit (PenCTU) at Plymouth University will have direct access to the study information. Paper-based information will be stored in locked filing cabinets within a locked office in the PenCTU. Information kept on computers will be stored securely on a system maintained by Plymouth University. Copies of the study information will be held securely at your local hospital but will not contain any details that could identify you.

Authorised people from your NHS Trust, the PenCTU and study organisers may need to review your medical records to check that the study is being carried out correctly. All will have a duty of confidentiality to you as a research participant. As part of the consent process, you will be asked to consent to your contact details (name, address, telephone number) being provided to the PenCTU and the TIC TOC researcher based at Royal Cornwall Hospitals NHS Trust, to enable collection of some information by post. At the PenCTU, these details will be stored separately from the de-identified study information also held.

For peer review only - http://bmjopen.bmj.com/site/about/guidelines.xhtml

Will the study information help with other research projects?

It is important that good quality research data can be shared with others in order to advance clinical research and to benefit patients in the future. After the end of the study, de-identified information collected during the study will be made available to other researchers under an appropriate data sharing agreement, but it will not be possible to identify you personally from any information shared.

This is a feasibility study, so the aim is to test the processes required for a large study. This study will provide us with the necessary information to help us to learn what to consider when designing a main study in the future. Ultimately, the main study will assess whether ICS or donor blood transfusion is associated with better outcomes for patients having ovarian cancer surgery.

Involvement of your General Practitioner/ Family Doctor (GP)

Your general practitioner will be informed of your participation in this study.

Who is organising and funding the study?

The study is being led by Miss Khadra Galaal, Consultant Gynaecological Oncologist at the Royal Cornwall Hospitals NHS Trust (RCHT). The study is funded by the National Institute for Health Research (NIHR) Research for Patient Benefit grant scheme Ref: PB-PG-1014-35005. The study will be managed by the Peninsula Clinical Trials Unit at Plymouth University and sponsored (overseen) by RCHT.

Who has reviewed the study?

All NHS research is looked at by an independent panel (Research Ethics Committee). This study has been reviewed and been given a favourable opinion by the <Enter name> Research Ethics Committee.

Thank you for taking the time to read this information sheet and for considering taking part in the TIC TOC study.

For peer review only

TIC TOC

TO BE PRINTED ON RELEVANT NHS TRUST HEADED PAPER

Appendix 5: Informed consent form

PARTICIPANT CONSENT FORM

A randomised, controlled feasibility trial of intraoperative cell salvage versus donor blood transfusion in ovarian cancer surgery

Principal Investigator: <Insert PI's name> **Participant Study Number:** ☐☐☐☐

Please initial each box

1. I confirm that I have read and understood the information sheet (version 2.0, dated 09 October 2017) for the above study. I have had the opportunity to consider the information and ask questions and I have had my questions answered satisfactorily. ☐
2. I understand that my participation in this study is voluntary and that I am free to withdraw at any time without giving any reason and without my medical care or legal rights being affected. ☐
3. I agree that my name, address and telephone number can be given to and stored by the Peninsula Clinical Trials Unit at Plymouth University to enable collection of study information by post. ☐
4. I understand that relevant sections of any of my medical notes and information collected during the study may be looked at by responsible individuals from my local NHS Trust, the Peninsula Clinical Trials Unit and the regulatory authorities, where it is relevant to my taking part in this research. I give permission for these individuals to have access to my records. ☐
5. I understand that an anonymised copy of this consent form will be sent to the Peninsula Clinical Trials Unit to confirm my agreement to participate. ☐
6. I understand that the information collected about me may be shared anonymously with other researchers to support future research studies. I cannot be personally identified from this. ☐
7. I agree to take part in the TIC TOC study. ☐

________________________	________________________	________________________
Print Name (Participant)	*Date*	*Signature*

________________________	________________________	________________________
Print Name (Researcher taking consent)	*Date*	*Signature*

For peer review only - http://bmjopen.bmj.com/site/about/guidelines.xhtml

SPIRIT

STANDARD PROTOCOL ITEMS: RECOMMENDATIONS FOR INTERVENTIONAL TRIALS

SPIRIT 2013 Checklist: Recommended items to address in a clinical trial protocol and related documents*

Section/item	Item No	Description	Addressed on page number
**Administrative information**			
Title	1	Descriptive title identifying the study design, population, interventions, and, if applicable, trial acronym	_____1________
Trial registration	2a	Trial identifier and registry name. If not yet registered, name of intended registry	_____2_____
	2b	All items from the World Health Organization Trial Registration Data Set	1-23, as applicable
Protocol version	3	Date and version identifier	9 Oct 2017, not included
Funding	4	Sources and types of financial, material, and other support	______22_______
Roles and responsibilities	5a	Names, affiliations, and roles of protocol contributors	______1, 21-22__
	5b	Name and contact information for the trial sponsor	______22_______
	5c	Role of study sponsor and funders, if any, in study design; collection, management, analysis, and interpretation of data; writing of the report; and the decision to submit the report for publication, including whether they will have ultimate authority over any of these activities	_____22_____
	5d	Composition, roles, and responsibilities of the coordinating centre, steering committee, endpoint adjudication committee, data management team, and other individuals or groups overseeing the trial, if applicable (see Item 21a for data monitoring committee)	_____14________

For peer review only

Introduction

Background and rationale	6a	Description of research question and justification for undertaking the trial, including summary of relevant studies (published and unpublished) examining benefits and harms for each intervention	______4-6_____
	6b	Explanation for choice of comparators	____4, 5_______
Objectives	7	Specific objectives or hypotheses	_____6________
Trial design	8	Description of trial design including type of trial (eg, parallel group, crossover, factorial, single group), allocation ratio, and framework (eg, superiority, equivalence, noninferiority, exploratory)	______6, 7______

Methods: Participants, interventions, and outcomes

Study setting	9	Description of study settings (eg, community clinic, academic hospital) and list of countries where data will be collected. Reference to where list of study sites can be obtained	_____7________
Eligibility criteria	10	Inclusion and exclusion criteria for participants. If applicable, eligibility criteria for study centres and individuals who will perform the interventions (eg, surgeons, psychotherapists)	_____ 8_______
Interventions	11a	Interventions for each group with sufficient detail to allow replication, including how and when they will be administered	_____9_______
	11b	Criteria for discontinuing or modifying allocated interventions for a given trial participant (eg, drug dose change in response to harms, participant request, or improving/worsening disease)	____9_________
	11c	Strategies to improve adherence to intervention protocols, and any procedures for monitoring adherence (eg, drug tablet return, laboratory tests)	_____N/A_______
	11d	Relevant concomitant care and interventions that are permitted or prohibited during the trial	_____9-10______
Outcomes	12	Primary, secondary, and other outcomes, including the specific measurement variable (eg, systolic blood pressure), analysis metric (eg, change from baseline, final value, time to event), method of aggregation (eg, median, proportion), and time point for each outcome. Explanation of the clinical relevance of chosen efficacy and harm outcomes is strongly recommended	_____10, 11_____
Participant timeline	13	Time schedule of enrolment, interventions (including any run-ins and washouts), assessments, and visits for participants. A schematic diagram is highly recommended (see Figure)	_____12________

For peer review only - http://bmjopen.bmj.com/site/about/guidelines.xhtml

Sample size	14	Estimated number of participants needed to achieve study objectives and how it was determined, including clinical and statistical assumptions supporting any sample size calculations	____14_______
Recruitment	15	Strategies for achieving adequate participant enrolment to reach target sample size	_____N/A______

Methods: Assignment of interventions (for controlled trials)

Allocation:

Sequence generation	16a	Method of generating the allocation sequence (eg, computer-generated random numbers), and list of any factors for stratification. To reduce predictability of a random sequence, details of any planned restriction (eg, blocking) should be provided in a separate document that is unavailable to those who enrol participants or assign interventions	_____8________
Allocation concealment mechanism	16b	Mechanism of implementing the allocation sequence (eg, central telephone; sequentially numbered, opaque, sealed envelopes), describing any steps to conceal the sequence until interventions are assigned	_____8________
Implementation	16c	Who will generate the allocation sequence, who will enrol participants, and who will assign participants to interventions	______7, 8______
Blinding (masking)	17a	Who will be blinded after assignment to interventions (eg, trial participants, care providers, outcome assessors, data analysts), and how	____10_______
	17b	If blinded, circumstances under which unblinding is permissible, and procedure for revealing a participant's allocated intervention during the trial	____N/A_______

Methods: Data collection, management, and analysis

Data collection methods	18a	Plans for assessment and collection of outcome, baseline, and other trial data, including any related processes to promote data quality (eg, duplicate measurements, training of assessors) and a description of study instruments (eg, questionnaires, laboratory tests) along with their reliability and validity, if known. Reference to where data collection forms can be found, if not in the protocol	____12, 13______
	18b	Plans to promote participant retention and complete follow-up, including list of any outcome data to be collected for participants who discontinue or deviate from intervention protocols	_____13_______

Data management	19	Plans for data entry, coding, security, and storage, including any related processes to promote data quality (eg, double data entry; range checks for data values). Reference to where details of data management procedures can be found, if not in the protocol	____12,13_____
Statistical methods	20a	Statistical methods for analysing primary and secondary outcomes. Reference to where other details of the statistical analysis plan can be found, if not in the protocol	____15______
	20b	Methods for any additional analyses (eg, subgroup and adjusted analyses)	_____N/A_______
	20c	Definition of analysis population relating to protocol non-adherence (eg, as randomised analysis), and any statistical methods to handle missing data (eg, multiple imputation)	_______N/A______
**Methods: Monitoring**			
Data monitoring	21a	Composition of data monitoring committee (DMC); summary of its role and reporting structure; statement of whether it is independent from the sponsor and competing interests; and reference to where further details about its charter can be found, if not in the protocol. Alternatively, an explanation of why a DMC is not needed	_____14_______
	21b	Description of any interim analyses and stopping guidelines, including who will have access to these interim results and make the final decision to terminate the trial	_____N/A_______
Harms	22	Plans for collecting, assessing, reporting, and managing solicited and spontaneously reported adverse events and other unintended effects of trial interventions or trial conduct	__13-14________
Auditing	23	Frequency and procedures for auditing trial conduct, if any, and whether the process will be independent from investigators and the sponsor	_____N/A_______
**Ethics and dissemination**			
Research ethics approval	24	Plans for seeking research ethics committee/institutional review board (REC/IRB) approval	_______2______
Protocol amendments	25	Plans for communicating important protocol modifications (eg, changes to eligibility criteria, outcomes, analyses) to relevant parties (eg, investigators, REC/IRBs, trial participants, trial registries, journals, regulators)	_____N/A_______

Consent or assent	26a	Who will obtain informed consent or assent from potential trial participants or authorised surrogates, and how (see Item 32)	____7_________
	26b	Additional consent provisions for collection and use of participant data and biological specimens in ancillary studies, if applicable	____N/A________
Confidentiality	27	How personal information about potential and enrolled participants will be collected, shared, and maintained in order to protect confidentiality before, during, and after the trial	____12 -13_____
Declaration of interests	28	Financial and other competing interests for principal investigators for the overall trial and each study site	____22__________
Access to data	29	Statement of who will have access to the final trial dataset, and disclosure of contractual agreements that limit such access for investigators	_____19________
Ancillary and post-trial care	30	Provisions, if any, for ancillary and post-trial care, and for compensation to those who suffer harm from trial participation	______19_______
Dissemination policy	31a	Plans for investigators and sponsor to communicate trial results to participants, healthcare professionals, the public, and other relevant groups (eg, via publication, reporting in results databases, or other data sharing arrangements), including any publication restrictions	______19_______
	31b	Authorship eligibility guidelines and any intended use of professional writers	______N/A______
	31c	Plans, if any, for granting public access to the full protocol, participant-level dataset, and statistical code	______N/A______
**Appendices**			
Informed consent materials	32	Model consent form and other related documentation given to participants and authorised surrogates	Appendix pp5 -14
Biological specimens	33	Plans for collection, laboratory evaluation, and storage of biological specimens for genetic or molecular analysis in the current trial and for future use in ancillary studies, if applicable	_______N/A_____

*It is strongly recommended that this checklist be read in conjunction with the SPIRIT 2013 Explanation & Elaboration for important clarification on the items. Amendments to the protocol should be tracked and dated. The SPIRIT checklist is copyrighted by the SPIRIT Group under the Creative Commons "Attribution-NonCommercial-NoDerivs 3.0 Unported" license.

For peer review only - http://bmjopen.bmj.com/site/about/guidelines.xhtml

Trial of intraoperative cell salvage versus transfusion in ovarian cancer (TIC TOC): protocol for a randomised controlled feasibility study

Journal:	*BMJ Open*
Manuscript ID	bmjopen-2018-024108.R2
Article Type:	Protocol
Date Submitted by the Author:	21-Sep-2018

Complete List of Authors:	Galaal, Khadra; Royal Cornwall Hospitals NHS Trust, Gynaeoncology<br>Lopes, Alberto; Royal Cornwall Hospitals NHS Trust<br>Pritchard, Colin; Royal Cornwall Hospitals NHS Trust, Research, Development and Innovation<br>Barton, Andy; NIHR South West Research Design Service<br>Wingham, Jennifer; University of Exeter Medical School,<br>Marques, Elsa; University of Bristol, School of Social and Community Medicine<br>Faulds, John; Royal Cornwall Hospitals NHS Trust<br>Palmer, Joanne; Hull York Medical School, Vascular Research Unit<br>Vickery, Patricia; Plymouth University, Peninsula Clinical Trials Unit<br>Ralph, Catherine; Royal Cornwall Hospitals NHS Trust, Anaesthetics<br>Ferreira, Nicole ; Royal Cornwall Hospitals NHS Trust<br>Ewings, Paul; Research Design Service, Research Office

**Primary Subject Heading**:	Obstetrics and gynaecology
Secondary Subject Heading:	Haematology (incl blood transfusion)
Keywords:	intraoperative cell salvage, donor blood transfusion, cytoreductive surgery, ovarian cancer, feasibility trial, quality of life

SCHOLARONE™
Manuscripts

Trial of intraoperative cell salvage versus transfusion in ovarian cancer (TIC TOC): protocol for a randomised controlled feasibility study

Khadra Galaal[1], Alberto Lopes[2], Colin Pritchard[3], Andrew Barton[4], Jennifer Wingham[2], Elsa Marques[5], John Faulds[6], Joanne Palmer[3], Jane Vickery[7], Catherine Ralph[9], Nicole Ferreira[6], Paul Ewings[4].

1. Gynaecology, Royal Cornwall Hospitals NHS Trust, Truro, Cornwall, UK
2. University of Exeter, Devon, UK
3. Research, Development and Innovation, Royal Cornwall Hospitals NHS Trust, Truro, Cornwall, UK
4. NIHR Research Design Service (South West)
5. Bristol Medical School, University of Bristol, Bristol, UK
6. Royal Cornwall Hospitals NHS Trust, Truro, Cornwall, UK
7. Peninsula Clinical Trials Unit, Plymouth University, Plymouth, UK
8. Department of Anaesthesia, Royal Cornwall Hospitals NHS Trust, Truro, Cornwall, UK.

Corresponding Author

Miss Khadra Galaal FRCOG

Consultant Gynaecological Oncologist

Royal Cornwall Hospital, Truro, UK

Tel: 01872 252731

Email: k.galaal@nhs.net

Key words: intraoperative cell salvage, donor blood transfusion, cytoreductive surgery, ovarian cancer, feasibility trial, economic evaluation, quality of life.

For peer review only

ABSTRACT

Introduction: Ovarian cancer is the leading cause of death from gynaecological cancer, with more than 7,000 new cases registered in the UK in 2014. In patients suitable for surgery, NICE guidance for treatment recommends surgical resection of all macroscopic tumour, followed by chemotherapy. The surgical procedure can be extensive and associated with substantial blood loss which is conventionally replaced with a donor blood transfusion. Whilst often necessary and life-saving, the use of donor blood is associated with increased risks of complications and adverse surgical outcomes. Intraoperative cell salvage (ICS) is a blood conservation strategy in which red cells collected from blood lost during surgery are returned to the patient thus minimising the use of donor blood. This is the protocol for a feasibility randomised controlled trial with an embedded qualitative study and feasibility economic evaluation. If feasible, a later definitive trial will test the effectiveness and cost-effectiveness of ICS reinfusion versus donor blood transfusion in ovarian cancer surgery.

Methods and analysis: Sixty adult females scheduled for primary or interval ovarian cancer surgery at participating UK NHS Trusts will be recruited and individually randomised in a 1:1 ratio to receive intraoperative cell salvage reinfusion or donor blood (as required) during surgery. Participants will be followed up by telephone at 30 days post-operatively for adverse events monitoring and by postal questionnaire at six weeks and three monthly thereafter, to capture quality of life and resource use data. Qualitative interviews will capture participants' and clinicians' experiences of the study.

Ethics and dissemination: This study has been granted ethical approval by the South West - Exeter Research Ethics Committee (ref: 16/SW/0256). Results will be disseminated via peer-reviewed publications and will inform the design of a larger trial.

Trial registration number: ISRCTN19517317

For peer review only

For peer review only - http://bmjopen.bmj.com/site/about/guidelines.xhtml

Strengths and limitations of this study

- This is the first study to use intraoperative cell salvage in cytoreductive surgery for ovarian cancer
- The study explores the feasibility and informs the design of a larger randomised controlled trial. Quantitative, qualitative and feasibility economic components are included
- Limitations are;
    - The effect of transfusion and cell salvage on immune response to surgery is not assessed
    - This feasibility study will not provide information on the long-term outcomes of using either cell salvage or transfusion.

For peer review only

For peer review only - http://bmjopen.bmj.com/site/about/guidelines.xhtml

INTRODUCTION

Background

Ovarian cancer is the leading cause of death from gynaecological cancer in the UK (age-standardised mortality rate 9.1 per 100,000 2008-2010) (1). Although survival rates have improved in recent decades, there are still more deaths from ovarian cancer than all other gynaecological cancers combined (2). The mainstays of treatment for advanced ovarian cancer are surgical cytoreduction and platinum-based chemotherapy. As operative success and survival is largely determined by residual disease (3). Surgery is often extensive with substantial intraoperative blood loss, about 53% of patients lose more than 1.5 litres during their first surgery (4). Blood lost during surgery is conventionally replaced using donor blood transfusion with the incidence of transfusion ranging from 35% to 77% (5, 6). Perioperative donor blood transfusion is associated with increased risks of complications and adverse surgical outcomes including mortality, wound infection, pulmonary and renal complications, systemic sepsis and prolonged hospital stay (7). In 2012 there were 12.3 serious adverse incidents per 10,000 transfused components reported by the Serious Hazards of Transfusion (SHOT) group (8). SHOT is an independent, professionally-led scheme, involved in collecting and analysing anonymised information on adverse events and reactions in blood transfusion from all healthcare organisations in the U.K. Where risks and problems are identified, they produce recommendations to improve patient safety. One suggested explanation for adverse reactions is a general transient depression of the immune system following transfusion with blood products, transfusion-induced immunomodulation (TRIM) (9, 10).

Intraoperative cell salvage (ICS) or autologous blood transfusion is the practice of recovering red cells from blood lost in the operative field and returning them to the patient (11). This process involves the separation, centrifugation, washing and filtration of heparinised red blood cells, before reinfusion into the patient. ICS eliminates or reduces the need for donor blood transfusion and its associated risks, making it an alternative where major blood loss is anticipated (12). ICS can be available in theatre at modest expense and reduces dependence on the limited pool of banked blood.

For peer review only - http://bmjopen.bmj.com/site/about/guidelines.xhtml

For peer review only

Studies comparing cell salvage with allogeneic blood transfusion have demonstrated increased mean erythrocyte (red blood cells) viability as high as 88% with cell salvage (13-15). ICS has been used successfully in surgical specialties (16) including cardiothoracic, vascular, orthopaedic and hepatobiliary (17-20). In addition, intraoperative cell salvage is associated with low rate of patient-related adverse events (21). ICS was initially contraindicated in cancer because of the theoretical risk of reintroducing malignant tumour cells into patients' bloodstreams (22, 23). However, such concerns appear to be unfounded(24). The *in vitro,* leucocyte depletion filters are highly efficient at removing malignant cells with removal rates of between 80 and 100% (25, 26). In patients undergoing surgery for gynaecological malignancy, leucocyte depletion filters effectively eliminate viable nucleated malignant cells from the returned blood (27, 28). Far from compromising outcomes, ICS is associated with improved outcomes in cervical (29, 30) and oesophageal cancers (24).

Interestingly, patients with primary metastatic cancer are known to have circulating tumour cells in the blood (31). Furthermore, operative manipulation of tumours during surgery leads to peripheral blood concentrations of malignant cells many times higher than could be attained with cell salvage (32). The presence of circulating tumour cells is prevalent in cancer patients with approximately one circulating tumour cell (CTC) per 105 to 107 mononuclear cells found in the peripheral blood of metastatic cancer patients (33).

Rationale

There is a paucity of studies in ICS, making it difficult for patients, clinicians and NHS managers to make decisions about this technology (34). ICS has been used in ovarian cancer patients in one of the participating sites with encouraging results, but a randomised controlled trial (RCT) is required for robust determination of effectiveness. The aim of a definitive trial would be to assess the clinical and cost-effectiveness of intraoperative cell salvage for women undergoing cytoreductive surgery for ovarian cancer, compared with usual practice of transfusing only allogeneic blood as required.

For peer review only - http://bmjopen.bmj.com/site/about/guidelines.xhtml

Aim and objectives

The aim of the study is to determine whether a definitive randomised controlled trial is feasible and, if so, how best to deliver it. The objectives of the study are to:

- Estimate the likely recruitment rate for the larger trial
- Estimate the likely completeness of resource use and outcome data
- Explore the practical logistics of undertaking randomisation in theatres
- Assess success of blinding of allocation for participants and outcome assessors
- Design data collection tools to collect resource use data from participants, hospital medical records and hospital staff
- Inform the trial design and confirm the resources required to run a larger definitive trial
- Explore the barriers and facilitators for women when deciding whether or not to participate
- Explore women's perceptions of:
    - The intervention, the information given and advantages/disadvantages of participation so that information can be optimised for the larger trial
    - Other trial aspects, e.g. regarding collection of outcome measures and completing resource use questionnaires.
- Identify factors influencing surgeons' decisions about whether or not to participate in the study.

METHODS

Trial design

This is a protocol for a randomised, controlled, multi-centre feasibility study in women undergoing cytoreductive surgery for ovarian cancer. Sixty participants will be individually randomised in a 1:1 ratio to intraoperative cell salvage (re-infusion of their own blood) or donor blood transfusion during surgery. Participants and outcome assessors will be blinded to the intervention. All participants will be followed up by telephone for adverse events reporting at 30 days post-

For peer review only - http://bmjopen.bmj.com/site/about/guidelines.xhtml

operatively, by post six weeks post-operatively and three monthly thereafter as time allows. A schematic diagram of the trial is given in Figure 1. The feasibility study includes an embedded qualitative component to assess participants' (patients and clinicians) perceptions of their experience in preparation for the later trial. It will also involve an assessment of the feasibility of collecting resource use and other economic data for a future economic evaluation.

Study setting

The study will take place at the Royal Cornwall Hospitals NHS Trust, Plymouth Hospitals NHS Trust, Gateshead Health NHS Foundation Trust and University Hospitals of Leicester NHS Trust. All sites have existing personnel experienced in the management of intraoperative cell salvage and reinfusion.

Participants and recruitment

Participants will be recruited from patients scheduled to undergo surgery for ovarian cancer at the participating hospitals. Potential participants will usually be identified from those patients attending the gynaecological oncology out-patient clinic having been referred by their GP under the two week wait cancer pathway. Some patients will be scheduled for primary surgery and are suitable for immediate recruitment to the study. Others will undergo neo-adjuvant chemotherapy prior to interval debulking surgery and may be recruited to the study at a later date, following chemotherapy. Written informed consent (Appendix 1 and 2) will be obtained by an appropriately trained member of the research team in line with ICH Good Clinical Practice (GCP) guidelines. As part of the consent process, patients will be reminded that they are free to withdraw from the study at any time without giving a reason and without affecting their future treatment.

Inclusion criteria

Potential participants must satisfy the following criteria to be enrolled in the study:

• 18 years old or over

For peer review only

For peer review only - http://bmjopen.bmj.com/site/about/guidelines.xhtml

• Suspected or confirmed ovarian cancer (newly diagnosed) requiring cytoreductive surgery, whether primary or interval (following chemotherapy)

• CT scan evidence (with or without clinical evidence) compatible with FIGO stage III/IV ovarian cancer/primary peritoneal cancer at presentation (35) (Appendix 3)

• Eastern Cooperative Oncology Group (ECOG) Performance Status 0-1 (36)

• Willing to participate and able to give written informed consent

Exclusion criteria

Potential participants meeting any of the following criteria will be excluded from study participation:

• Diagnosis of concurrent malignancy

• Pregnant

• Haemoglobinopathies (e.g. sickle cell, thalassaemia)

• Unwilling to accept donor blood (e.g. on religious grounds)

Randomisation

Randomisation will be undertaken after written consent has been obtained, but as close to the start of surgery as possible; usually this will be on the morning of the operation day but if this is not possible for practical reasons, it may be performed earlier. Randomisation will be achieved by means of a web-based system created by the UK Clinical Research Collaboration (UKCRC) registered Peninsula Clinical Trials Unit (CTU) in conjunction with the trial statistician, using random permuted blocks of varying size. Participants will be allocated to receive ICS reinfusion or donor blood transfusion in a 1:1 ratio, stratified by study site. To prevent any unnecessary delays in the operating theatre, cell salvage equipment will be set up in advance for all study participants, before confirmation of treatment allocation.

Trial interventions

For peer review only - http://bmjopen.bmj.com/site/about/guidelines.xhtml

Participants will be allocated to receive either donor blood transfusion or ICS reinfusion intraoperatively, in accordance with specified transfusion protocols. Donor blood will only be given (in standard volumes) when deemed necessary (e.g. after substantial blood loss and/or drop in haemoglobin) whereas ICS blood will be returned even if only small quantities are lost. Some participants may not require any intraoperative transfusion and some (in either arm of the trial) may require donor blood transfusion post-operatively.

Intraoperative cell salvage

All sites will follow a common ICS protocol and relevant site staff will undergo study-specific training prior to the study start. Collected blood will be processed via the ICS machine before being re-infused via a leucodepletion filter. The make and model of ICS machine and leucodepletion filter used in clinical practice varies across NHS Trusts and will not be standardised for this feasibility study. Relevant data from a local intraoperative cell salvage audit form will be transcribed into the study-specific Case Report Form (CRF), including the amounts of salvaged blood processed and reinfused.

Donor transfusion

Participants allocated to donor transfusion will be considered for transfusion during surgery in accordance with clinical judgement, guided by local hospital policy. The factors triggering transfusion (e.g. excessive blood loss, hypotension, reduced Hb) will be documented in the CRF along with the amount and type of blood and blood products transfused.

Donor transfusion in ICS arm

Participants allocated to the ICS arm who need donor transfusion can be given donor blood at any time, during or after surgery, for the duration of their hospital stay. The factors triggering intraoperative donor transfusion in the ICS group will be documented in the CRF as well as the amount and type of any blood and blood products transfused.

Blinding

For peer review only

Surgeons, other theatre staff and the person recording details of intra-operative blood transfusion or reinfusion cannot be blinded in this study. The research nurse responsible for recording post-operative outcomes will aim to remain blinded to treatment allocation. Participants in either arm of the study may have some form of blood replacement in progress immediately post-surgery; it is unlikely that participants will be able to distinguish between the two types and either group may require donor blood for clinical reasons.

Feasibility outcomes

The outcomes for this study are the feasibility and acceptability of the study and study procedures in relation to recruitment, randomisation, intervention, blinding, participant retention and data completion. Both quantitative and qualitative methods will be used. Recruitment rate will be measured as the proportion of eligible patients who are subsequently enrolled and the number of patients recruited per site per month. The number of patients screened, number/percent of patients approached, number/percent of patients excluded after screening/approach and the number/percent of patients providing consent will be assessed. Reasons for declining participation will be sought where possible, and the appropriateness and practicalities of the chosen eligibility criteria will be explored. The number/percent of women enrolled prior to initial surgery compared to following neo-adjuvant chemotherapy will be assessed. The timing of randomisation in relation to operation start will be recorded to assess the practicalities of randomising as late as possible, in particular what proportion are randomised on the day of surgery itself.

Use of ICS blood and donor blood will be recorded for both arms, partly to assess intervention fidelity but also to obtain an estimate of the proportion of people in the control arm that actually require donor blood. Reasons for non-use of ICS blood and/or use of donor blood in the ICS arm will be recorded.

Since the intervention takes place in the operating theatre it is unlikely that any participant will withdraw from intervention following randomisation. Attrition will be assessed by examining the

For peer review only - http://bmjopen.bmj.com/site/about/guidelines.xhtml

number of participants lost to follow-up at any subsequent point in the study period. Reasons for discontinuation of follow-up will be sought from participants.

The success of blinding of allocation for participants and outcome assessors will be assessed by asking both the participant and research nurse to guess the allocation (including "unsure") at the 30 day post-operative follow-up and comparing the responses with the actual allocation.

Clinical outcomes

In the later, definitive trial, our primary outcome is likely to be either mortality or cancer recurrence, both of which are unlikely to occur in the time available in this feasibility study. Therefore, whilst readily accessible, these data will not be collected here. Other measures proposed for the later trial will be collected in this feasibility study at baseline and peri-operatively, with follow-up at 30 days and 6 weeks post-operatively. Participants recruited at an early stage of the study will also be followed up at 4.5, 7.5 and 10.5 months post-operatively as time allows (Figure 1). Clinical outcomes include:

- Inadvertent visceral injury (bladder, bowel, ureters, blood vessels, nerve)
- Return to theatre within 48 hours
- Surgical site infection (Appendix 4) within 30 days
- Thromboembolic complications (DVT, PE) within 30 days
- Number and nature of adverse events
- Amount of donor blood given (total and ≤24 hours post-surgery)
- Length of hospital stay
- Resource use
- Generic quality of life (QOL) measure: EQ-5D-5L
- Cancer-specific QOL measure: EORTC QLQ-C30 (Version 3.0) (confirmed cancer only)
- Ovarian cancer QOL measure: EORTC QLQ-OV28 (confirmed cancer only)

Data management

For peer review only

For peer review only - http://bmjopen.bmj.com/site/about/guidelines.xhtml

Each participant will be allocated a unique trial number on consenting to the study and will be identified in all study-related documentation by her trial number and initials. A record of names and addresses linked to participants' trial numbers will be maintained by the research nurse at each site for administrative purposes, and stored securely.

Data collection

Data collected by the research team (Table 1) up to 30 days post-operatively will be recorded on study specific data collection forms (CRFs), usually by a research nurse. All data not routinely captured during the hospital admission but recorded straight into the CRF will be classified as source data. Participant self-completion questionnaires at baseline will be completed during a face-to-face meeting with a research nurse, following written informed consent. The research nurse will return completed CRFs and baseline questionnaires to the CTU. Subsequent self-completion questionnaires (6 weeks post-operatively and three monthly thereafter as time allows) will be mailed to participants directly from the CTU and returned by participants to the CTU in a pre-paid envelope provided. In the event of non-return of a questionnaire, a reminder will be sent from the CTU in the first instance. If there is no response from the two mailings, the CTU will inform the local research nurse who will telephone the participant in order to encourage compliance with follow-up.

For peer review only

Table 1: Trial schedule

	Pre-operative		OPERATION and peri-operative data collection	Post-operative follow-up				
	Screen	Baseline		1	2	3†	4†	5†
				30 days post-op	6 weeks post-op	3 months after follow-up 2	6 months after follow-up 2	9 months after follow-up 2
Screen/eligibility	x							
Consent		x						
Demographics & history		x						
Randomisation		x						

EORTC QLQ-C30*		x			x	x	x	x
EORTC QLQ-OV28*		x			x	x	x	x
EQ-5D-5L		x			x	x	x	x
Adverse events				x				
Resource use questionnaire		x			x	x		
Qualitative interviews					x	x		

Statistical considerations

Sample size for a feasibility study is necessarily a compromise between the twin assets of precision and efficiency. For any binary "outcome" our target sample size of 60 will result in a 95% confidence interval of no greater than about +/-12 percentage points, while in a single arm the target of 30 will have a CI of no more than +/- 17 percentage points.

Data analysis will enable the feasibility outcomes to be addressed in order to inform a decision about proceeding to a definitive trial. Data will be presented in accordance with the extension to the ConSORT statement for pilot and feasibility studies. They will detail the numbers of patients that were approached, the number that were eligible and the number providing consent. Likewise, compliance rates at all stages will be presented, including the numbers of questionnaires completed at each stage and more generally the completeness of data on all outcomes at each time point. Participating patients' characteristics (demographics, comorbidities, clinical details) will be summarised and, where possible, compared with the overall population of relevant patients to explore possible factors associated with participation. Where possible, the reasons will be ascertained for potentially eligible patients not being approached to consider participation.

Descriptive data on the clinical outcomes will be presented by trial arm, using appropriate measures of central tendency and variation for continuous measures and numbers/percentages for categorical measures. No formal statistical tests will be conducted.

Qualitative study

For peer review only

For peer review only - http://bmjopen.bmj.com/site/about/guidelines.xhtml

A qualitative evaluation will assess the acceptability of the intervention to women taking part in the study, in particular attitudes towards reinfusion of salvaged blood and transfusion of donor blood. The study will also gain an understanding of the women's experience of taking part in the research processes of the TIC TOC study and what influenced their decision to take part. Following surgery, up to 20 women from across all centres will be asked to take part in individual face to face or telephone semi-structured interviews using a topic guide that has been developed with patient and public involvement (PPI) involvement (Appendix 5). Purposive sampling techniques will ensure a range of women are selected according to centre, education, age, ethnicity, socioeconomic status, and social support.

As the trial schedule allows, the same women will be approached to take part in a brief telephone interview three months after the first interview. The purpose of the second interview is to determine participants' perceptions about the follow-up research processes and ask their opinion about whether anything should change in a full trial. Surgeons from each centre will also be invited to participate in one brief telephone interview each to understand the issues considered in deciding whether to offer women the opportunity to take part in the study.

The qualitative data will be managed using computer software such as Nvivo 11 and thematically analysed (37, 38). The researcher will ensure accuracy of the transcription and read the transcript several times to become immersed in the data, noting initial thoughts and ideas. Codes will be assigned to extracts of the data relevant to the project. Codes with similar meaning will be grouped together in themes. Using constant comparison techniques across the transcripts' themes looking for similarities and differences, the themes will be reviewed and refined. Extracts from the data will be used in the final report. Reflexive research memos will be used as an audit trail of the analysis procedure (39). A second qualitative researcher will conduct an independent analysis of a subset of six transcripts before the researchers meet to discuss and agree the findings. Findings will also be presented to the study's patient advisory group for discussion. Any significant differences of opinion will be discussed with the Chief Investigator. A model may be developed to explain the

For peer review only

For peer review only - http://bmjopen.bmj.com/site/about/guidelines.xhtml

factors affecting recruitment and retention to the trial to inform development of the research processes required in any future full trial.

Economic data and analyses

A definitive study will include a within trial economic evaluation to compare costs and health outcomes of ICS versus donor blood within the time frame of the study and a decision analytic model to extrapolate any future health benefits and costs to the lifetime of the participant. The evaluations will primarily be in relation to quality adjusted life years and will take a health and social perspective on costs, in accordance with NICE guidelines (37). Secondary analyses will take place in relation to important clinical outcomes of interest for the definitive trial such as deaths averted and disease-free progression. This study aims to test the feasibility of collecting enough resource use and outcome data to perform the future economic evaluations.

Data collection tools will be prepared and refined with a view to undertaking the two planned economic evaluations within the future study. These evaluations will take on a health and social care payer perspective. Should participant-reported resource use data allow, the future within-trial economic evaluation will take on a societal perspective on costs in secondary analyses, to further capture the burden to participants, carers, and society. The parameters for the lifetime economic decision model (costs, outcomes, and probabilities of outcomes to occur) will be informed by the within trial economic results. If feasible, costs from a societal perspective may be included in the lifetime economic decision model as well.

Resources will be collected from several sources. In the immediate post-operative period, research nurses will record resources pertaining to the participant's surgery and subsequent hospital stay. Where possible, research staff will also review participants' medical notes at 4.5 months post-operatively to collect hospital contacts following initial discharge (i.e. re-hospitalisations, outpatient and emergency visits). Participant-completed resource use questionnaires will be administered at both six weeks and 4.5 months post-operatively (where the trial schedule allows) to collect other

For peer review only

For peer review only - http://bmjopen.bmj.com/site/about/guidelines.xhtml

resources used. These questionnaires will be delivered by post and include questions related to in-patient and out-patient hospital visits; community based services such as General Practice doctor and nurse contacts, physiotherapy, occupational therapy and other community contacts; use of personal social services such as home care workers and social workers; privately paid therapies and expenses; time off work and lost leisure; and informal care required from family and friends. Completion rates, missing data and the method of administering questionnaires will be reviewed to identify potential problems with data collection methods and to seek solutions to minimise participant/staff burden if required. We will report frequency, mean, and standard deviation of resources used by trial arm to explore potential cost-drivers for the main study.

The EuroQoL EQ-5D questionnaire will capture generic quality of life differences between the trial arms. In a recent study of EQ-5D valuation sets, the 3L and 5L versions of the EQ-5D produced substantially different estimates for cost-effectiveness (40) and prompted NICE to issue a position statement in August 2017 to recommend the future use of the 3L version (41). In this study, we will use the mapped utility scores from the 3L to the 5L version using the Van Hout algorithm (42) for the UK population, as recommended by the NICE statement. We expect to use the 3L version in the future study and not proceed with the study of the distribution properties produced by the 5L version scores in this feasibility study.

Patient and Public Involvement and Engagement

The study has benefitted from its inception from an enthusiastic patient advisory group. The aim of PPI in the study is to ensure that the trial is equitable and acceptable to the women taking part by embedding the women's experiential expertise of cancer throughout the trial design and processes. The group comprises six women aged between 50-80 years, who have experienced a cancer diagnosis and are living in Cornwall. However, one member is formerly from Gateshead, where she was treated for her cancer, so is able to bring her experience of the patient pathway to inform the trial processes across the sites. Another member and co-applicant is the founder of PANTS cancer charity in Cornwall.

For peer review only - http://bmjopen.bmj.com/site/about/guidelines.xhtml

The PPI work is undertaken using a predominately collaborative approach with engagement functions embedded within it. The members worked with the research team on the research design and in particular the patient approach, providing input into the grant application, language, content and layout of the participant documentation. The group have worked on the qualitative interview topic guide content and are also working with the qualitative researchers on analysis of the participant interview transcripts. The members are fully integrated into the team and regularly attend the trial management meetings, as well as providing advice and suggesting solutions to problems encountered during the trial.

The members will attend patient and public events and conferences to engage with other members of the public and professionals and share their experience of supporting and being part of the design and management of research. They will also work together with the wider research team to prepare a lay summary of the findings and on other communications such as website, Twitter and Facebook articles.

All members of the research team contribute to the training and support of the PPI members. The mechanisms to achieve these are multifactorial and include specific discussion around methodology and trial processes in PPI meetings, explaining the terminology in lay language, providing information, such as the Involve jargon buster sheet and conducting workshops for specific tasks (e.g. poster development), as well as signposting to other resources such as the Involve website.

For peer review only

ETHICS AND DISSEMINATION

The results of this feasibility study will be published in peer-reviewed journals and presented at relevant national/international conferences and to patient groups. Participants of the trial will be sent a summary of the findings and these will also be disseminated via the pantscancer.org charity, Target Ovarian Cancer charity and participating NHS Trusts' websites.

DISCUSSION

For peer review only - http://bmjopen.bmj.com/site/about/guidelines.xhtml

Research has shown that donor blood transfusions have been associated with poorer outcomes including increased mortality, wound, pulmonary and renal complications; this has been ascribed to transfusion-induced immune modulation (TRIM) (9) which is a transient depression of the immune system following transfusion with blood products. The Cochrane meta-analysis of randomised trials estimated perioperative allogeneic blood transfusion to be associated with increased risk of recurrence with odds ratio of 1.42 (95% CI, 1.20 to 1.67) in surgery for colorectal cancer (43). Long-term results from a clinical trial suggest that this effect of allogeneic blood transfusion is persistent (44, 45). This led to the suggestion of introducing measures that would help limit the use of allogeneic blood transfusion (12).

Patient blood management is an evidence-based patient-tailored approach aimed at reducing the need for allogeneic blood transfusion by managing anaemia, perioperative blood conservation, surgical haemostasis, and drug use (46). Perioperative blood conservation measures include interventions such as the administration of agents to diminish blood loss (e.g. tranexamic acid, fibrin sealant), agents that promote red blood cell production (e.g. erythropoietin) and techniques for reinfusing a patient's own blood including cell salvage (28). Previous randomised and non-randomised studies have provided evidence that the use of intraoperative cell salvage can reduce the need for allogeneic blood transfusion (ABT) (9). A systematic review of 75 randomised trials highlighted that salvaged blood reinfusion reduced the rate of exposure to ABT by 38% (relative risk, 0.62; 95% confidence interval [95% CI], 0.55-0.70) (47). However, concern exists that blood collected by intraoperative cell salvage might result in reinfusion of tumour cells and subsequent distant metastases thus limiting the use of cell salvage across oncological specialties. However, in patients undergoing surgery for a gynaecological malignancy, the use of a leucocyte depletion filter was shown to be effective in eliminating viable nucleated malignant cells from the returned blood during collection, processing, and leukofiltration (27). Similarly, in vitro work shows that depletion filters are highly efficient at removing malignant cells, leading to removal rates of between 80 and 100% (25, 26).

For peer review only - http://bmjopen.bmj.com/site/about/guidelines.xhtml

Patients with primary or metastatic cancer are known to have CTCs in the blood. The concentration of CTCs varies widely depending on tumour type and stage of disease (31). There is evidence from a range of different cancer surgeries that operative manipulation of tumour during surgery leads to peripheral blood concentrations of malignant cells many times higher than could be attained with cell salvage alone (31, 32, 48).

There is emerging evidence suggesting that far from compromising outcomes, intraoperative autologous transfusion is associated with improved outcomes in surgery for other gynaecological cancers such as cervical cancer. Several studies in early stage (I-IIA) cervical cancer patients report that intraoperative autologous transfusion significantly reduces the need for donor blood transfusion, without compromising survival or post-operative complication rates (30). In addition, no distant recurrences have been reported (30). However, most of the evidence on the use of salvaged blood in cancer surgery is based on retrospective and observational studies. These studies are insufficient to draw any definitive conclusions regarding adverse events related to a particular intervention in the presence of multiple confounding factors. Therefore in order to mitigate for confounding factors a large well-designed randomised controlled trials are required (49). Our trial provides new evidence in the use of cell salvage in ovarian cancer surgery and will add to a more general evidence base informing the use of ICS in other areas, in particular other cancers.

For peer review only

Conflict of interest statement

The authors declare no conflict of interest.

This work was supported by the National Institute for Health Research (NIHR) Research for Patient Benefit (RfPB) programme, grant number PB-PG-1014-35005.

Contributorship statement

All authors except NF and JP were co-applicants on the NIHR RfPB grant application and as such were involved in the design of this feasibility study. All authors contributed to successive drafts of this paper.

For peer review only - http://bmjopen.bmj.com/site/about/guidelines.xhtml

KG is the Chief Investigator, provided clinical expertise and was responsible for conception and design of the study as well as drafting and revising of the article.

NF was responsible for the first draft of this paper.

CP contributed to study design.

AB contributed to study design and trial management.

PE is the trial statistician and provided expertise in the overall design of the trial.

JF provided expertise in cell salvage and drafted the cell salvage protocol.

AL provided clinical expertise and helped with design of the study.

EM was responsible for the design and analysis of the economic evaluation component.

JP contributed to study design and coordinated the PPI input.

CR provided anaesthetics and cell salvage expertise

JV is the trial manager, responsible for overseeing the day-to-day running of the trial.

JW was responsible for the design and conduct of the qualitative study.

Acknowledgements

The authors are grateful for the support of the study sponsor (Royal Cornwall Hospitals NHS Trust) and the South West Peninsula NIHR Clinical Research Network. We are also indebted to the members of our PPI group for their continued support of the trial. In addition, the authors would like to thank the following members of the TIC TOC trial team: Mr S Chatopadhyay, Mr G Hughes, Mr R Naik and Dr P Ricketts.

DH disclaimer

The views expressed are those of the author(s) and not necessarily those of the NHS, the NIHR or the Department of Health.

For peer review only

For peer review only

For peer review only - http://bmjopen.bmj.com/site/about/guidelines.xhtml

References

1. Cancer Research UK. Ovarian cancer incidence statistics http://www.cancerresearchuk.org/cancer-; 2014.

2. National Cancer Intelligence Network (NCIN). Overview of Ovarian Cancer in England: Incidence, Mortality and Survival. Trent Cancer Registry November 2012 [Internet]. 2012.

3. Schwartz PE. Cytoreductive surgery in the management of ovarian cancer Oncology 22: 1025-33. 2008;22(9):1025-33 .

4. Peiretti M ZV, Aletti GD, Bocciolone L, Colombo N, Landoni F, Minig L, Biffi R, Radice D, Maggioni A. Role of maximal primary cytoreductive surgery in patients with advanced epithelial ovarian and tubal cancer: Surgical and oncological outcomes. Single institution experience. Gynecol Oncol. 2010;119(2):256-64.

5. Prescott LS AT, Brown AJ, Taylor JS, Munsell MF, Sun CC, et al,. Perioperative blood transfusion in gynecologic oncology surgery: analysis of the National Surgical Quality Improvement Program Database. Gynecologic oncology. 2015;136(1):65-70.

6. Warner LL DS, Martin JR, Lemens MA, McGree ME, Weaver AL, et al,. The impact of perioperative packed red blood cell transfusion on survival in epithelial ovarian cancer. . Int J Gynecol Cancer 2013;23:1612–9.

7. Ferraris VA DD, Saha SP, Austin PC, Zwischenberger JB. Surgical Outcomes and Transfusion of Minimal Amounts of Blood in the Operating Room. JAMA Surgery. Jan 2012, Vol 147, No. 1 JAMA Surgery. 2012;147(1):49-55.

8. PHB Bolton-Maggs (Ed) DP, A Watt, D Thomas and H Cohen on behalf of the Serious Hazards of Transfusion (SHOT) Steering Group. The 2012 Annual SHOT Report (2013)

9. Blajchman MA, and Eleftherios C. Vamvakas. "Transfusion-Related Immunomodulation." Practical Transfusion Medicine. Third Edition (2009): 98-106. 2009.

10. Goubran H SD, Radosevic J, Burnouf T, Seghatchian J,. Transfusion-related immunomodulation and cancer. Transfus Apher Sci. 2017;56(3):336–40.

11. Ashworth A KAA. Cell salvage as part of a blood conservation strategy in anaesthesia. Br J Anaesth. 2010;105(4):401-16.

12. The Association of Anaesthetists of Great Britain and Ireland (AAGBI). The Association of Anaesthetists of Great Britain and Ireland (AAGBI) Safety Guideline—Blood Transfusion and the Anaesthetist: Intra-operative Cell Salvage2009.

13. Krajewski K AR, Pung N, Wald S, Lazareff J, Kawamoto HK, Bradley JP. Successful blood conservation during craniosynostotic correction with dual therapy using Procrit and cell saver. J Caraniofac Surg. 2008;19:101-5.

14. Bridgens JP EC, Dobson PMS, Hamer AJ. Intraoperative red blood-cell salvage in revision hip surgery. J Bone Joint Surg 2008;89:270-5.

15. Colwell CW Jr. BE, West C, Hardwick ME, Morris BA. Erythrocyte viability in blood salvaged during total joint arthroplasty with cement. J Bone Joint Surg 2002;84:23-5.

16. Williamson KR TH. Intraoperative blood salvage: a review. Transfusion. 1991;31(7):662-75.

17. Akbulut S KC, Yilmaz M, Ince V, Ozgor D, Karabulut K, Eris C, Toprak HI, Aydin C, Yilmaz S. Effect of autotransfusion system on tumor recurrence and survival in hepatocellular carcinoma patients World J Gastroenterol. 2013;19(10):1625–31.

18. Wang G BD, Martin J, Cheng D The efficacy of an intraoperative cell saver during cardiac surgery: a meta-analysis of randomized trials. Anesth Analg. 2009;109(2):320-30.

19. Marković M DL, Savić N, Sindjelić R, Ille T, Dragas M. Intraoperative cell salvage versus allogeneic transfusion during abdominal aortic surgery: clinical and financial outcomes. Vascular. 2009;17(2):83-92.

20. Breakwell LM GC, Dobson P. T,. The efficacy of autologous blood transfusion in bilateral total knee arthroplasty. Knee. 2000;7(3):145–7.

For peer review only - http://bmjopen.bmj.com/site/about/guidelines.xhtml

21. DeAndrade D WJ, Triulzi DJ, Alarcon L, Wisniewski MK, Dyga R, et al,. Very low rate of patient-related adverse events associated with the use of intraoperative cell salvage. Transfusion. 2016 Nov;56(11):2768–72. Transfusion. 2016;56(11):2768–72.
22. Hansen E WN, Kneuchel R, Ruschoff J, Hofstaedter F. Hansen E, Wolff N, Kneuchel R, Ruschoff J, Hofstaedter F. Tumor calls in blood shed from the surgical field. Arch.Surg. 1995; 130(4) 387-393. Tumor calls in blood shed from the surgical field. 1995;130(4):387-93.
23. Kumar N ZA, Kantharajanna SB, Khoo BL, Lim CT, Thiery JP,. Metastatic efficiency of tumour cells can be impaired by intraoperative cell salvage process: truth or conjecture? . Transfus Med. 2017;Suppl 5:327-34.
24. Motoyama S SR, Kamata S, Kitamura M, Okuyama M, Imano H, Nakamura M, Suzuki H, Omokawa S, Motohashi Y, Ogawa J. Survival advantage of using autologous blood transfusion during surgery for esophageal cancer. Surg Today. 2002;32(11):951-8.
25. Karczewski DM LM, Glaves D. The efficiency of an autotransfusion system for tumor cell removal from blood salvaged during cancer surgery. Anesth Analg 1994;78(6):1131-5.
26. Fruhauf NR DO, Kaudel CP, Kasimir-Bauer S, Oldhafer KJ. Filtration of malignant cells: tumour cell depletion in an ex vivo model using a leukocyte adhesion filter. Perfusion 2001;16:Suppl:51-5.
27. Catling S WS, Freites O, Rees M, Davies C, Hopkins L. Use of a leucocyte filter to remove tumour cells from intra-operative cell salvage blood. Anaesthesia 2008;63(12):1332-8.
28. Zaw AS BKS, Kumar N,. Is Autologous Salvaged Blood a Viable Option for Patient Blood Management in Oncologic Surgery? . Transfusion Medicine Reviews. 2017;31(1):56-61.
29. Connor JP MP, Alagoz T, Anderson B, Bottles K, Buller RE. Intraoperative autologous blood collection and autotransfusion in the surgical management of early cancers of the uterine cervix. Obstet Gynecol. 1995;86(3):373- 8.
30. Mirhashemi R AH, Deepika K, Estape R, Angioli R, Martin J, Rodriguez M, Penalver MA. The impact of intraoperative autologous blood transfusion during type III radical hysterectomy for early-stage cervical cancer. Am J Obstet Gynecol 1999;181(6):1310-5; discussion 5-6.
31. AllardWJ MJ, MillerMC, Repollet M, Connelly MC, Rao C, et al,. Tumor cells circulate in the peripheral blood of all major carcinomas but not in healthy subjects or patients with nonmalignant diseases. Clin Cancer Res. 2004;10:6897–904.
32. Oefelein MG KK, Herz B, Blum MD, Holland JM, Keeler TC, Cook WA, Ignatoff JM. Molecular detection of prostate epithelial cells from the surgical field and peripheral circulation during radical prostatectomy. J Urol 1996;155(1):238-42.
33. Allan AL KM. Circulating tumor cell analysis: technical and statistical considerations

for application to the clinic. J Oncol 2010;2010:426218.
34. Galaal K BA, Smits A, Lopes AD,. Intraoperative Cell Salvage in Surgery for Ovarian Cancer: Time for Action. . Medical Journal of Obstetrics and Gynecology 2014;2(2):1035.
35. Society of Gynecologic Oncology. FIGO ovarian cancer staging: https://www.sgo.org/wp-content/uploads/2012/FIGO-Ovarian-Cancer-Staging_1.10.14.pdf; 2014 [
36. Oken MM, Creech, R.H., Tormey, D.C., Horton, J., Davis, T.E., McFadden, E.T., Carbone, P.P. Toxicity And Response Criteria Of The Eastern Cooperative Oncology Group. Am J Clin Oncol 1982;5:649-55.
37. Braun V CV. Using thematic analysis in psychology. Qualitative Research in Psychology. 2006;3:77-101.
38. Green J TN. 2004. Qualitative Health Methods for Health Research. London: Sage Publications.
39. Liamputtong P ED. Qualitative Research Methods. Second Edition ed. Oxford: Oxford University Press; 2005. In: Press OU, editor. Oxford:2005.
40. Wailoo A HAM, Grimm S et al. Comparing the EQ-5D-3L and 5L versions. What are the implications for cost effectiveness estimates? NICE Decision Support Unit report 2017 6th July 2017.

For peer review only - http://bmjopen.bmj.com/site/about/guidelines.xhtml

41. https://www.nice.org.uk/Media/Default/About/what-we-do/NICE-guidance/NICE-technology-appraisal-guidance/eq5d5l_nice_position_statement.pdf.
42. Van Hout B JM, Feng Y et al,. Interim scoring for the EQ-5D-5L: Mapping the EQ-5D-5L to EQ-5D-3L value sets. Value in Health. 2012;15:708-15.
43. Amato A PM. Perioperative blood transfusions and recurrence of colorectal cancer. Cochrane Database of Systematic Reviews 2006, Issue 1. Art. No.: CD005033. DOI: 10.1002/14651858.CD005033.pub2. Cochrane Database of Systematic Reviews. 2006(1).
44. Linder BJ FI, Cheville JC, Tollefson MK, Thompson RH, Tarrell RF, Thapa P, Boorjian SA. The Impact of Perioperative Blood Transfusion on Cancer Recurrence and Survival Following Radical Cystectomy Eur Urol. 2013;63(5):839–45
45. Harlaar JJ GM, Hop WC, Lange JF, Busch OR, Jeekel H Blood Transfusions and Prognosis in Colorectal Cancer: Long-term Results of a Randomized Controlled Trial Ann Surg. 2012;256:681-6
46. Spahn DR SA, Hofmann A,. The chiasm: transfusion practice versus patient blood management. Best Pract Res Clin Anaesthesiol 2013;27:37-42.
47. Carless PA HD, Moxey AJ, O'Connell D, Brown T, Fergusson DA. Cell salvage for minimising perioperative allogeneic blood transfusion. Cochrane Database of Systematic Reviews 2010(4):Art. No: CD001888.
48. Yamaguchi K TY, Aoki S, FutamuraM, Saji S,. Significant detection of circulating cancer cells in the blood by reverse transcriptase-polymerase chain reaction during colorectal cancer resection. Ann Surg 2000;232:58-65.
49. Yazer MH TD. Things aren't always as they seem: what the randomized trialsof red blood cell transfusion tell us about adverse outcomes. Transfusion 2014;54:3243–6 [quiz 2].

For peer review only

Legend

Table 1 Trial schedule

Figure 1 Summary of trial design

Appendix 1 Participant Information Sheet

Appendix 2 Informed Consent Form

Appendix 3 FIGO Ovarian Cancer Staging

Appendix 4 Definition of surgical site infection

Appendix 5 Topic guide for participant interviews

For peer review only

For peer review only - http://bmjopen.bmj.com/site/about/guidelines.xhtml

Figure 1: Summary of trial design

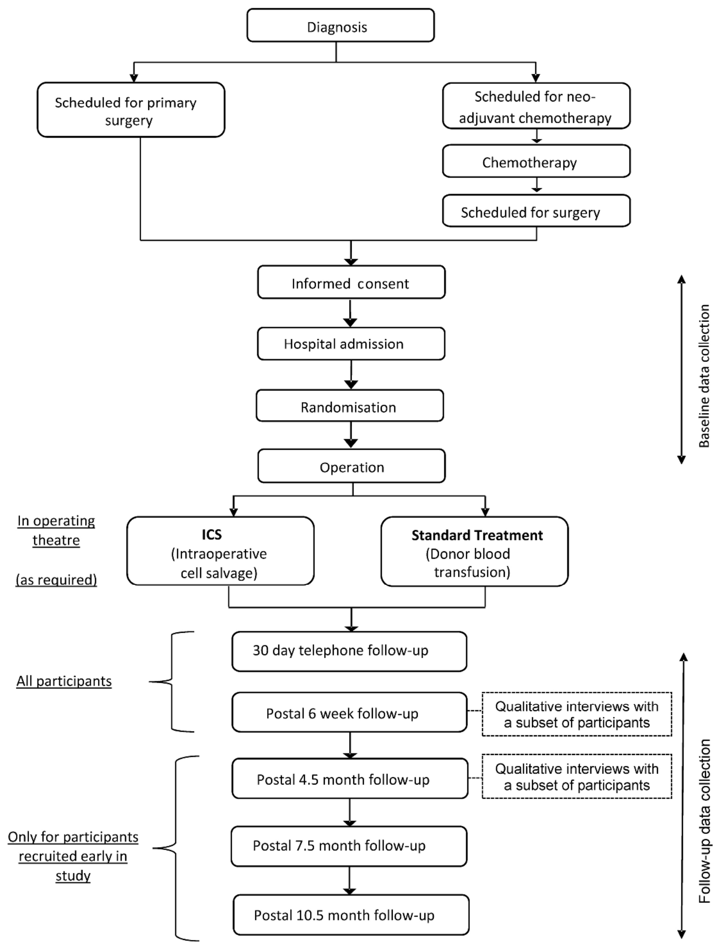

TIC TOC protocol paper Table_figure_K Galaal_31.07.18 ISRCTN19517317 Page **2** of **2**

297x420mm (300 x 300 DPI)

Appendix 1: Participant Information Sheet

TIC TOC

Trial of intraoperative cell salvage versus transfusion in ovarian cancer – a feasibility study (the TIC TOC study)

Participant Information Sheet

We'd like to invite you to take part in our research study

- Before you decide whether to take part it is important for you to understand why the research is being done and what it would involve for you.
- Please take time to read the following information carefully. Discuss it with your family, friends or your family doctor (GP) if you wish.
- You are free to decide whether or not to take part in this study. If you choose not to take part, this will not affect the care you get from your doctors.
- Please ask us if anything is not clear, or if you would like [illegible]

Important information about this study

- This is a **feasibility study.** A feasibility study may be carried out before a main study in order to answer the question "Can this study be done?"
- The aim of this study is to find out whether we can successfully plan and carry out a larger study in the future.
- If you take part in this study you will be randomly allocated to receive **either** a reinfusion (return) of your own blood (called Intraoperative Cell Salvage) **or** a transfusion of donated blood (standard blood transfusion), if there is enough blood loss during your forthcoming operation.
- Your care and medications will continue as normal.
- The study involves completing some follow-up questionnaires which will be sent to you by post.

Key contents

	Page
What is the purpose of the study?	2
Donor blood transfusion	2
Intraoperative cell salvage (ICS)	2
Why have I been approached?	2
What does the study involve?	2
Do I have to take part?	4
Study flowchart	5
What are the possible risks?	6
General information	7

If you have any questions about this study please contact:

Your research nurse:

<Enter local contact name>

>Enter local contact details<

For peer review only - http://bmjopen.bmj.com/site/about/guidelines.xhtml

What is the purpose of the study?

During any surgical operation it is common for there to be some loss of blood. Sometimes this is significant enough for the patient to need a blood transfusion. Giving someone a blood transfusion involves transfusing donated blood from an anonymous healthy donor.

For a long time, donor blood has been the only choice available for blood transfusion. Although this is a very safe procedure in the UK it is not completely without risk. There is now a technique available in which blood lost by a patient during surgery is collected, washed and given back to the same patient. The technical name for this is **Intraoperative Cell Salvage** (ICS).

Donor blood transfusion

- Blood from an anonymous donor
- Washed and filtered in the blood bank

ICS blood reinfusion (ICS blood return)

- Patient's own blood
- Washed and filtered during the operation

Surgery is one of the main treatments for ovarian cancer. ICS blood return is already used successfully in other types of surgery and is being used in cancer surgery including some ovarian cancer operations. There is some evidence that using ICS blood return instead of donor blood transfusion promotes better recovery for patients after surgery. However, we do not know which method is better for patients undergoing ovarian cancer surgery. We also do not know which method is better value for money. This study will help us to answer those questions.

Why have I been approached to take part?

You are being invited to take part because you are due to have surgery for ovarian cancer, or suspected ovarian cancer, at one of the four participating hospitals and have been identified as being potentially suitable for the study. You cannot take part if: -

- You have any other diagnosed cancer
- You are pregnant
- You have any disease of the red blood cells such as sickle cell or thalassaemia
- You are unwilling to accept donor blood (e.g. on religious grounds)

What does the study involve?

This study will involve 60 women in total. Half of the women taking part in the study will be allocated to receive a donor blood transfusion. The other half will be allocated to receive an ICS blood return. All women will receive a brief telephone check-up by a research nurse approximately one month after surgery, and will also be asked to complete up to four study follow-up questionnaire booklets by post. There are more details about this on page 4. There are **no** extra hospital visits required.

For peer review only - http://bmjopen.bmj.com/site/about/guidelines.xhtml

What will happen to me if I agree to take part in the study?

If you agree to take part in the study after considering the information provided, you will be asked to sign a consent form before any study procedures are completed. Consent will usually be taken when you attend the routine pre-operative assessment clinic. A research nurse will then review your past and current health status and you will be asked to complete a questionnaire booklet about your general health and wellbeing, and how your illness is affecting your daily life. When you have your operation, you may be given blood replacement by either a donor blood transfusion (if there is enough blood loss) or you will be given an ICS blood return. The blood replacement you receive will depend on which method you are allocated. Some women may not require any blood replacement at all.

Who decides which type of blood replacement I receive?

If you consent to take part in the study, you will be allocated at random (by chance – like tossing a coin) by computer to receive either ICS blood return during your operation, or, if replacement blood is needed, a donor blood transfusion. This is called **randomisation**. All other aspects of your operation and care will be exactly the same as if you had decided not to be involved in the study. During the study, you will not be told which type of blood replacement you have received but you can find this out when the study has ended (more information is provided on page 7). In accordance with standard practice, women who receive donor blood will only be given a blood transfusion if there is enough blood loss during the operation. Women allocated to ICS blood will be given ICS blood return, even if there is only a little blood loss during the operation.

What if I am to receive chemotherapy before my surgery?

Your surgeon / doctor at the hospital will inform you if you require chemotherapy prior to your surgery and will provide you with the information you need. This is called neo-adjuvant chemotherapy. You can still take part in the study if you need chemotherapy first.

What happens if I do not require blood replacement?.

Women undergoing surgery for ovarian cancer (or suspected cancer) do not always require blood replacement, so some women taking part in this study will not receive blood of any sort. If you are one of these women, you are still very important to the study and we would still like to collect the same information from you.

For peer review only - http://bmjopen.bmj.com/site/about/guidelines.xhtml

Blood replacement after your operation

Sometimes it is usual for some patients to require blood replacement after their operation, either in the recovery unit or on the ward. If you need blood after surgery, this will be a donor blood transfusion regardless of whether you were in the group that received donor blood or your own ICS blood during your operation. This is because the ICS machine cannot be used on the hospital ward. If you require a donor transfusion after surgery your participation in the study will still be valid and it is important that your information is still l included in the study analysis.

What happens after I've had my operation?

Participation in this study will not interfere with your usual care and recovery from surgery and should not delay your discharge home. If you take part in this study, the research nurse will record some details about your operation, recovery and whether or not you needed blood replacement. Any routine hospital follow-up visits will continue as usual.

Approximately **30 days** after your operation the research nurse will telephone you to ask about your general health and wellbeing since you were discharged from hospital. **Six weeks** after your operation you will be sent a questionnaire booklet to complete - the length of the booklet depends upon your final diagnosis. Your completed questionnaire booklet should be returned in the pre-paid envelope provided.

People who are recruited in the early stages of the study will be sent repeat questionnaire booklets, at three month intervals, as time allows (see flowchart on page 5). At these time points we will also ask you about any contacts you may have had with your hospital, GP, district nurse or other services since discharge from hospital. The questionnaire booklet should take no longer than 30 minutes to complete in total.

Do I have to take part?

No. Taking part in this study is entirely voluntary and it is up to you to decide whether or not to take part. If you do decide to take part you will be asked to sign a consent form, but you are still free to withdraw at any time in the future and without giving a reason. You will be given a copy of this information sheet and a copy of your signed consent form to keep. If you decide not to take part, or you withdraw from the study at any point, your medical care will not be affected in any way.

What will happen to me if I do NOT take part in the study?

If you decide this study is not for you at this time, you will be given the usual care and treatment offered at your hospital. If you need blood replacement during your operation, you may be offered ICS blood return as part of usual care (depending on the extent of your surgery

For peer review only - http://bmjopen.bmj.com/site/about/guidelines.xhtml

and the expected blood loss) even if you do not take part in the study. Usual care varies between hospitals because there is currently no evidence to show that receiving your own blood (ICS blood) is better or worse than donor blood. Currently ICS blood reinfusion is offered for ovarian cancer surgery in two of the hospitals participating in this study (Truro and Plymouth) but is not routinely offered in Gateshead or Leicester.

Study flowchart

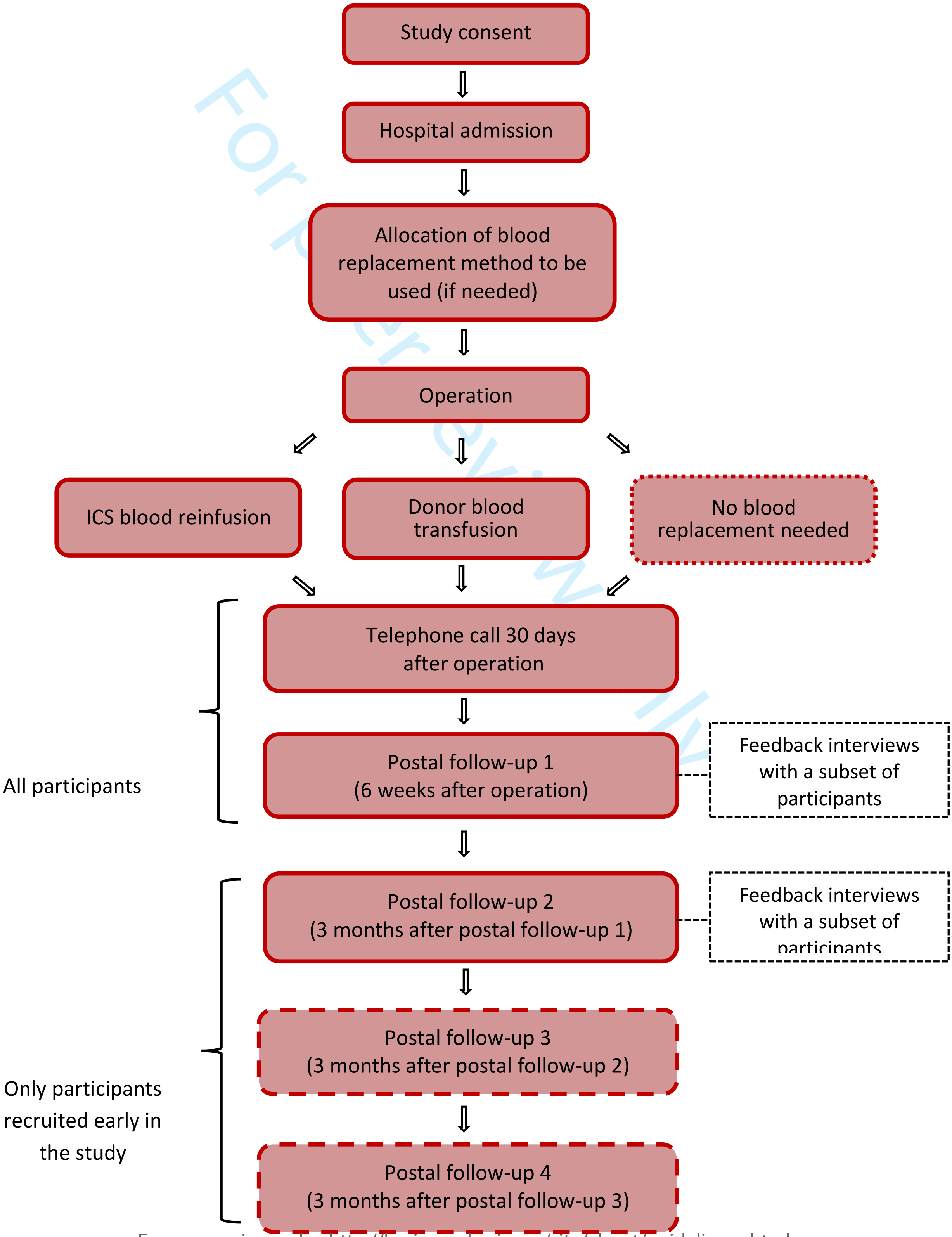

For peer review only - http://bmjopen.bmj.com/site/about/guidelines.xhtml

What are the possible benefits of taking part?

You may or may not benefit directly from this study but by taking part you will be contributing to a study which could potentially bring future benefit to women with ovarian cancer around the world. We don't know whether one method of blood replacement compared with the other improves recovery after surgery for ovarian cancer, but we hope that this study will help to answer that question.

What are the possible disadvantages and risks of taking part?

If you agree to take part in this study you will be asked to complete some questionnaire booklets, as described on page 4. These will take 15-30 minutes on each occasion and you will be asked to complete these two times (and up to a maximum of five times) over several months.

You may or may not receive blood replacement if you take part in this study. The anaesthetist and surgeon will decide whether you require additional blood during your operation as part of your usual care. If you need blood replacement during surgery, you may be given a donor blood transfusion or you may be given ICS blood return.

With any donor blood transfusion there is a possibility of side effects, including an increased risk of wound or other infections, lung and kidney problems, and a risk of receiving the wrong blood type in error. Such events and adverse transfusion reactions are rare. Donor blood transfusion has been used widely for many years and is considered a safe way of delivering blood to patients.

Intraoperative cell salvage (ICS) has been used in cancer operations, including ovarian cancer. Its use has been limited because of the theoretical risk (i.e. based on theory rather than experience) of reintroducing cancer cells into the bloodstream. However, the risk of cancer cells entering the bloodstream is low as far as current evidence shows, because a special filter is used which can remove any active cancer cells from the returned blood.

It is possible that the ICS technique may cause a temporary lowering of blood pressure but this is monitored continuously during the operation and any problem can be quickly corrected. There are no other documented problems with using ICS known to date.

For peer review only - http://bmjopen.bmj.com/site/about/guidelines.xhtml

What happens when the research study stops?

Once your participation in the study has ended, your usual care will continue as before. When every woman has completed their involvement in the study, we will prepare the study results (which normally takes several months) which will be available to participants.

If you would like to know whether you received a donor blood transfusion or ICS blood return, your research team will be able to tell you once everyone has completed the study. This is likely to be in the summer of 2018. The study results may be presented at national and international conferences and published in medical journals but you will not be identified in any information included in any presentation or publication.

General information about this study

What if relevant new information becomes available?

A special committee will be set up to look at all the information collected during the course of the TIC TOC study and will ensure that any study-related issues of concern are investigated. If the study is stopped for any reason, you will be told why. If any new information about ICS blood return becomes available which might affect your participation in the study, you will be informed.

What happens if I don't want to carry on with the study?

You are free to withdraw from the study at any time, without giving any reason, and without your medical care or legal rights being affected. If you want to withdraw from the study before you have your surgery, you must do this before you are given any anaesthetic. If you decide to withdraw from the study at any stage, we may still use information collected about you unless you ask us not to.

What if there is a problem?

Complaints: If you have a concern about any aspect of this study, please speak to someone in your research team who will do their best to answer your questions. If you remain unhappy and wish to complain formally, you can do this through your local NHS complaints procedure. The NHS has a Patient Advice and Liaison Service (PALS) for information and support, which can be found at your local hospital <Enter local PALS contact details>. You can also contact the department responsible for overseeing the study: Research, Development and

For peer review only - http://bmjopen.bmj.com/site/about/guidelines.xhtml

Innovations Manager, Knowledge Spa, Royal Cornwall Hospitals NHS Trust, Truro TR1 3HD. Tel: 01872 246424.

Harm: We don't expect any harm to come to you as a result of participating in this study. If you are harmed and this is due to someone's negligence, then you may have grounds for legal action for compensation against your hospital's Trust but you may have to pay your legal costs.

There are no special compensation arrangements in place. The normal NHS complaints mechanisms will still be available to you; your PALS service will be able to advise you.

Private insurance policies: Please note that it is your responsibility to check if taking part in this study affects the terms and conditions of any private insurance policies that you hold.

Will the information collected during the study be kept confidential?

All information collected about you whilst taking part in this study will be kept strictly confidential and will be collected and stored for five years after the study is complete, in accordance with the Data Protection Act (1998). You will be given a unique study number which your study information will be labelled with, along with your initials, so that you cannot be identified (known as pseudonymised or de-identified data). This study information will be stored and analysed at Plymouth University. Only members of the research team and the Peninsula Clinical Trials Unit (PenCTU) at Plymouth University will have direct access to the study information. Paper-based information will be stored in locked filing cabinets within a locked office in the PenCTU. Information kept on computers will be stored securely on a system maintained by Plymouth University. Copies of the study information will be held securely at your local hospital but will not contain any details that could identify you.

Authorised people from your NHS Trust, the PenCTU and study organisers may need to review your medical records to check that the study is being carried out correctly. All will have a duty of confidentiality to you as a research participant. As part of the consent process, you will be asked to consent to your contact details (name, address, telephone number) being provided to the PenCTU and the TIC TOC researcher based at Royal Cornwall Hospitals NHS Trust, to enable collection of some information by post. At the PenCTU, these details will be stored separately from the de-identified study information also held.

For peer review only - http://bmjopen.bmj.com/site/about/guidelines.xhtml

Will the study information help with other research projects?

It is important that good quality research data can be shared with others in order to advance clinical research and to benefit patients in the future. After the end of the study, de-identified information collected during the study will be made available to other researchers under an appropriate data sharing agreement, but it will not be possible to identify you personally from any information shared.

This is a feasibility study, so the aim is to test the processes required for a large study. This study will provide us with the necessary information to help us to learn what to consider when designing a main study in the future. Ultimately, the main study will assess whether ICS or donor blood transfusion is associated with better outcomes for patients having ovarian cancer surgery.

Involvement of your General Practitioner/ Family Doctor (GP)

Your general practitioner will be informed of your participation in this study.

Who is organising and funding the study?

The study is being led by Miss Khadra Galaal, Consultant Gynaecological Oncologist at the Royal Cornwall Hospitals NHS Trust (RCHT). The study is funded by the National Institute for Health Research (NIHR) Research for Patient Benefit grant scheme Ref: PB-PG-1014-35005. The study will be managed by the Peninsula Clinical Trials Unit at Plymouth University and sponsored (overseen) by RCHT.

Who has reviewed the study?

All NHS research is looked at by an independent panel (Research Ethics Committee). This study has been reviewed and been given a favourable opinion by the <Enter name> Research Ethics Committee.

Thank you for taking the time to read this information sheet and for considering taking part in the TIC TOC study.

For peer review only

TIC TOC

TO BE PRINTED ON RELEVANT NHS TRUST HEADED PAPER

Appendix 2: Informed consent form

PARTICIPANT CONSENT FORM

A randomised, controlled feasibility trial of intraoperative cell salvage versus donor blood transfusion in ovarian cancer surgery

Principal Investigator: <Insert PI's name> **Participant Study Number:** | | | | |

Please initial each box

1. I confirm that I have read and understood the information sheet (version 2.0, dated 09 October 2017) for the above study. I have had the opportunity to consider the information and ask questions and I have had my questions answered satisfactorily. ☐
2. I understand that my participation in this study is voluntary and that I am free to withdraw at any time without giving any reason and without my medical care or legal rights being affected. ☐
3. I agree that my name, address and telephone number can be given to and stored by the Peninsula Clinical Trials Unit at Plymouth University to enable collection of study information by post. ☐
4. I understand that relevant sections of any of my medical notes and information collected during the study may be looked at by responsible individuals from my local NHS Trust, the Peninsula Clinical Trials Unit and the regulatory authorities, where it is relevant to my taking part in this research. I give permission for these individuals to have access to my records. ☐
5. I understand that an anonymised copy of this consent form will be sent to the Peninsula Clinical Trials Unit to confirm my agreement to participate. ☐
6. I understand that the information collected about me may be shared anonymously with other researchers to support future research studies. I cannot be personally identified from this. ☐
7. I agree to take part in the TIC TOC study. ☐

____________________ ____________________ ____________________
Print Name (Participant) *Date* *Signature*

____________________ ____________________ ____________________
Print Name (Researcher taking consent) *Date* *Signature*

For peer review only

Appendix 3: FIGO Ovarian Cancer Staging

Effective 1 January 2014

**STAGE I: Tumour confined to ovaries**	
**IA**	Tumour limited to one ovary, capsule intact, no tumour on surface, negative washings
**IB**	Tumour involves both ovaries, otherwise like IA
**IC**	Tumour limited to one or both ovaries
IC1	Surgical spill
IC2	Capsule rupture before surgery or tumour on ovarian surface
IC3	Malignant cells in the ascites or peritoneal washings

**STAGE II: Tumour involves one or both ovaries with pelvic extension (below the pelvic brim) or primary peritoneal cancer**	
**IIA**	Extension and/or implant on uterus and/or Fallopian tubes
**IIB**	Extension to other pelvic intraperitoneal tissues

**STAGE III: Tumour involves 1 or both ovaries with cytologically or histologically confirmed spread to the peritoneum outside the pelvis and/or metastasis to the retroperitoneal lymph nodes**		
**IIIA**	Positive retroperitoneal lymph nodes and/or microscopic metastasis beyond the pelvis	
IIIA1	Positive retroperitoneal lymph nodes only	
	IIIA1 (i)	Metastasis ≤ 10mm
	IIIA1 (ii)	Metastasis > 10mm
IIIA2	Microscopic, extrapelvic (above the brim) peritoneal involvement ± positive retroperitoneal lymph nodes	
IIIB	Macroscopic, extrapelvic, peritoneal metastasis ≤ 2cm ± positive retroperitoneal lymph nodes. Includes extension to capsule of liver/spleen	
IIIC	Macroscopic, extrapelvic, peritoneal metastasis > 2cm ± positive retroperitoneal lymph nodes. Includes extension to capsule of liver/spleen	

**STAGE IV: Distant metastasis excluding peritoneal metastasis**	
IVA	Pleural effusion with positive cytology
IVB	Hepatic and/or splenic parenchymal metastasis, metastasis to extra-abdominal organs (including inguinal lymph nodes and lymph nodes outside of the abdominal cavity)

Other major recommendations are as follows:

- Histologic type including grading should be designated at staging
- Primary site (ovary, Fallopian tube or peritoneum) should be designated where possible
- Tumours that may otherwise qualify for stage I but involved with dense adhesions justify upgrading to stage II if tumour cells are histologically proven to be present in the adhesions.

For peer review only

Appendix 4: Definition of surgical site infection

For the purposes of this study, surgical site infection (48, 49) is defined as an infection that:-

i) occurs within 30 days after the operation and

ii) appears to be related to the operation and

iii) involves deep soft tissues (e.g. fascial and muscle layers) of the incision and at least one of the following:-

a) Purulent drainage from the deep incision but not from the organ/space component of the surgical site

b) A deep incision spontaneously dehisces or is deliberately opened by a surgeon when the patient has at least one of the following signs or symptoms: fever (> 38 C), localized pain, or tenderness, unless site is culture-negative.

c) An abscess or other evidence of infection involving the deep incision is found on direct examination, during reoperation, or by histopathologic or radiologic examination.

d) Diagnosis of a deep incision SSI by a surgeon or attending physician

For peer review only

Appendix 5: Topic guide for participant interviews

First qualitative interview (6 weeks)

**Topic**	**Questions**	**Prompts**
**Opening question**	How are you feeling after your operation? Tell me a bit about yourself?	Role in life – past or present employment<br><br>Family<br><br>Be sensitive and understanding
**Recruitment**	How were you approached to take part in the TIC TOC study?<br><br>What did you think about the way the study was introduced?<br><br>What did you understand about the study?<br><br>What questions did you have? Did you receive answers you understood?	Which member of staff, how approached(surgeon, specialist nurse)
**Specific understanding**	What did you understand about reintroducing your own blood?<br><br>What did you understand by donor blood transfusion?	Which method did you think was safest?
**Involvement of family and friends**	Did you ask anyone else for their opinions?<br><br>If yes, who were they?<br><br>What was their opinion?	Explore any negative responses from family and friends<br><br>Explore any positive responses from family and friends
**Decision process**	What things did you think about when deciding if you were going to take part?	Barriers<br><br>Factors that stopped the woman taking part (fear, overwhelmed by potential cancer diagnosis, chance would get cell salvage anyway (some

For peer review only

		sites), lack of understanding, unable to read research literature)<br><br>Facilitators<br><br>Factors that encouraged her to take part (trust of surgeon, research staff, feeling obligated, fear, distrust of donated blood or salvaged blood)
**Research processes**	When you came to the first clinic to see your consultant, how were you treated in the research part of your appointment?<br><br>Tell me what you felt about the specialist nurse asking you if you wanted to take part in the TIC TOC study?<br><br>What did you think about the timing of being recruited to the study?<br><br>What did you think about the questionnaires?	Check woman's talk is about the research.<br><br>Woman may want to talk about their cancer experience – allow it.<br><br>Baseline questionnaires only
**Allocation**	Which group do you think you were allocated?<br><br>Why?	Do not say which
**Information about next appointments**	As part of your normal care, you will be followed up by your consultant or his/her team. As part of the research study you will receive some further postal questionnaires.	

For peer review only

	Can I contact you again in about 6 months to see what you think about the postal follow-up?	

Second qualitative interview (three months after first, by telephone)

**Topic**	**Questions**	**Prompts**
**Opening question**	Since we last spoke, how have you been getting on?<br><br>I have a few questions to ask you about your experience of taking part in the TIC TOC study.	May not be feeling well.<br><br>May be on chemotherapy treatment.<br><br>Be sensitive and understanding
**Research process:**<br><br>**follow-up questionnaires**	Where did you complete your questionnaires?<br><br>Did you have help to complete the questionnaires?<br><br>What did you like about the telephone/postal follow up?<br><br>What didn't you like about the telephone/postal follow up?<br><br>Was there anything that could be improved?<br><br>Did you know who to contact if you did not wish to keep taking part?	Did the woman know how to make a complaint?

For peer review only

	What did you think about the questionnaires asking you what health services you had used?	(probe questionnaires by telephone)<br><br>Check view about the number of questionnaires and clarity of questions<br><br>Check for questionnaire burden
**Allocation**	Which group do you think you were allocated?<br><br>Why?	**Do not say which.**<br><br>**The woman will receive notification about the allocation at the end of the study.**
	Thank you for taking part in the research study that will help inform a larger study.<br><br>Wish well for the future.	

For peer review only

STANDARD PROTOCOL ITEMS: RECOMMENDATIONS FOR INTERVENTIONAL TRIALS

SPIRIT 2013 Checklist: Recommended items to address in a clinical trial protocol and related documents*

Section/item	Item No	Description	Addressed on page number
**Administrative information**			
Title	1	Descriptive title identifying the study design, population, interventions, and, if applicable, trial acronym	_____1________
Trial registration	2a	Trial identifier and registry name. If not yet registered, name of intended registry	_____2_____
	2b	All items from the World Health Organization Trial Registration Data Set	1-23, as applicable
Protocol version	3	Date and version identifier	9 Oct 2017, not included
Funding	4	Sources and types of financial, material, and other support	______22_______
Roles and responsibilities	5a	Names, affiliations, and roles of protocol contributors	______1, 21-22__
	5b	Name and contact information for the trial sponsor	______22_______
	5c	Role of study sponsor and funders, if any, in study design; collection, management, analysis, and interpretation of data; writing of the report; and the decision to submit the report for publication, including whether they will have ultimate authority over any of these activities	_____22_____
	5d	Composition, roles, and responsibilities of the coordinating centre, steering committee, endpoint adjudication committee, data management team, and other individuals or groups overseeing the trial, if applicable (see Item 21a for data monitoring committee)	_____14________

For peer review only

Introduction

Background and rationale	6a	Description of research question and justification for undertaking the trial, including summary of relevant studies (published and unpublished) examining benefits and harms for each intervention	______4-6_____
	6b	Explanation for choice of comparators	____4, 5_______
Objectives	7	Specific objectives or hypotheses	_____6________
Trial design	8	Description of trial design including type of trial (eg, parallel group, crossover, factorial, single group), allocation ratio, and framework (eg, superiority, equivalence, noninferiority, exploratory)	______6, 7______

Methods: Participants, interventions, and outcomes

Study setting	9	Description of study settings (eg, community clinic, academic hospital) and list of countries where data will be collected. Reference to where list of study sites can be obtained	_____7________
Eligibility criteria	10	Inclusion and exclusion criteria for participants. If applicable, eligibility criteria for study centres and individuals who will perform the interventions (eg, surgeons, psychotherapists)	_____ 8_______
Interventions	11a	Interventions for each group with sufficient detail to allow replication, including how and when they will be administered	_____9_______
	11b	Criteria for discontinuing or modifying allocated interventions for a given trial participant (eg, drug dose change in response to harms, participant request, or improving/worsening disease)	____9_________
	11c	Strategies to improve adherence to intervention protocols, and any procedures for monitoring adherence (eg, drug tablet return, laboratory tests)	_____N/A_______
	11d	Relevant concomitant care and interventions that are permitted or prohibited during the trial	_____9-10______
Outcomes	12	Primary, secondary, and other outcomes, including the specific measurement variable (eg, systolic blood pressure), analysis metric (eg, change from baseline, final value, time to event), method of aggregation (eg, median, proportion), and time point for each outcome. Explanation of the clinical relevance of chosen efficacy and harm outcomes is strongly recommended	_____10, 11_____
Participant timeline	13	Time schedule of enrolment, interventions (including any run-ins and washouts), assessments, and visits for participants. A schematic diagram is highly recommended (see Figure)	_____12________

For peer review only - http://bmjopen.bmj.com/site/about/guidelines.xhtml

Sample size	14	Estimated number of participants needed to achieve study objectives and how it was determined, including clinical and statistical assumptions supporting any sample size calculations	____14_______
Recruitment	15	Strategies for achieving adequate participant enrolment to reach target sample size	_____N/A______

Methods: Assignment of interventions (for controlled trials)

Allocation:			
Sequence generation	16a	Method of generating the allocation sequence (eg, computer-generated random numbers), and list of any factors for stratification. To reduce predictability of a random sequence, details of any planned restriction (eg, blocking) should be provided in a separate document that is unavailable to those who enrol participants or assign interventions	_____8________
Allocation concealment mechanism	16b	Mechanism of implementing the allocation sequence (eg, central telephone; sequentially numbered, opaque, sealed envelopes), describing any steps to conceal the sequence until interventions are assigned	_____8________
Implementation	16c	Who will generate the allocation sequence, who will enrol participants, and who will assign participants to interventions	______7, 8______
Blinding (masking)	17a	Who will be blinded after assignment to interventions (eg, trial participants, care providers, outcome assessors, data analysts), and how	____10_______
	17b	If blinded, circumstances under which unblinding is permissible, and procedure for revealing a participant's allocated intervention during the trial	____N/A_______

Methods: Data collection, management, and analysis

Data collection methods	18a	Plans for assessment and collection of outcome, baseline, and other trial data, including any related processes to promote data quality (eg, duplicate measurements, training of assessors) and a description of study instruments (eg, questionnaires, laboratory tests) along with their reliability and validity, if known. Reference to where data collection forms can be found, if not in the protocol	____12, 13______
	18b	Plans to promote participant retention and complete follow-up, including list of any outcome data to be collected for participants who discontinue or deviate from intervention protocols	_____13________

For peer review only - http://bmjopen.bmj.com/site/about/guidelines.xhtml

Data management	19	Plans for data entry, coding, security, and storage, including any related processes to promote data quality (eg, double data entry; range checks for data values). Reference to where details of data management procedures can be found, if not in the protocol	____12,13_____
Statistical methods	20a	Statistical methods for analysing primary and secondary outcomes. Reference to where other details of the statistical analysis plan can be found, if not in the protocol	____15______
	20b	Methods for any additional analyses (eg, subgroup and adjusted analyses)	_____N/A_______
	20c	Definition of analysis population relating to protocol non-adherence (eg, as randomised analysis), and any statistical methods to handle missing data (eg, multiple imputation)	_______N/A______
**Methods: Monitoring**			
Data monitoring	21a	Composition of data monitoring committee (DMC); summary of its role and reporting structure; statement of whether it is independent from the sponsor and competing interests; and reference to where further details about its charter can be found, if not in the protocol. Alternatively, an explanation of why a DMC is not needed	_____14_______
	21b	Description of any interim analyses and stopping guidelines, including who will have access to these interim results and make the final decision to terminate the trial	_____N/A_______
Harms	22	Plans for collecting, assessing, reporting, and managing solicited and spontaneously reported adverse events and other unintended effects of trial interventions or trial conduct	__13-14________
Auditing	23	Frequency and procedures for auditing trial conduct, if any, and whether the process will be independent from investigators and the sponsor	_____N/A_______
**Ethics and dissemination**			
Research ethics approval	24	Plans for seeking research ethics committee/institutional review board (REC/IRB) approval	_______2______
Protocol amendments	25	Plans for communicating important protocol modifications (eg, changes to eligibility criteria, outcomes, analyses) to relevant parties (eg, investigators, REC/IRBs, trial participants, trial registries, journals, regulators)	_____N/A_______

Consent or assent	26a	Who will obtain informed consent or assent from potential trial participants or authorised surrogates, and how (see Item 32)	____7_________
	26b	Additional consent provisions for collection and use of participant data and biological specimens in ancillary studies, if applicable	____N/A________
Confidentiality	27	How personal information about potential and enrolled participants will be collected, shared, and maintained in order to protect confidentiality before, during, and after the trial	____12 -13_____
Declaration of interests	28	Financial and other competing interests for principal investigators for the overall trial and each study site	____22__________
Access to data	29	Statement of who will have access to the final trial dataset, and disclosure of contractual agreements that limit such access for investigators	_____19________
Ancillary and post-trial care	30	Provisions, if any, for ancillary and post-trial care, and for compensation to those who suffer harm from trial participation	______19_______
Dissemination policy	31a	Plans for investigators and sponsor to communicate trial results to participants, healthcare professionals, the public, and other relevant groups (eg, via publication, reporting in results databases, or other data sharing arrangements), including any publication restrictions	______19_______
	31b	Authorship eligibility guidelines and any intended use of professional writers	______N/A______
	31c	Plans, if any, for granting public access to the full protocol, participant-level dataset, and statistical code	______N/A______
**Appendices**			
Informed consent materials	32	Model consent form and other related documentation given to participants and authorised surrogates	Appendix pp5 -14
Biological specimens	33	Plans for collection, laboratory evaluation, and storage of biological specimens for genetic or molecular analysis in the current trial and for future use in ancillary studies, if applicable	_______N/A_____

*It is strongly recommended that this checklist be read in conjunction with the SPIRIT 2013 Explanation & Elaboration for important clarification on the items. Amendments to the protocol should be tracked and dated. The SPIRIT checklist is copyrighted by the SPIRIT Group under the Creative Commons "Attribution-NonCommercial-NoDerivs 3.0 Unported" license.
